# Supplementary material for: Whole-genome amplification in double-digest RADseq results in adequate libraries but fewer sequenced loci
Source: PeerJ. 2018 Jul 17;6:e5089. doi: 10.7717/peerj.5089 (PMC6054070; doi:10.7717/peerj.5089)
Supplement: Supplemental Information 6 [file peerj-06-5089-s006.pdf]

Whole-genome amplification

REVISED BY B. MEDEIROS 19-MAR-2015. Changed from 20uL to 15uL reactions

This protocol describes how to perform whole-genome amplification using the Repli-g mini kit. The protocol was modified from the manufacturer to do 15 uL reactions, instead of the suggested 50 uL. This enables a kit to be used in 3.3 times more reactions than the manufacturer recommendation.

Part requirements

REPLI-g Mini kit (Qiagen 150025 (~$600 + $100 shipping) or 150023)
Nuclease-free water
Strip tubes

1 – Preparing the kit
Buffer DLB must be reconstituted before the kit can be used. This is done by adding 500uL of nuclease-free water to the corresponding tube. Vortex and quick spin after adding water. Buffer DLB can be stored for up to 6 months in the freezer.

2 – Preparing reaction sheet
Use the template excel file to make a table relating samples their positions in the strip tubes.

3 – Preparing buffers for the reaction.
1. Thaw DNA polymerase on ice.
2. Thaw other reagents at room temperature.
3. Vortex all buffers before continuing.
4. Label three tubes as follow: D1, N1 and * (for master mix)
5. Label strip tubes indicating sample locations.
6. Prepare sufficient Buffer D1 and Buffer N1 for the total number of reactions (see template excel spreadsheet). If doing more than 16 reactions, prepare buffers only for 16 reactions at a time. Vortex after mixing. Quantities per reaction:
    a. Buffer D1: 0.34 uL of Buffer DLB to 1.20 uL of water
    b. Buffer N1: 0.45 uL of stop solution to 2.55 uL of water
7. Add 1.5 uL of each template DNA to the BOTTOM of the corresponding tube.

If doing more than 8 reactions, repeat steps 8-16 for each strip tube separately. Place each strip tube at the thermocycler as soon as you are done with it.

8. Add 1.5 uL of Buffer D1 to the SIDE of each tube (so all tubes will mix at the same time).
9. Place tube caps, mix by vortexing and quick spin.
10. Incubate the samples at room temperature for 3 min.
11. Add 3 uL of buffer N1 to the SIDE of each tube.

12. Place tube caps, mix by vortexing and quick spin (so all tubes will mix at the same time).
13. Vortex the reaction buffer tube for 10 s to dissolve any precipitate that might have formed.
14. Prepare a master mix. Keep it on ice. Mix by flickering and then quick spin. Quantities per reaction:
    a. Master Mix: 0.30 uL of polymerase to 8.70 uL of reaction buffer.
15. Add 9 uL of master mix to 6 uL of denatured DNA (the product of previous reactions).
16. Seal the plate and put it in the thermocycler. Thermocycler program:
    a. Temperature of lid: 70ºC.
    b. Incubate at 30ºC for 16h.
    c. Incubate at 65ºC for 3 min.
    d. 4ºC forever.
17. Quantitate the samples and use directly for restriction enzyme digestion in RAD tags. No need to clean. For other uses, see manufacturer manual.
18. Samples can be stored at 4ºC for short-term and -20ºC for long-term.
