# Supplementary material for: Whole-genome amplification in double-digest RADseq results in adequate libraries but fewer sequenced loci
Source: PeerJ. 2018 Jul 17;6:e5089. doi: 10.7717/peerj.5089 (PMC6054070; doi:10.7717/peerj.5089)
Supplement: Supplemental Information 7 [file peerj-06-5089-s007.pdf]

Article S2 - Effects of whole genome amplification

Contents

Does the amount of input DNA into the MDA reaction affect number of loci assembled? 2

Assessing nontemplated amplification 6
Aligning reads and clusters to references 6
Comparing same samples with and without whole-genome amplification 26

Comparing MDA and gDNA for number of loci, heterozygosity and GC bias 30
Does whole-genome amplification reduce the number of loci obtained in comparison to gDNA libraries? 30
What is the effect of MDA on variance of read depth among loci? 34
Does MDA interfere with heterozygosity? 38
4 - does WGA bias the GC content? 47

Are errors and biases caused by MDA large enough to impair analyses? 50

Are the loci sequenced with MDA are biased subset of loci sequenced with gDNA libraries? 58

This notebook contains code to reproduce analyses and graphs from Medeiros & Farrell (PeerJ, 2018).

First, we start by reading packages, tables, functions, etc.

```
rm(list=ls())
library(ggplot2)
library(lme4)
library(lmerTest)
library(RColorBrewer)
library(ggthemes)
library(ggdendro)
library(MDMR)
library(scales)
library(gridExtra)
library(plyr)
library(dplyr)
library(knitr)

#this function will be important for plots
make_WGA_labels = function(labels){
  labels = lapply(labels, as.character)
  labels = lapply(labels, function(x) if (x == 'TRUE') {return('MDA')} else {return('gDNA')})
  return(labels)}

#function to do logit transformation
logit = function(vec){
  log(vec/(1-vec))
}

#reverse logit function
inv_logit = function(vec){
  exp(vec) / (exp(vec) + 1)
}
```

```
sample_info_all = read.csv('sample_info_new_WGA.csv')

sample_info_all$pool = factor(sample_info_all$pool)
#sample_info_all$WGA = factor(sample_info_all$WGA,levels=c('FALSE','TRUE'),ordered = T)

#this records the total number of loci in the final dataset for each taxon
Nfinal = c('Anchylorhynchus' = 42240,
           'Andranthobius' = 17769,
           'Celetes_impar' = 27624,
           'Microstrates_bondari' = 17344,
           'Microstrates_ypsilon' = 17712)

#this is the fraction of loci in the final dataset recovered for a sample
sample_info_all$Pfinal = sample_info_all$N_loci_s7/Nfinal[as.character(sample_info_all$taxon)]

#let's also read statistics from ipyrad s3 and merge them with sample info table
s3_stats = read.table('s3_cluster_stats.txt',header = T,sep = '')
s3_stats$samplename_ipyrad = rownames(s3_stats)

sample_info_all = sample_info_all %>% left_join(s3_stats)
```

```
## Warning: Column `samplename_ipyrad` joining factor and character vector,
## coercing into character vector
```

```
head(sample_info_all)
```

sample	genus	species	taxon	population	pool	WGA	WGA_input_ng	sampler
BdM1231	Anchylorhynchus	trapezicollis	Anchylorhynchus	P1	5	FALSE	NA	BdM12
BdM1231	Anchylorhynchus	trapezicollis	Anchylorhynchus	P1	27	TRUE	305.613070	BdM12
BdM1232	Anchylorhynchus	trapezicollis	Anchylorhynchus	P1	5	FALSE	NA	BdM12
BdM1232	Anchylorhynchus	trapezicollis	Anchylorhynchus	P1	27	TRUE	322.723674	BdM12
BdM1233	Anchylorhynchus	trapezicollis	Anchylorhynchus	P1	5	TRUE	8.611418	BdM12
BdM1233	Anchylorhynchus	trapezicollis	Anchylorhynchus	P1	18	TRUE	8.611418	BdM12

```
knitr::opts_chunk$set(warning=FALSE)
knitr::opts_chunk$set(tidy.opts=list(width.cutoff=60),tidy=TRUE)
```

Does the amount of input DNA into the MDA reaction affect number of loci assembled?

Here we will test whether using smaller amounts of template DNA in the MDA reaction decreases the number of loci that can be assembled.

We start with a model with number of loci as response and the following predictors: * Amount of inpout genomic DNA in the MDA reactions (ng) * log-transformed number of reads.

We will use R function step to try to simplify the model and will report the final model.

Below are the diagnostic plots and the output for the chosen model. In the best model, number of loci increases with number of reads and decreases with quantity of DNA:

```
temp_data = sample_info_all[c("clusters_hidepth", "WGA_input_ng",
    "taxon", "pool", "reads_passed_filter")]
temp_data$log_reads = log(sample_info_all$reads_passed_filter)
temp_data$log_clusters = log(sample_info_all$clusters_hidepth)
temp_data$N_clusters = sample_info_all$clusters_hidepth

temp_data = temp_data[!is.na(temp_data$WGA_input_ng), ]

ng_model_full = lmer(N_clusters ~ WGA_input_ng * log_reads +
    (1 | taxon) + (1 | pool), data = temp_data, REML = TRUE)

step_out = step(ng_model_full, reduce.random = FALSE)

ng_model = get_model(step_out)
plot(ng_model)
```

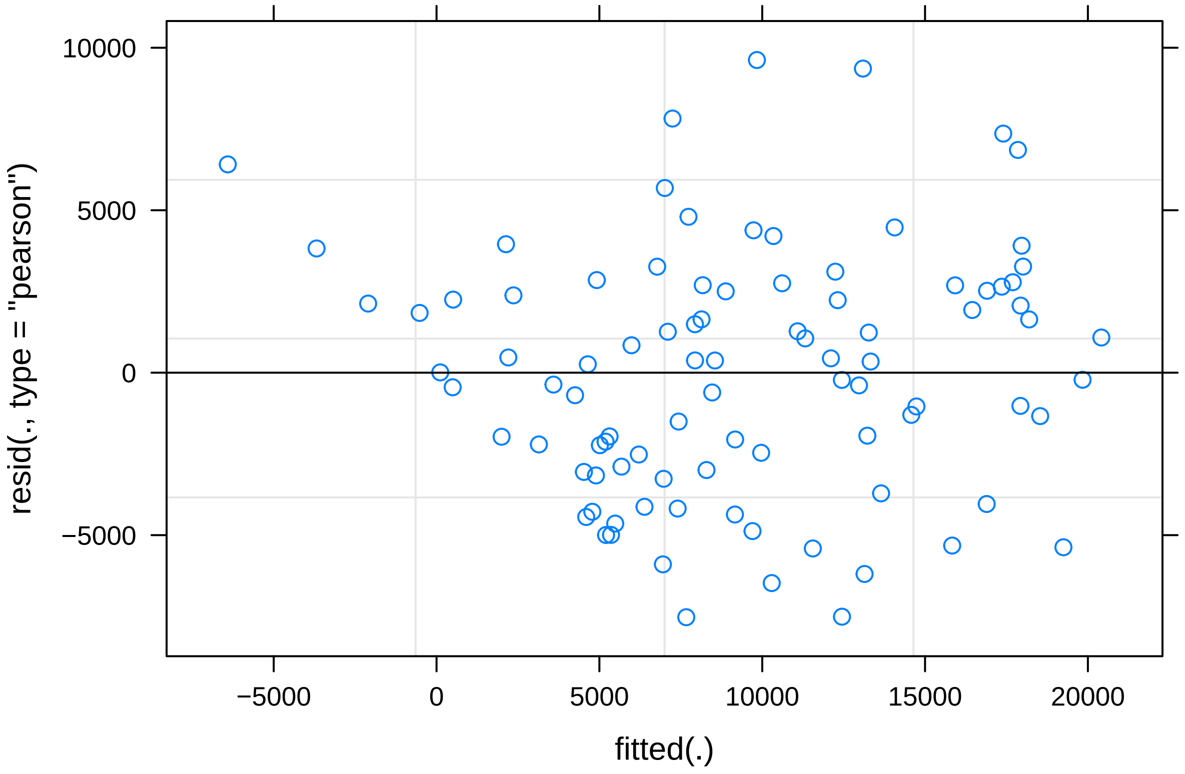


```
qqnorm(resid(ng_model))
```

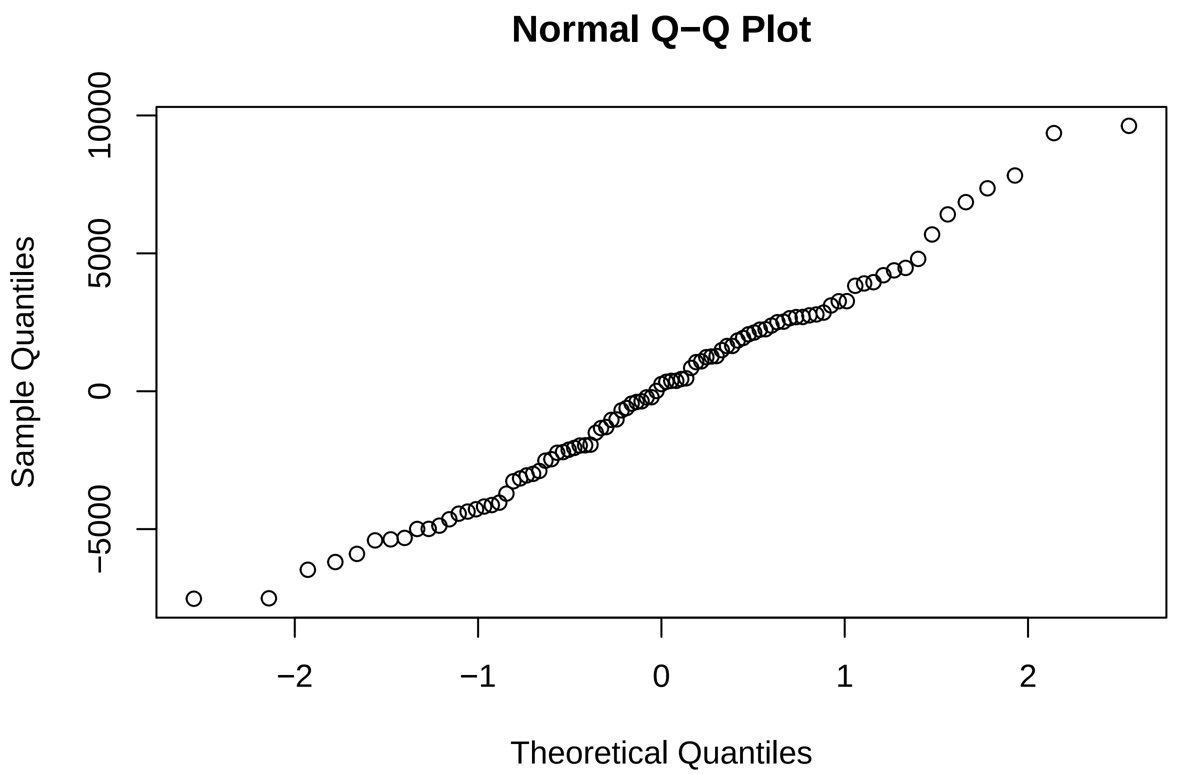


```
summary(ng_model)
```

```
## Linear mixed model fit by REML. t-tests use Satterthwaite's method [
## lmerModLmerTest]
## Formula: N_clusters ~ WGA_input_ng * log_reads + (1 | taxon) + (1 | pool)
##    Data: temp_data
##
## REML criterion at convergence: 1780.4
##
## Scaled residuals:
##      Min       1Q   Median       3Q      Max
## -1.88806 -0.72552  0.06512  0.63266  2.41430
##
## Random effects:
##  Groups   Name        Variance Std.Dev.
##  pool     (Intercept)  2793696 1671
##  taxon    (Intercept) 13719767 3704
##  Residual             15884949 3986
## Number of obs: 93, groups:  pool, 13; taxon, 5
##
## Fixed effects:
##                         Estimate Std. Error        df t value Pr(>|t|)
## (Intercept)            -6624.925   6550.987    58.893  -1.011   0.3160
## WGA_input_ng            -227.423     53.492    85.279  -4.252 5.41e-05 ***
## log_reads               1061.323    495.624    56.713   2.141   0.0366 *
## WGA_input_ng:log_reads    18.213      4.083    84.778   4.461 2.49e-05 ***
## ---
## Signif. codes:  0 '***' 0.001 '**' 0.01 '*' 0.05 '.' 0.1 ' ' 1
##
## Correlation of Fixed Effects:
```

```
##              (Intr) WGA_n_ lg_rds
## WGA_inpt_ng -0.605
## log_reads   -0.956  0.617
## WGA_npt_n:_  0.608 -0.995 -0.629
## fit warnings:
## Some predictor variables are on very different scales: consider rescaling
```

A graph with data and model predictions makes it more clear:

```
line_seq = log(10^seq(2, 6.3, 0.01))
line_df = data.frame(log_reads = line_seq, WGA_input_ng = rep(c(50,
    100, 300), each = length(line_seq)))
predicted = predict(ng_model, line_df, re.form = NA)
prediction = cbind(line_df, x = exp(line_df$log_reads)/1000,
    y = predicted)



p = ggplot(sample_info_all[!is.na(sample_info_all$WGA_input_ng),
    ]) + geom_point(aes(x = reads_passed_filter/1000, y = clusters_hidepth/1000,
    colour = WGA_input_ng)) + geom_line(data = prediction, mapping = aes(x = x,
    y = y/1000, colour = WGA_input_ng, group = WGA_input_ng)) +
    scale_colour_gradientn(name = "Input DNA (ng)", colours = c(brewer.pal(n = 5,
        name = "BrBG")[1], "#c1c18f", brewer.pal(n = 5, name = "BrBG")[5],
        "#018571"), values = rescale(c(0, 100, 200, 357))) +
    scale_y_continuous(limits = c(0, 25)) + # scale_y_log10() +
theme_tufte() + theme(panel.border = element_rect(colour = "black",
    fill = NA)) + xlab("Reads passing filter (thousands)") +
    ylab("Loci assembled (thousands)")

print(p)
```

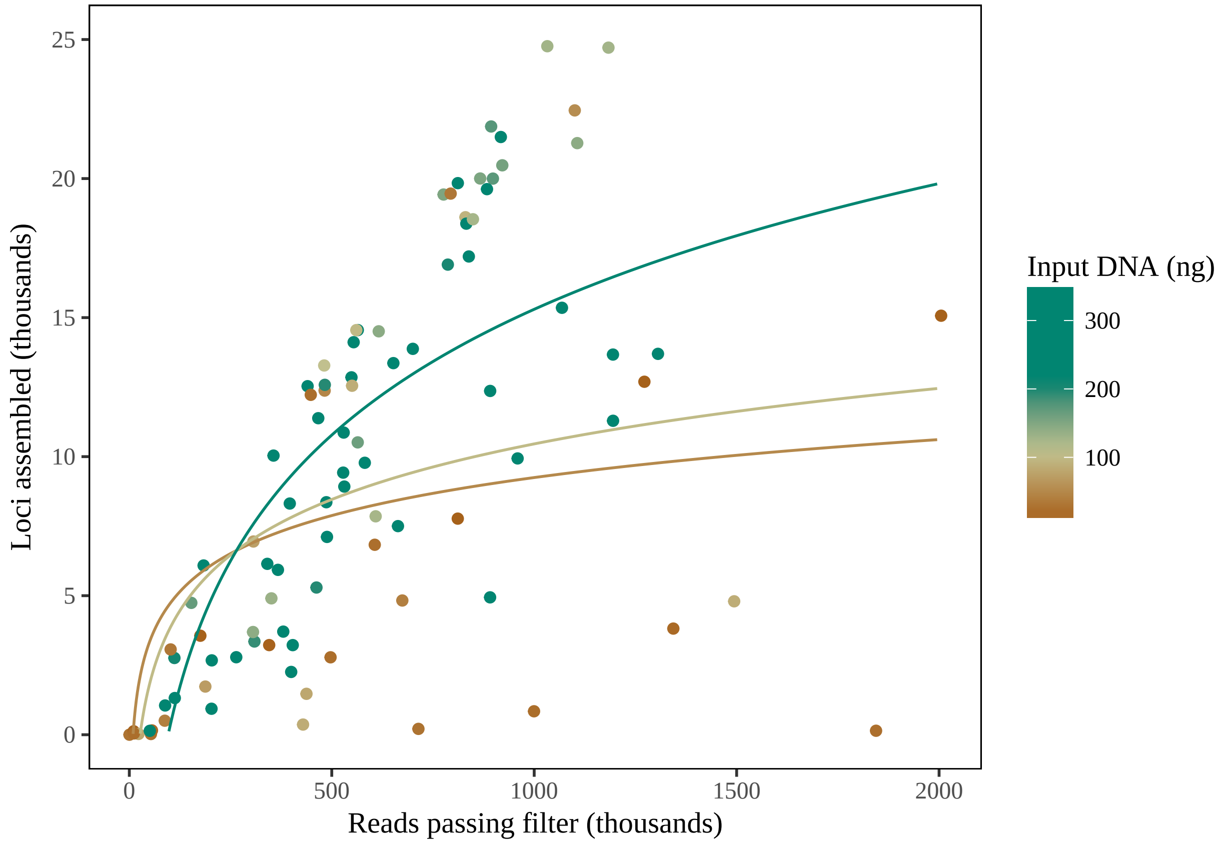


```
# now plotting to a pdf

pdf(file = "plots/fig_quantWGA.pdf", width = 7, height = 3.5,
    useDingbats = F)
print(p)
dev.off()
```

```
## pdf
##   2
```

Assessing nontemplated amplification

Aligning reads and clusters to references

Contamination is a potential issue with whole-genome amplification. Therefore, it is important to assess whether there is significant contamination in the samples here.

For that, we used bowtie2 to align reads and also assembled clusters to reference sequences from potential contaminants. These consisted of the repeat-masked human genome (from [ftp://ftp.ncbi.nlm.nih.gov/genomes/all/GCA/000/001/405/GCA_000001405.25_GRCh38.p10/GCA_000001405.25_GRCh38.p10_genomic.fna.gz], replacing lower-case for Ns) and of reference sequences for each beetle taxon in our dataset. In the absence of reference genomes, the reference sequences were generated from final loci obtained for all libraries which did not undergo whole-genome amplification, dereplicated independently for each taxon using cd-hit at the 95% similarity level. For the reference dataset, we used all libraries available that did not undergo whole-genome amplification, including libraries that were not duplicated and libraries that yielded few clusters.

We then parsed bowtie results and, for each library, counted the number of matches to (1) human genome, (2) to the correct taxon, (3) to incorrect taxa and (4) also sequences that did not match to anything.

Before analysing, let's prepare the data. We will make a new dataframe containing only samples with both gDNA and MDA libraries and at least 100 assembled loci.

We will create the following data frames:

- sample_info_sing * will store information for all samples that will have only one library considered
- sample_info_dup * will store information for all samples with two libraries: one with WGA and one without.
- cont_reads * will store counts of reads aligned by bowtie to each reference taxon
- cont_clusters * is the same as above, but queries consisted of clusters with depth > 7
- cont_final * is the same as above, but queries consisted of loci in the final dataset

```
sample_info = sample_info_all[!sample_info_all$excluded_dueto_less_than_100_loci,
    ]  #remove samples with <100 clusters

# now remove empty factor levels
categorical = sapply(sample_info, is.factor)
sample_info[categorical] = lapply(sample_info[categorical], factor)

samples = levels(sample_info$sample)

duplicate_samples = samples[sapply(samples, function(x) {
    sum(sample_info$sample == x, na.rm = T)
}) > 1]

duplicate_2methods_samples = as.character(duplicate_samples[sapply(duplicate_samples,
    function(x) {
        xor(as.logical(sample_info$WGA[sample_info$sample ==
            x][1]), as.logical(sample_info$WGA[sample_info$sample ==
            x][2]))
    })])

single_samples = setdiff(samples, duplicate_samples)

dup_single_method = setdiff(duplicate_samples, duplicate_2methods_samples)


sample_info_dup = sample_info[sample_info$sample %in% duplicate_2methods_samples,
    ]
sample_info_dup = sample_info_dup[order(sample_info_dup$sample,
    sample_info_dup$WGA), ]
categorical = sapply(sample_info_dup, is.factor)
sample_info_dup[categorical] = lapply(sample_info_dup[categorical],
    factor)

sample_info_sing = sample_info[sample_info$sample %in% single_samples,
    ]
sample_info_sing = sample_info_sing[order(sample_info_sing$sample,
    sample_info_sing$WGA), ]
categorical = sapply(sample_info_sing, is.factor)
sample_info_sing[categorical] = lapply(sample_info_sing[categorical],
```

```
    factor)


for (sample in dup_single_method) {
    temp_data = sample_info[sample_info$sample == sample, ]
    sample_info_sing = rbind(sample_info_sing, temp_data[temp_data$N_loci_s7 ==
        max(temp_data$N_loci_s7), ])
}


cont_reads = read.delim("contamination_reads_counts.txt", row.names = 1)
cont_reads = t(apply(cont_reads, 1, function(x) {
    x/sum(x)
}))

cont_clusters = read.delim("contamination_clusters_counts.txt",
    row.names = 1)
cont_clusters = t(apply(cont_clusters, 1, function(x) {
    x/sum(x)
}))

cont_final = read.delim("contamination_final_counts.txt", row.names = 1)
cont_final = t(apply(cont_final, 1, function(x) {
    x/sum(x)
}))
```

Nontemplated amplification at the read level

A plot of the proportion of raw reads matching each of the categories above. After we got the first reviews back for the manuscript, ggthemes updated colors for the few theme. If running this code with ggthemes v. 3.5.0 or above, you might end up with a different color palette than the one published:

```
cont_reads_df = data.frame(sample = NULL, ipyrad_sample = NULL,
    taxon = NULL, match = NULL, frequency = NULL, pool = NULL)

for (sample in unique(row.names(cont_reads))) {
    if (sample %in% sample_info_dup$samplename_ipyrad) {
        freqs = rep(NA, 4)
        names(freqs) = c("correct", "incorrect", "Homo", "none")
        taxon = as.character(sample_info_dup$taxon[sample_info_dup$samplename_ipyrad ==
            sample])
        freqs["correct"] = cont_reads[sample, taxon]
        freqs["incorrect"] = sum(cont_reads[sample, !grepl(paste(c(as.character(taxon),
            "Homo", "none"), collapse = "|"), colnames(cont_reads))])
        freqs["Homo"] = cont_reads[sample, "Homo"]
        freqs["none"] = cont_reads[sample, "none"]
        cont_reads_df = rbind(cont_reads_df, data.frame(sample = strsplit(sample,
            "pool")[[1]][1], ipyrad_sample = sample, taxon = taxon,
            match = names(freqs), frequency = freqs, pool = strsplit(sample,
                "pool")[[1]][2]))
    }
}
row.names(cont_reads_df) = NULL
```

```r
cont_reads_df = merge(cont_reads_df, sample_info_dup[c("samplename_ipyrad",
    "clusters_hidepth", "WGA")], by.x = "ipyrad_sample", by.y = "samplename_ipyrad")

cont_reads_df$match = factor(as.character(cont_reads_df$match),
    ordered = T, levels = c("correct", "incorrect", "Homo", "none"))

cont_reads_df$Nloci = sapply(cont_reads_df$ipyrad_sample, function(x) {
    sample_info$clusters_hidepth[sample_info$samplename_ipyrad ==
        as.character(x)]
})

cont_reads_df$Nreads = sapply(cont_reads_df$ipyrad_sample, function(x) {
    sample_info$reads_passed_filter[sample_info$samplename_ipyrad ==
        as.character(x)]
})

# this will make sure samples in the graph dataset are
# ordered by taxon
cont_reads_df$sample = factor(as.character(cont_reads_df$sample),
    ordered = T, levels = unique(cont_reads_df$sample[order(as.character(cont_reads_df$taxon))]))



p1 = ggplot(cont_reads_df) + geom_bar(aes(x = sample, weight = frequency,
    fill = match)) + facet_grid(WGA ~ ., labeller = labeller(WGA = make_WGA_labels)) +
    theme_tufte() + scale_fill_few(palette = "medium", name = "",
    labels = setNames(c("Correct", "Incorrect", "Human", "No match"),
        c("correct", "incorrect", "Homo", "none"))) + theme(axis.text.x = element_blank(),
    axis.ticks.x = element_blank()) + xlab("Samples") + ylab("Reads passing filter")

print(p1)
```

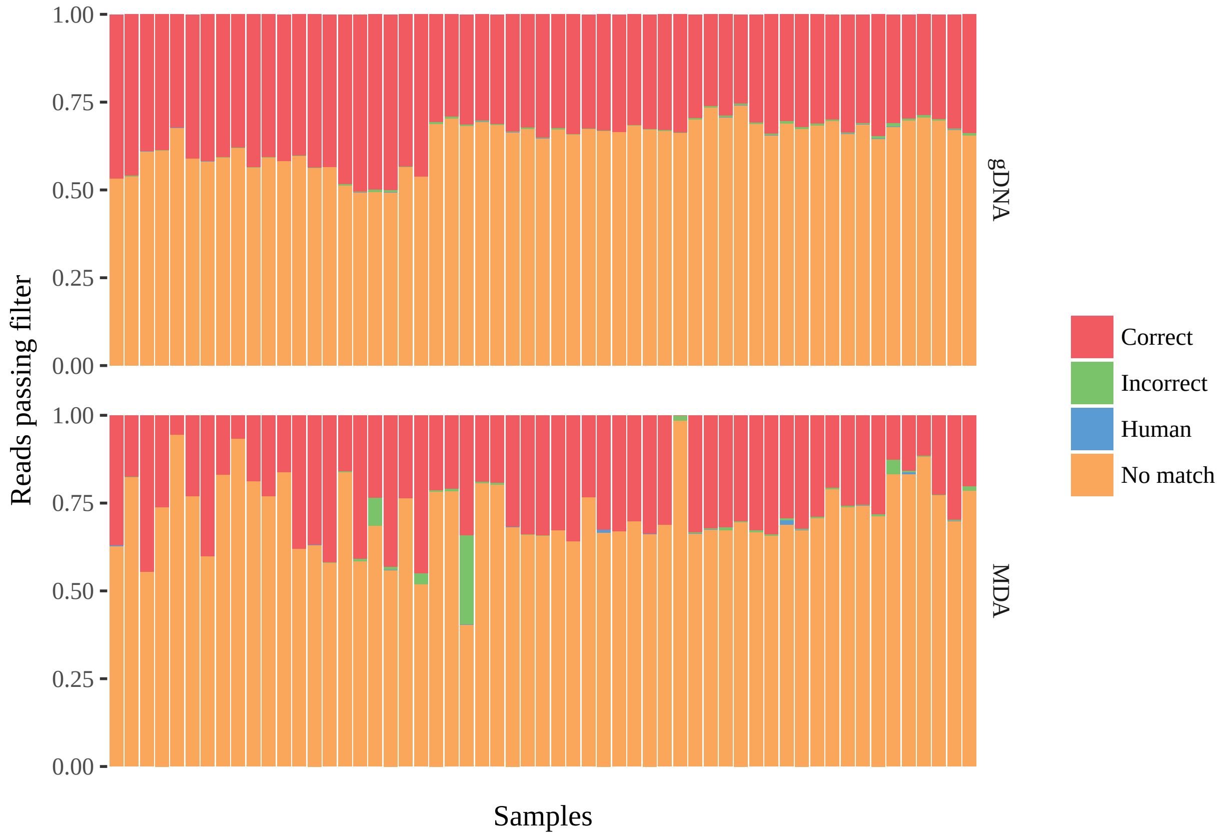

There is some background incorrect matches because of the way the reference set was built: some proportion of reads might match to a conserved region in a closely related species (e. g. the two species of Microstrates). Comparing the same samples with and without MDA, we can see that MDA causes widespread increase in proportion of **no match** and occasionally in **incorrect**. We will investigate this further below.

Now we will do some statistics.

First, is the proportion of incorrect matches increased under MDA, controlling for and number of reads?

It seems it is, and the effect is stronger when we have fewer reads for a sample.

Diagnostic plots and R output:

```
incorrect_df = cont_reads_df[cont_reads_df$match == "incorrect",
    ]
incorrect_df$frequency = cont_reads_df$frequency[cont_reads_df$match ==
    "incorrect"] + cont_reads_df$frequency[cont_reads_df$match ==
    "Homo"]
incorrect_df$WGA = factor(incorrect_df$WGA, levels = c("FALSE",
    "TRUE"))  #this is just to nicely order the output of the model summary
incorrect_df$logit_freq = logit(incorrect_df$frequency)
incorrect_df$log_reads = log(incorrect_df$Nreads)

# full model
cont_reads_model_full = lmer(logit_freq ~ WGA * log_reads + (1 |
    taxon/sample), data = incorrect_df, REML = TRUE)

step_out = step(cont_reads_model_full, reduce.random = FALSE)
step_out
```

```
## Backward reduced random-effect table:
##
##                       Eliminated npar  logLik    AIC    LRT Df Pr(>Chisq)
## <none>                             7 -193.67 401.33
## (1 | sample:taxon)           0     6 -198.89 409.78 10.449  1   0.001227 **
## (1 | taxon)                  0     6 -201.80 415.60 16.264  1  5.509e-05 ***
## ---
## Signif. codes:  0 '***' 0.001 '**' 0.01 '*' 0.05 '.' 0.1 ' ' 1
##
## Backward reduced fixed-effect table:
## Degrees of freedom method: Satterthwaite
##
##                  Eliminated Sum Sq Mean Sq NumDF  DenDF F value  Pr(>F)
## WGA:log_reads             1 0.0143  0.0143     1 81.691  0.0155 0.90112
## WGA                       2 1.5393  1.5393     1 66.326  1.7053 0.19611
## log_reads                 0 6.3935  6.3935     1 66.659  6.6703 0.01201 *
## ---
## Signif. codes:  0 '***' 0.001 '**' 0.01 '*' 0.05 '.' 0.1 ' ' 1
##
## Model found:
## logit_freq ~ log_reads + (1 | taxon/sample)
```

```r
cont_reads_model = get_model(step_out)

plot(cont_reads_model)
```

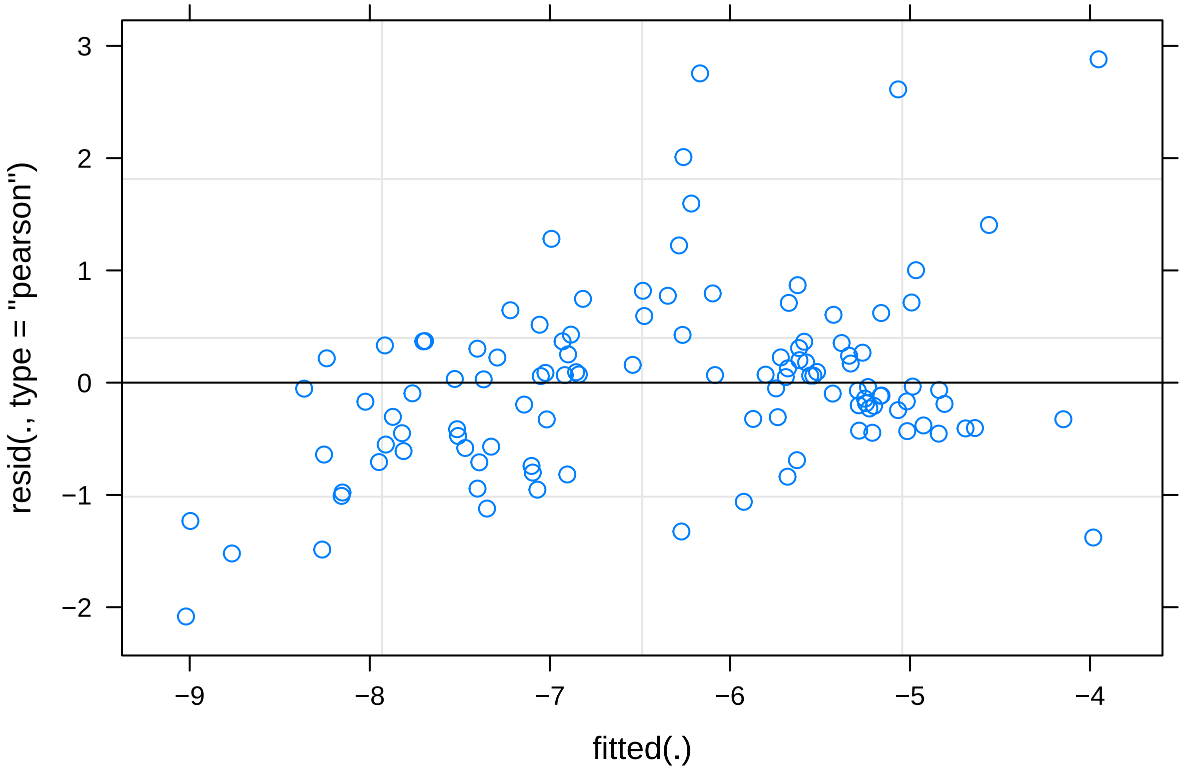


```r
qqnorm(resid(cont_reads_model))
```

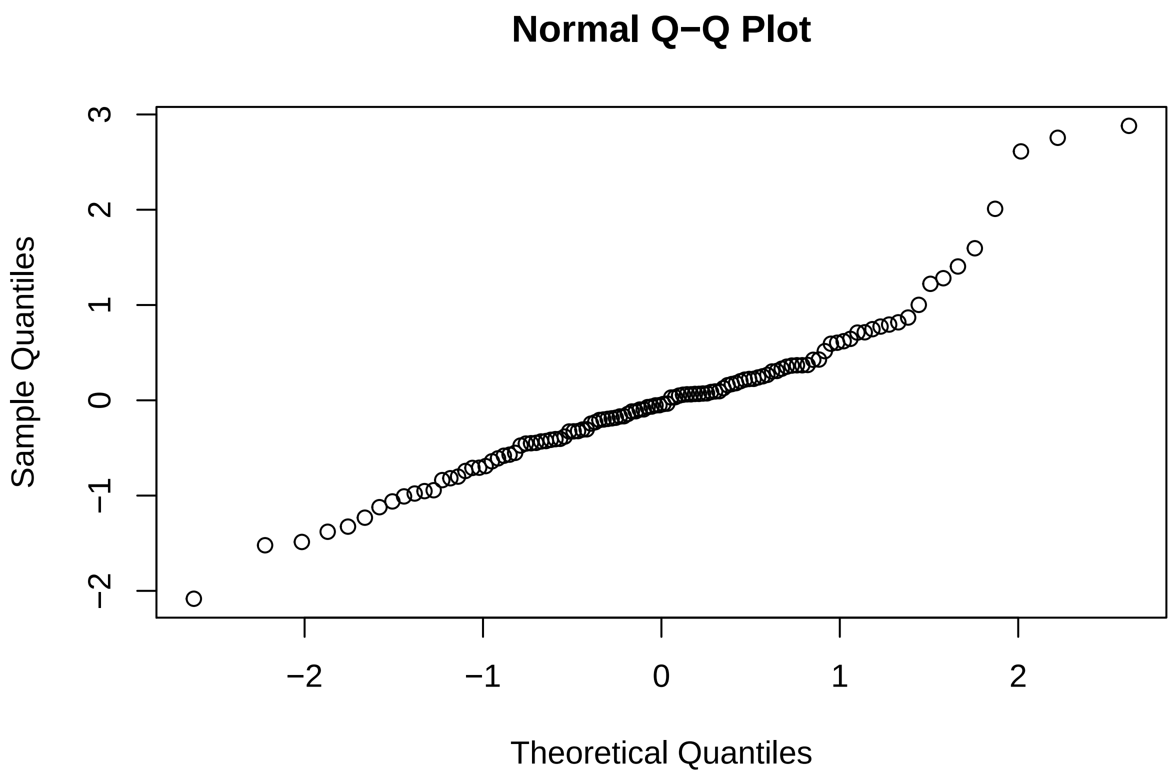


```r
summary(cont_reads_model)
```

```
## Linear mixed model fit by REML. t-tests use Satterthwaite's method [
## lmerModLmerTest]
## Formula: logit_freq ~ log_reads + (1 | taxon/sample)
##    Data: incorrect_df
##
## REML criterion at convergence: 387.7
##
## Scaled residuals:
##      Min       1Q   Median       3Q      Max
## -2.12730 -0.43988 -0.04604  0.33284  2.94219
##
## Random effects:
##  Groups       Name        Variance Std.Dev.
##  sample:taxon (Intercept) 0.8349   0.9137
##  taxon        (Intercept) 1.0386   1.0191
##  Residual                 0.9585   0.9790
## Number of obs: 114, groups:  sample:taxon, 57; taxon, 5
##
## Fixed effects:
##             Estimate Std. Error       df t value Pr(>|t|)
## (Intercept) -2.82888    1.30746 62.86978  -2.164   0.0343 *
## log_reads   -0.25364    0.09821 66.65948  -2.583   0.0120 *
## ---
## Signif. codes:  0 '***' 0.001 '**' 0.01 '*' 0.05 '.' 0.1 ' ' 1
##
## Correlation of Fixed Effects:
##           (Intr)
## log_reads -0.929
```

Now we will plot the proportion of incorrect matches against number of reads per library. We will connect libraries from the same sample

```
line_seq = seq(2, 7, 0.01)
line_df = data.frame(log_reads = line_seq, WGA = rep(c(TRUE,
    FALSE), each = length(line_seq)))
predicted = predict(cont_reads_model, line_df, re.form = NA)
transformed_predicted = exp(predicted)/(1 + exp(predicted))  #back-logit
prediction = cbind(line_df, x = 10^line_seq, y = transformed_predicted)

segments = data.frame(x1 = incorrect_df$Nreads[incorrect_df$WGA ==
    "FALSE"]/1000, y1 = incorrect_df$frequency[incorrect_df$WGA ==
    "FALSE"], x2 = incorrect_df$Nreads[incorrect_df$WGA == "TRUE"]/1000,
    y2 = incorrect_df$frequency[incorrect_df$WGA == "TRUE"])


p4 = ggplot(incorrect_df) + geom_point(aes(x = Nreads/1000, y = frequency,
    colour = WGA)) + scale_x_log10(limits = range(incorrect_df$Nreads/1000)) +
    scale_y_continuous(limits = c(0, 0.26), breaks = scales::pretty_breaks(n = 8)) +
    theme_tufte(base_size = 11.1) + theme(panel.border = element_rect(colour = "black",
    fill = NA)) + scale_colour_manual(values = brewer.pal(n = 3,
    name = "RdYlBu")[c(3, 1)], name = "", labels = c(`FALSE` = "gDNA",
    `TRUE` = "MDA")) + ylab("Proportion of incorrect reads") +
    xlab("Reads passing filter (thousands)") + geom_segment(data = segments,
    mapping = aes(x = x1, y = y1, xend = x2, yend = y2), size = 0.1,
    alpha = 0.7, arrow = arrow(angle = 30, length = unit(0.05,
        "inches"), ends = "last", type = "closed"))

print(p4)
```

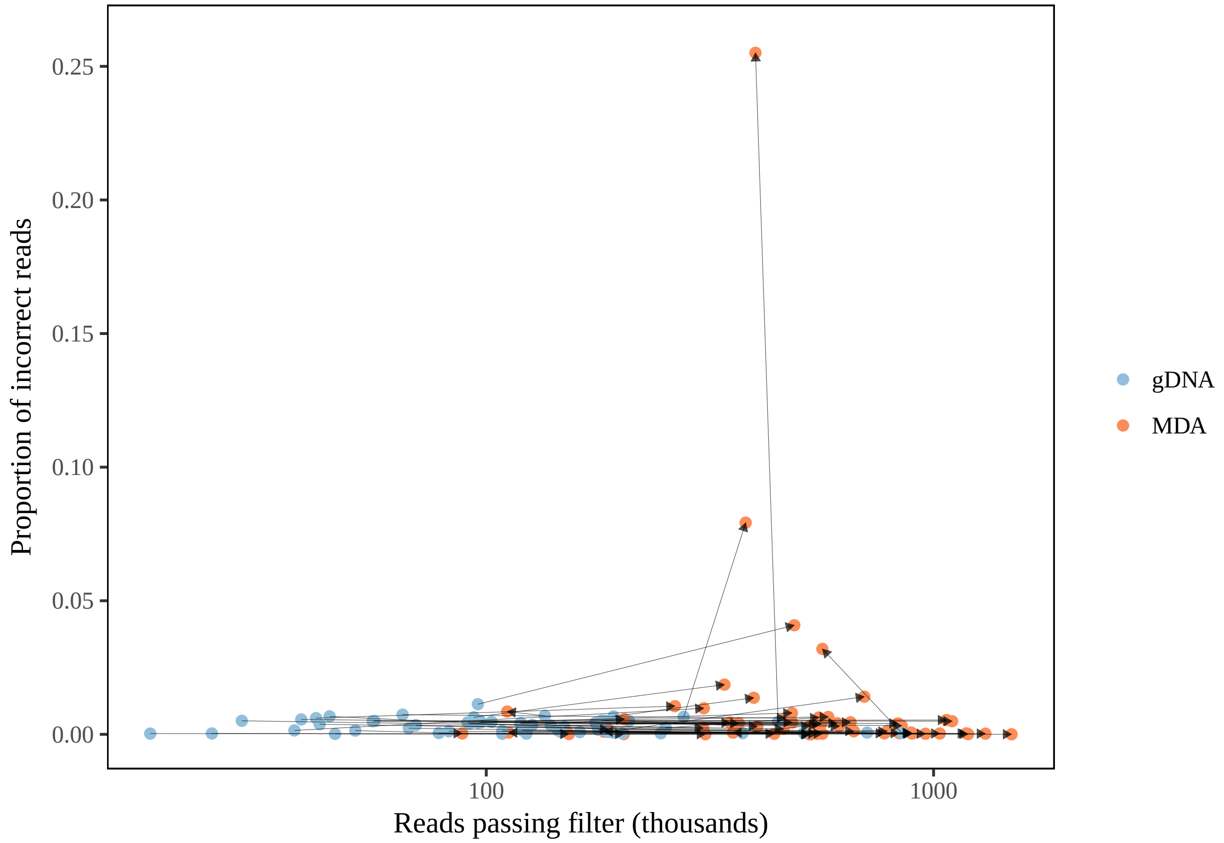

Nontemplated amplification in loci assembled for each sample

Do we see the same effect with loci assembled for each sample (i. e. those that passed the filter of minimum coverage of 7)?

```
cont_clusters_df = data.frame(sample = NULL, ipyrad_sample = NULL,
    taxon = NULL, match = NULL, frequency = NULL, pool = NULL)

for (sample in unique(row.names(cont_clusters))) {
    if (sample %in% sample_info_dup$samplename_ipyrad) {
        freqs = rep(NA, 4)
        names(freqs) = c("correct", "incorrect", "Homo", "none")
        taxon = as.character(sample_info_dup$taxon[sample_info_dup$samplename_ipyrad ==
            sample])
        freqs["correct"] = cont_clusters[sample, taxon]
        freqs["incorrect"] = sum(cont_clusters[sample, !grepl(paste(c(as.character(taxon),
            "Homo", "none"), collapse = "|"), colnames(cont_clusters))])
        freqs["Homo"] = cont_clusters[sample, "Homo"]
        freqs["none"] = cont_clusters[sample, "none"]
        cont_clusters_df = rbind(cont_clusters_df, data.frame(sample = strsplit(sample,
            "pool")[[1]][1], ipyrad_sample = sample, taxon = taxon,
            match = names(freqs), frequency = freqs, pool = strsplit(sample,
                "pool")[[1]][2]))
    }
}
row.names(cont_clusters_df) = NULL
```

```r
cont_clusters_df = merge(cont_clusters_df, sample_info_dup[c("samplename_ipyrad",
    "clusters_hidepth", "WGA")], by.x = "ipyrad_sample", by.y = "samplename_ipyrad")

cont_clusters_df$match = factor(as.character(cont_clusters_df$match),
    ordered = T, levels = c("correct", "incorrect", "Homo", "none"))


cont_clusters_df$Nloci = sapply(cont_clusters_df$ipyrad_sample,
    function(x) {
        sample_info$clusters_hidepth[sample_info$samplename_ipyrad ==
            as.character(x)]
    })

cont_clusters_df$Nreads = sapply(cont_clusters_df$ipyrad_sample,
    function(x) {
        sample_info$reads_passed_filter[sample_info$samplename_ipyrad ==
            as.character(x)]
    })

# this will make sure samples in the graph dataset are
# ordered by taxon
cont_clusters_df$sample = factor(as.character(cont_clusters_df$sample),
    ordered = T, levels = unique(cont_clusters_df$sample[order(as.character(cont_clusters_df$taxon))]))

p2 = ggplot(cont_clusters_df) + geom_bar(aes(x = sample, weight = frequency,
    fill = match)) + facet_grid(WGA ~ ., labeller = labeller(WGA = make_WGA_labels)) +
    theme_tufte() + scale_fill_few(palette = "medium", name = "",
    labels = setNames(c("Correct", "Incorrect", "Human", "No match"),
        c("correct", "incorrect", "Homo", "none"))) + theme(axis.text.x = element_blank(),
    axis.ticks.x = element_blank()) + xlab("Samples") + ylab("Loci assembled for each library")

print(p2)
```

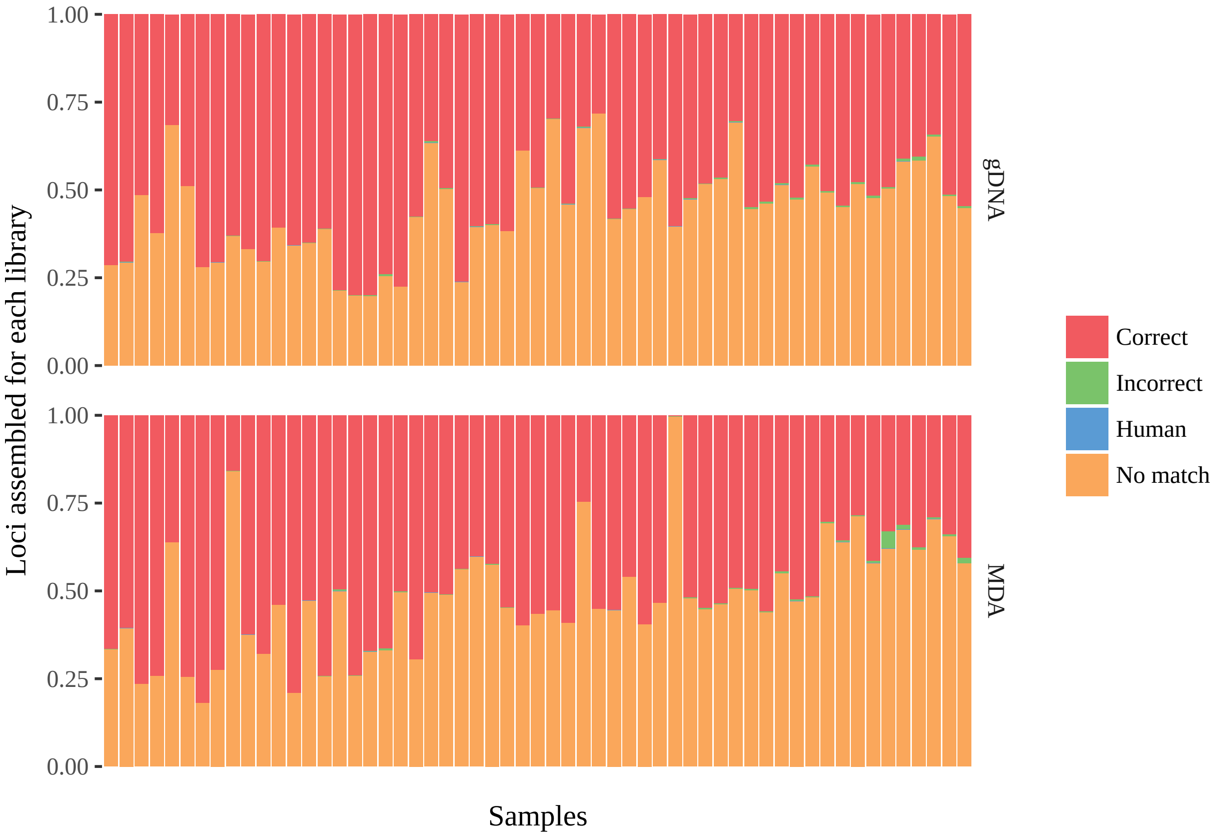

Just like for reads, there are a few samples with increase in incorrect, but overall the increase does not seem large.

```
incorrect_df = cont_clusters_df[cont_clusters_df$match == "incorrect",
    ]
incorrect_df$frequency = cont_clusters_df$frequency[cont_reads_df$match ==
    "incorrect"] + cont_reads_df$frequency[cont_clusters_df$match ==
    "Homo"]

incorrect_df$sample = factor(incorrect_df$sample, ordered = F)
incorrect_df$WGA = factor(incorrect_df$WGA, levels = c("FALSE",
    "TRUE"))  #this is just to nicely order the output of the model summary
incorrect_df$logit_freq = log((incorrect_df$frequency + 1e-04)/(1 -
    incorrect_df$frequency + 1e-04))
incorrect_df$log_Nloci = log(incorrect_df$Nloci)

cont_clusters_model_full = lmer(logit_freq ~ WGA * log_Nloci +
    (1 | taxon/sample), data = incorrect_df, REML = TRUE)

step_out = step(cont_clusters_model_full, reduce.random = F)
step_out
```

```
## Backward reduced random-effect table:
##
##                     Eliminated npar  logLik    AIC    LRT Df Pr(>Chisq)
## <none>                            7 -156.04 326.09
## (1 | sample:taxon)          0     6 -156.64 325.28  1.193  1     0.2748
## (1 | taxon)                 0     6 -182.78 377.56 53.471  1  2.624e-13 ***
```

```
## ---
## Signif. codes:  0 '***' 0.001 '**' 0.01 '*' 0.05 '.' 0.1 ' ' 1
##
## Backward reduced fixed-effect table:
## Degrees of freedom method: Satterthwaite
##
##                  Eliminated  Sum Sq Mean Sq NumDF   DenDF F value  Pr(>F)
## WGA:log_Nloci             1 2.07228 2.07228     1  95.073  3.1587 0.07872 .
## log_Nloci                 2 0.20607 0.20607     1 102.158  0.3278 0.56823
## WGA                       3 0.48859 0.48859     1  56.000  0.7757 0.38221
## ---
## Signif. codes:  0 '***' 0.001 '**' 0.01 '*' 0.05 '.' 0.1 ' ' 1
##
## Model found:
## logit_freq ~ (1 | taxon/sample)
```

```r
cont_clusters_model = get_model(step_out)

plot(cont_clusters_model)
```

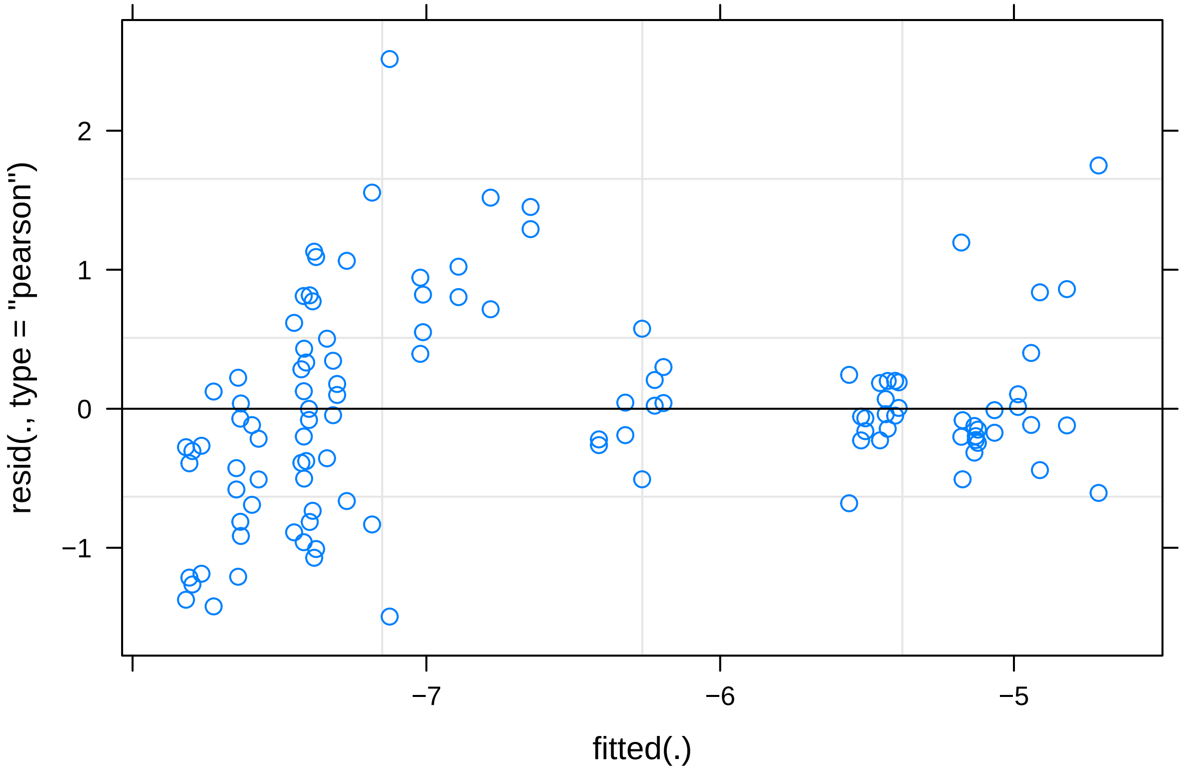


```r
qqnorm(resid(cont_clusters_model))
```

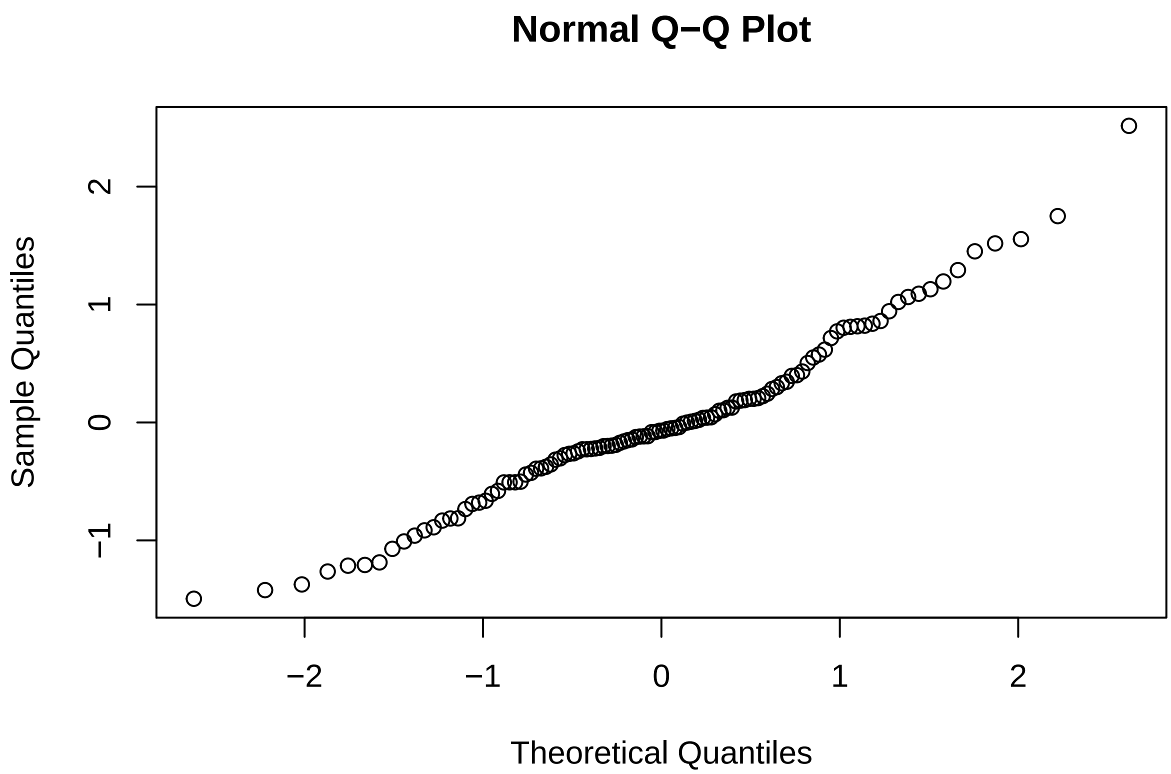


```r
summary(cont_clusters_model)
```

```
## Linear mixed model fit by REML. t-tests use Satterthwaite's method [
## lmerModLmerTest]
## Formula: logit_freq ~ (1 | taxon/sample)
##    Data: incorrect_df
##
## REML criterion at convergence: 309.8
##
## Scaled residuals:
##     Min      1Q  Median      3Q     Max
## -1.8875 -0.4872 -0.0882  0.4094  3.1758
##
## Random effects:
##  Groups       Name        Variance Std.Dev.
##  sample:taxon (Intercept) 0.1675   0.4092
##  taxon        (Intercept) 1.2354   1.1115
##  Residual                 0.6274   0.7921
## Number of obs: 114, groups:  sample:taxon, 57; taxon, 5
##
## Fixed effects:
##             Estimate Std. Error      df t value Pr(>|t|)
## (Intercept)  -6.3041     0.5074  4.0646  -12.43  0.00022 ***
## ---
## Signif. codes:  0 '***' 0.001 '**' 0.01 '*' 0.05 '.' 0.1 ' ' 1
```

```r
summary(incorrect_df$frequency)
```

```
##      Min.   1st Qu.    Median      Mean   3rd Qu.      Max.
## 1.880e-06 3.238e-04 1.612e-03 3.103e-03 4.528e-03 4.909e-02
```

Now let's look at the proportion incorrect only, against number of loci. Now the maximum proportion incorrect is much smaller:

```
segments = data.frame(x1 = incorrect_df$Nloci[incorrect_df$WGA ==
    "FALSE"], y1 = incorrect_df$frequency[incorrect_df$WGA ==
    "FALSE"], x2 = incorrect_df$Nloci[incorrect_df$WGA == "TRUE"],
    y2 = incorrect_df$frequency[incorrect_df$WGA == "TRUE"])



p5 = ggplot(incorrect_df) + geom_point(aes(x = Nloci, y = frequency,
    colour = WGA)) + scale_x_log10(limits = range(incorrect_df$Nloci)) +
    scale_y_continuous(limits = c(0, 0.26), breaks = scales::pretty_breaks(n = 8)) +
    theme_tufte(base_size = 11.1) + theme(panel.border = element_rect(colour = "black",
    fill = NA)) + scale_colour_manual(values = brewer.pal(n = 3,
    name = "RdYlBu")[c(3, 1)], name = "", labels = c(`FALSE` = "gDNA",
    `TRUE` = "MDA")) + ylab("Proportion of incorrect loci") +
    xlab("Loci assembled for each library") + geom_segment(data = segments,
    mapping = aes(x = x1, y = y1, xend = x2, yend = y2), size = 0.1,
    alpha = 0.7, arrow = arrow(angle = 30, length = unit(0.05,
        "inches"), ends = "last", type = "closed"))

print(p5)
```

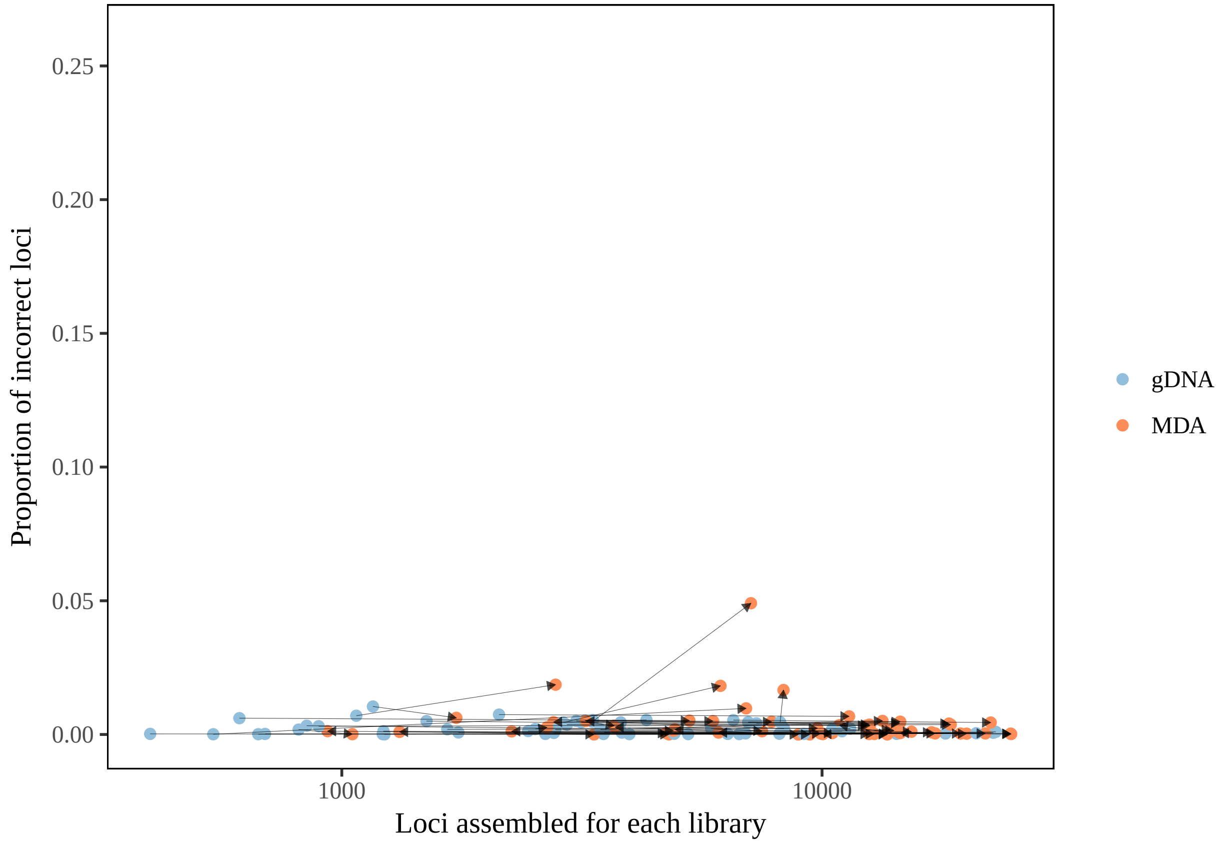

Nontemplated amplification in the final dataset

Repeating the same analyses, using loci in the final dataset:

```
cont_final_df = data.frame(sample = NULL, ipyrad_sample = NULL,
    taxon = NULL, match = NULL, frequency = NULL, pool = NULL)

for (sample in unique(row.names(cont_final))) {
    if (sample %in% sample_info_dup$samplename_ipyrad) {
        freqs = rep(NA, 4)
        names(freqs) = c("correct", "incorrect", "Homo", "none")
        taxon = as.character(sample_info_dup$taxon[sample_info_dup$samplename_ipyrad ==
            sample])
        freqs["correct"] = cont_final[sample, taxon]
        freqs["incorrect"] = sum(cont_final[sample, !grepl(paste(c(as.character(taxon),
            "Homo", "none"), collapse = "|"), colnames(cont_final))])
        freqs["Homo"] = cont_final[sample, "Homo"]
        freqs["none"] = cont_final[sample, "none"]
        cont_final_df = rbind(cont_final_df, data.frame(sample = strsplit(sample,
            "pool")[[1]][1], ipyrad_sample = sample, taxon = taxon,
            match = names(freqs), frequency = freqs, pool = strsplit(sample,
                "pool")[[1]][2]))
    }
}
row.names(cont_final_df) = NULL


cont_final_df = merge(cont_final_df, sample_info_dup[c("samplename_ipyrad",
    "N_loci_s7", "WGA", "clusters_hidepth", "Pfinal", "reads_passed_filter")],
    by.x = "ipyrad_sample", by.y = "samplename_ipyrad")

cont_final_df$match = factor(as.character(cont_final_df$match),
    ordered = T, levels = c("correct", "incorrect", "Homo", "none"))


# this will make sure samples in the graph dataset are
# ordered by taxon
cont_final_df$sample = factor(as.character(cont_final_df$sample),
    ordered = T, levels = unique(cont_final_df$sample[order(as.character(cont_final_df$taxon))]))

p3 = ggplot(cont_final_df) + geom_bar(aes(x = sample, weight = frequency,
    fill = match)) + facet_grid(WGA ~ ., labeller = labeller(WGA = make_WGA_labels)) +
    theme_tufte() + scale_fill_few(palette = "medium", name = "",
    labels = setNames(c("Correct", "Incorrect", "Human", "No match"),
        c("correct", "incorrect", "Homo", "none"))) + theme(axis.text.x = element_blank(),
    axis.ticks.x = element_blank()) + xlab("Samples") + ylab("Loci in final dataset")

print(p3)
```

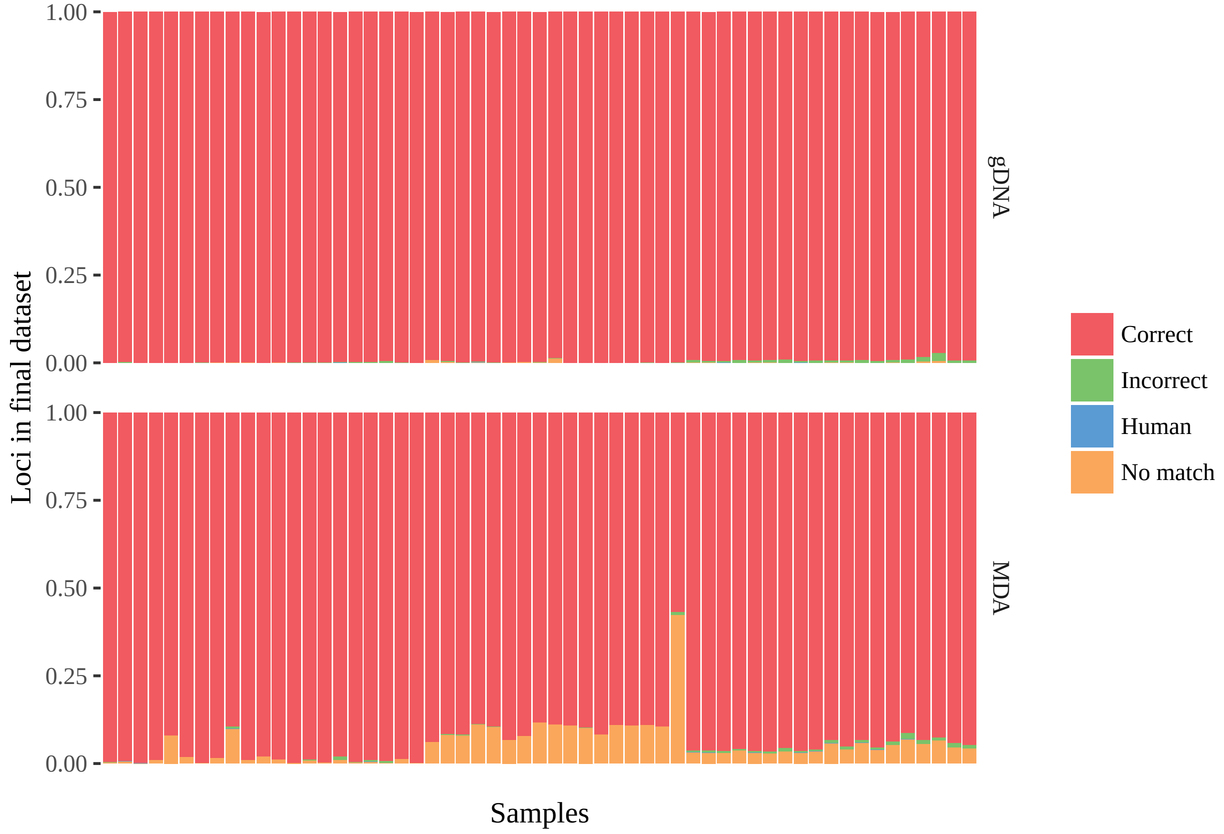

Visually, it is hard to tell whether MDA increases the amount of incorrect matches, which is very low overall. Most alignments are either to the correct species or have no match, in the case of MDA. This is expected, since only non-MDA samples were used for the reference dataset and we might expect some degree of non-overlap in the loci.

Is the effect significant?

```
incorrect_df = cont_final_df[cont_final_df$match == "incorrect",
    ]
incorrect_df$frequency = cont_final_df$frequency[cont_final_df$match ==
    "incorrect"] + cont_reads_df$frequency[cont_final_df$match ==
    "Homo"]

incorrect_df$sample = factor(incorrect_df$sample, ordered = F)
incorrect_df$WGA = factor(incorrect_df$WGA, levels = c("FALSE",
    "TRUE"))  #this is just to nicely order the output of the model summary
incorrect_df$logit_freq = log((incorrect_df$frequency + 1e-04)/(1 -
    incorrect_df$frequency + 1e-04))
incorrect_df$log_N_loci_s7 = log(incorrect_df$N_loci_s7)
incorrect_df$logit_Pfinal = logit(incorrect_df$Pfinal)

cont_final_model_full = lmer(logit_freq ~ WGA * log_N_loci_s7 +
    (1 | taxon/sample), data = incorrect_df, REML = TRUE)

step_out = step(cont_final_model_full, reduce.random = F)
step_out
```

```
## Backward reduced random-effect table:
```

```
##
##                       Eliminated npar  logLik    AIC    LRT Df Pr(>Chisq)
## <none>                              7 -157.68 329.36
## (1 | sample:taxon)          0     6 -162.35 336.70  9.342  1   0.002239 **
## (1 | taxon)                 0     6 -185.87 383.74 56.379  1  5.976e-14 ***
## ---
## Signif. codes:  0 '***' 0.001 '**' 0.01 '*' 0.05 '.' 0.1 ' ' 1
##
## Backward reduced fixed-effect table:
## Degrees of freedom method: Satterthwaite
##
##                      Eliminated Sum Sq Mean Sq NumDF  DenDF F value   Pr(>F)
## WGA:log_N_loci_s7             0 5.5761  5.5761     1 82.869  11.154 0.001258
##
## WGA:log_N_loci_s7 **
## ---
## Signif. codes:  0 '***' 0.001 '**' 0.01 '*' 0.05 '.' 0.1 ' ' 1
##
## Model found:
## logit_freq ~ WGA * log_N_loci_s7 + (1 | taxon/sample)
```

```
cont_final_model = get_model(step_out)

plot(cont_final_model)
```

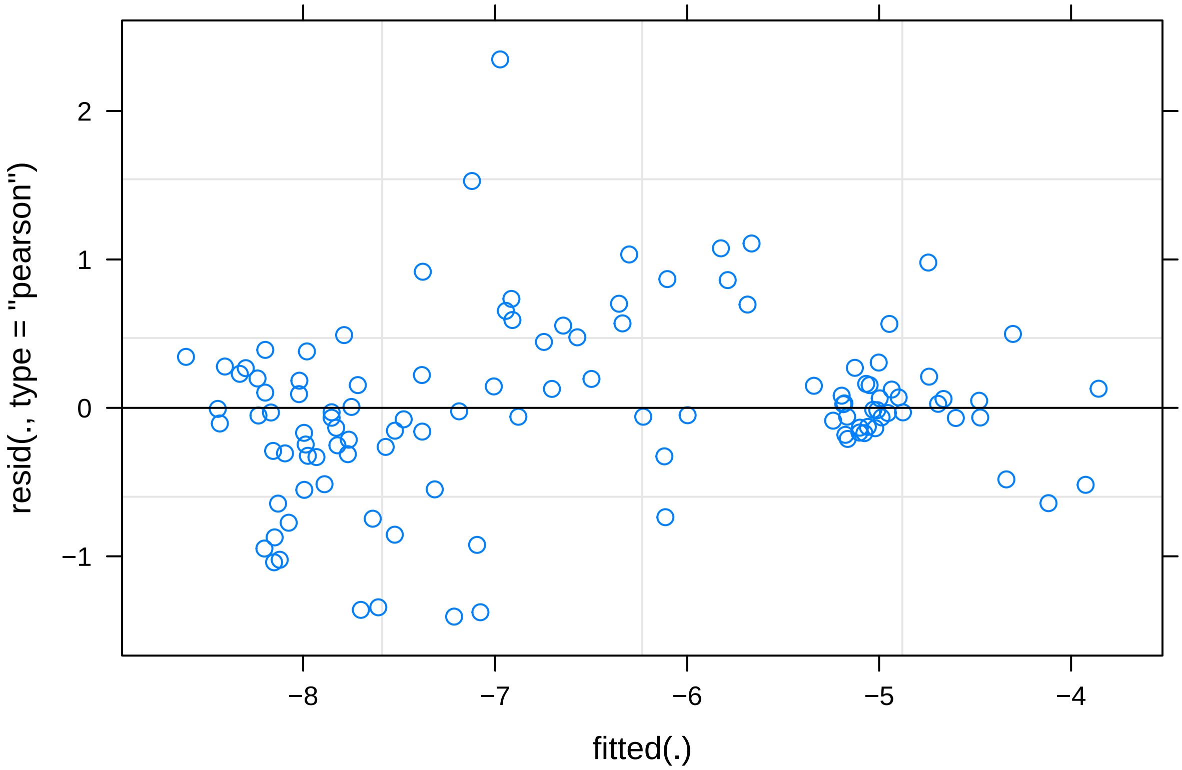


```
qqnorm(resid(cont_final_model))
```

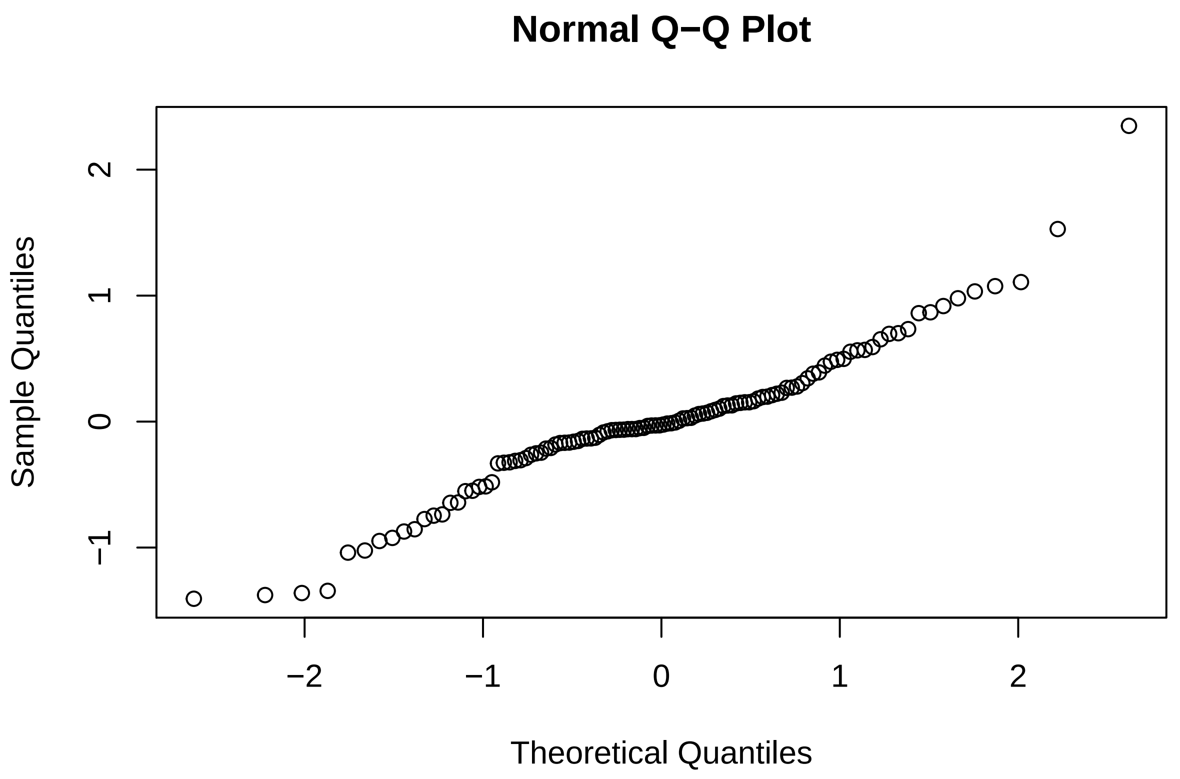


```r
summary(cont_final_model)
```

```
## Linear mixed model fit by REML. t-tests use Satterthwaite's method [
## lmerModLmerTest]
## Formula: logit_freq ~ WGA * log_N_loci_s7 + (1 | taxon/sample)
##    Data: incorrect_df
##
## REML criterion at convergence: 315.4
##
## Scaled residuals:
##     Min      1Q  Median      3Q     Max
## -1.9890 -0.3376 -0.0374  0.3215  3.3206
##
## Random effects:
##  Groups       Name        Variance Std.Dev.
##  sample:taxon (Intercept) 0.3540   0.595
##  taxon        (Intercept) 1.9164   1.384
##  Residual                 0.4999   0.707
## Number of obs: 114, groups:  sample:taxon, 57; taxon, 5
##
## Fixed effects:
##                         Estimate Std. Error        df t value Pr(>|t|)
## (Intercept)            -6.472758   0.913889 16.870006  -7.083 1.92e-06 ***
## WGATRUE                 3.986513   1.073476 82.647194   3.714  0.00037 ***
## log_N_loci_s7           0.003201   0.090613 83.627202   0.035  0.97190
## WGATRUE:log_N_loci_s7 -0.453328   0.135735 82.869287  -3.340  0.00126 **
## ---
## Signif. codes:  0 '***' 0.001 '**' 0.01 '*' 0.05 '.' 0.1 ' ' 1
##
## Correlation of Fixed Effects:
```

```
##                (Intr) WGATRUE l_N__7
## WGATRUE        -0.460
## log_N_lc_s7 -0.721  0.624
## WGATRUE:_N_   0.484 -0.991  -0.670
```

WGA and the interaction between WGA and number of loci are still significant in the final dataset, but the effect size is even smaller.

```
segments = data.frame(x1 = incorrect_df$N_loci_s7[incorrect_df$WGA ==
    "FALSE"], y1 = incorrect_df$frequency[incorrect_df$WGA ==
    "FALSE"], x2 = incorrect_df$N_loci_s7[incorrect_df$WGA ==
    "TRUE"], y2 = incorrect_df$frequency[incorrect_df$WGA ==
    "TRUE"])


p6 = ggplot(incorrect_df) + geom_point(aes(x = N_loci_s7, y = frequency,
    colour = WGA)) + scale_x_log10(limits = range(incorrect_df$N_loci_s7)) +
    scale_y_continuous(limits = c(0, 0.26), breaks = scales::pretty_breaks(n = 8)) +
    theme_tufte(base_size = 11.1) + theme(panel.border = element_rect(colour = "black",
    fill = NA)) + scale_colour_manual(values = brewer.pal(n = 3,
    name = "RdYlBu")[c(3, 1)], name = "", labels = c(`FALSE` = "gDNA",
    `TRUE` = "MDA")) + ylab("Proportion of incorrect loci in final dataset") +
    xlab("Loci in final dataset") + # geom_line(data = prediction, aes(x=x, y=y, colour = WGA)) +
geom_segment(data = segments, mapping = aes(x = x1, y = y1, xend = x2,
    yend = y2), size = 0.1, alpha = 0.7, arrow = arrow(angle = 30,
    length = unit(0.05, "inches"), ends = "last", type = "closed"))

print(p6)
```

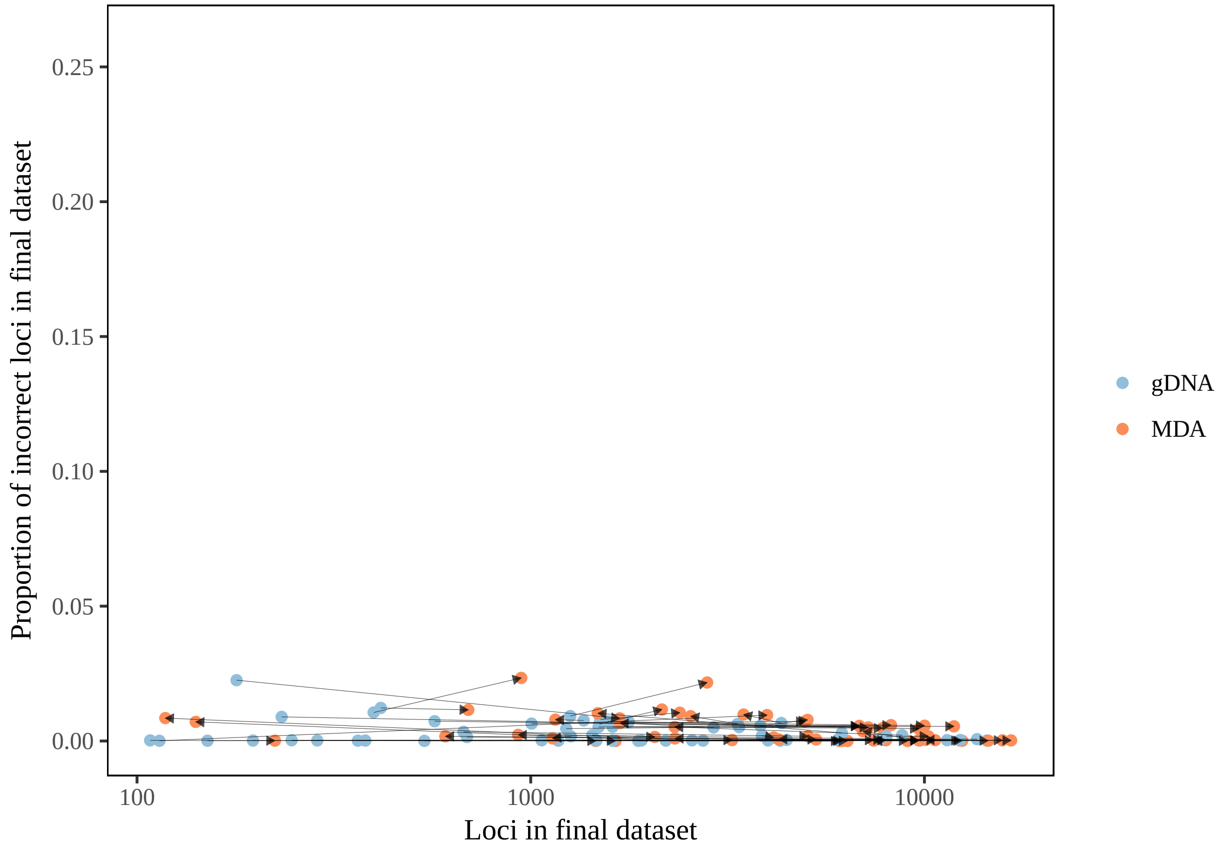

The average increase in proportion of incorrect matches is 0.001 and the maximum is 0.0128:

```
increases = tapply(X = incorrect_df$frequency, INDEX = incorrect_df$sample,
    FUN = function(x) {
        x[2] - x[1]
    })
increase_df = data.frame(sample = names(increases), increase = increases)

ggplot(increase_df) + geom_violin(aes(x = 1, y = increase)) +
    geom_jitter(aes(x = 1, y = increase)) + ylab(label = "Increase in the proportion of incorrect align
```

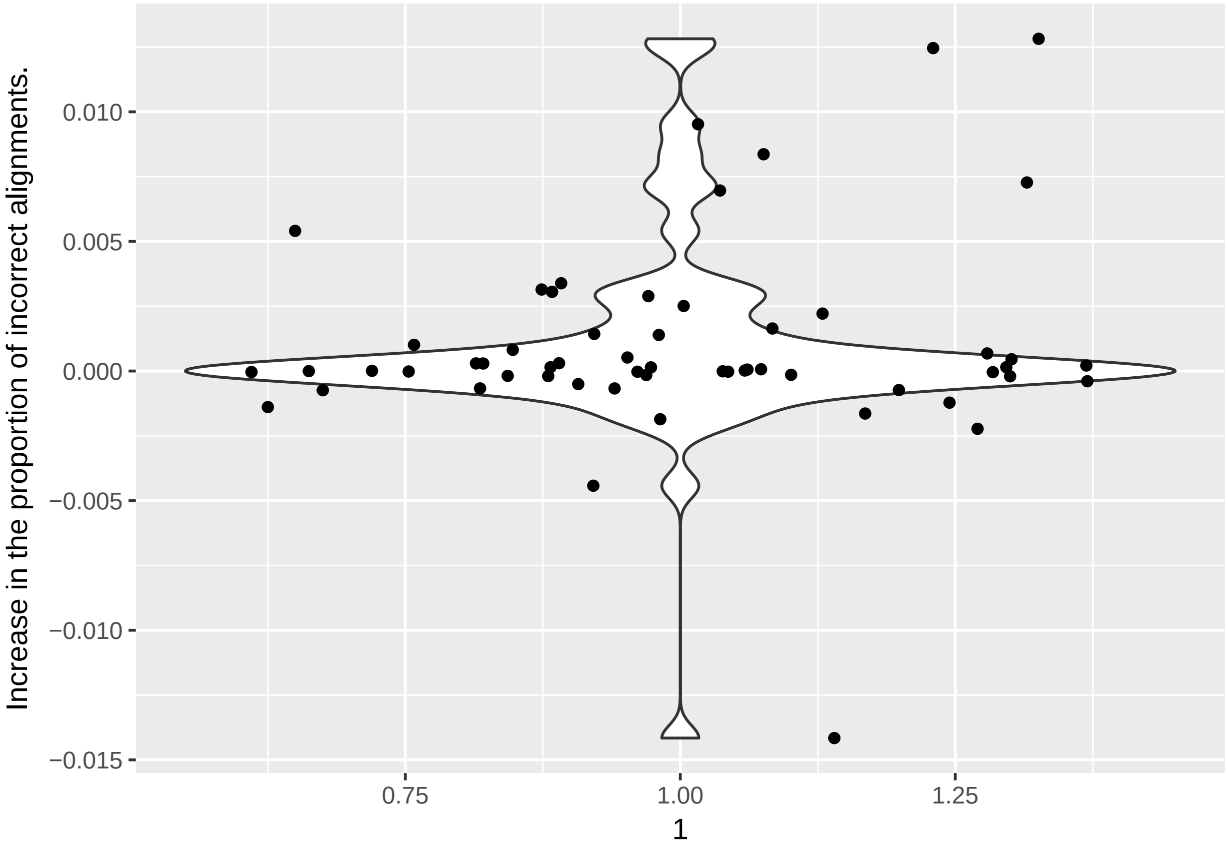


```
theme(axis.text.x = element_blank(), axis.title.x = element_blank(),
    axis.ticks.x = element_blank())
```

```
## List of 3
##  $ axis.title.x: list()
##   ..- attr(*, "class")= chr [1:2] "element_blank" "element"
##  $ axis.text.x : list()
##   ..- attr(*, "class")= chr [1:2] "element_blank" "element"
##  $ axis.ticks.x: list()
##   ..- attr(*, "class")= chr [1:2] "element_blank" "element"
##  - attr(*, "class")= chr [1:2] "theme" "gg"
##  - attr(*, "complete")= logi FALSE
##  - attr(*, "validate")= logi TRUE
```

```
mean(increases)
```

```
## [1] 0.001015994
```

```r
max(increases)
```

```
## [1] 0.01281569
```

Now plot everything.

```r
pdf(file = "plots/fig_contamination.pdf", width = 9 * 1.3, height = 6 *
    1.3, useDingbats = F)

hlay = rbind(c(1, 1, 1, 2, 2), c(3, 3, 3, 4, 4), c(5, 5, 5, 6,
    6))
grid.arrange(p1, p4, p2, p5, p3, p6, layout_matrix = hlay)

dev.off()
```

```
## pdf
##   2
```

```r
# This is to help put species names in Adobe Illustrator:
taxon_pos = data.frame(sample = levels(cont_clusters_df$sample),
    taxon = as.character(cont_clusters_df$taxon[match(levels(cont_clusters_df$sample),
        cont_clusters_df$sample)]))

taxon_pos$position = seq(1:dim(taxon_pos)[1])

tapply(taxon_pos$position, taxon_pos$taxon, range)
```

```
## $Anchylorhynchus
## [1]  1 21
##
## $Andranthobius
## [1] 22 26
##
## $Celetes_impar
## [1] 27 38
##
## $Microstrates_bondari
## [1] 39 47
##
## $Microstrates_ypsilon
## [1] 48 57
```

For publication, we edited thefigure above in Adobe Illustrator, adding taxon names and changing spacing between graphs. The output text above simply shows which bars in the graph correspond to which taxa, to help with image editing.

Comparing same samples with and without whole-genome amplification

It is still possible that there is contamination between closely related samples, which would not be eliminated using a minimum coverage by locus. One way to assess this type of contamination is to compare the genetic distance between a sample and itself. As a baseline, we will also compare each of these gDNA libraries to other gDNA libraries in the same taxon. Cases with likely significant contamination are those in which the distance between a gDNA library and its MDA library is larger than that between a gDNA library and its closest matching conspecific gDNA library.

We will plot the difference between these distances against the proportion of loci in the final dataset.

The figure below indicates that severe problems might all be among samples with lots of missing data. To avoid biases in the next few analyses, we further remove the MDA and gDNA libraries for the 9 samples with fewest loci in the final dataset for the MDA library

```
libraries = as.character(sample_info_dup$samplename_ipyrad[sample_info_dup$WGA ==
    F])
pairwise_distances = 1 - read.csv("pairwise_snps_N_matching_nuc.csv",
    row.names = 1)/2
pairwise_loci = read.csv("pairwise_snps_N_loci_total.csv", row.names = 1)

match_plot_df = data.frame(lib = NULL, taxon = NULL, population = NULL,
    taxpop = NULL, comparison = NULL, matches = NULL)

for (lib in libraries) {
    # first, get comparison to same sample with WGA
    sample = strsplit(lib, "pool")[[1]][1]
    taxon = as.character(unique(sample_info_dup$taxon[sample_info_dup$sample ==
        sample]))
    lib_wga = as.character(sample_info_dup$samplename_ipyrad[sample_info_dup$sample ==
        sample & sample_info_dup$WGA == T])


    # then to others in the same population
    criterion_dup = sample_info_dup$sample != as.character(sample) &
        sample_info_dup$taxon == as.character(taxon) & sample_info_dup$WGA ==
        FALSE
    pop_samples = as.character(sample_info_dup[criterion_dup,
        "samplename_ipyrad"])
    Npop = length(pop_samples)

    if (Npop) {
        indexer = matrix(data = c(rep(lib, Npop), pop_samples),
            nrow = Npop, byrow = F)
        indexer = indexer[pairwise_loci[indexer] > 35, ]

        if (is.null(dim(indexer))) {
            indexer = matrix(indexer, ncol = 2)
        }

        if (dim(indexer)[1] > 0) {
            match_plot_df = rbind(match_plot_df, data.frame(lib = lib,
                taxon = taxon, comparison = "wga", matches = pairwise_distances[lib,
                  lib_wga]))

            match_plot_df = rbind(match_plot_df, data.frame(lib = lib,
                taxon = taxon, comparison = "other", matches = pairwise_distances[indexer]))
        }

    }

}
# Now simplify dataframe to get the minimum distance only:
dist_df = as.data.frame.table(tapply(match_plot_df$matches, match_plot_df[c("lib",
    "comparison")], min), responseName = "distance")
```

```
dist_df = unique(merge(dist_df, match_plot_df[-length(match_plot_df)],
    all.y = FALSE))
dist_df$sample = sapply(dist_df$lib, function(x) {
    strsplit(as.character(x), split = "pool")[[1]][1]
})
dist_df = unique(merge(dist_df, sample_info_dup[sample_info_dup$WGA,
    c("sample", "N_loci_s7")], by.x = "sample", by.y = "sample"))


dist_diff_df = as.data.frame.table(tapply(dist_df$distance, dist_df[c("sample",
    "taxon")], function(x) {
    x[1] - x[2]
}), responseName = "diff_distance")
dist_diff_df = dist_diff_df[!is.na(dist_diff_df$diff_distance),
    ]
dist_diff_df = unique(merge(dist_diff_df, dist_df[c("sample",
    "N_loci_s7")]))

# this will make taxa be plotted in correct order in legend
dist_diff_df$taxon = factor(dist_diff_df$taxon, levels = c("Anchylorhynchus",
    "Andranthobius", "Celetes_impar", "Microstrates_bondari",
    "Microstrates_ypsilon"))



p = ggplot(dist_diff_df) + geom_hline(aes(yintercept = 0), alpha = 0.7) +
    geom_point(aes(x = N_loci_s7, y = diff_distance, colour = taxon)) +
    theme_tufte() + theme(panel.border = element_rect(colour = "black",
    fill = NA)) + scale_colour_brewer(type = "qualitative", palette = "Accent",
    name = "Taxon", labels = c(Anchylorhynchus = "Anchylorhynchus",
        Andranthobius = "Andranthobius", Celetes_impar = "C. impar",
        Microstrates_bondari = "M. bondari", Microstrates_ypsilon = "M. ypsilon")) +
    scale_x_log10() + xlab("Loci in final dataset for MDA library") +
    ylab("Difference in genetic distance\n(self MDA - other gDNA library)")

print(p)
```

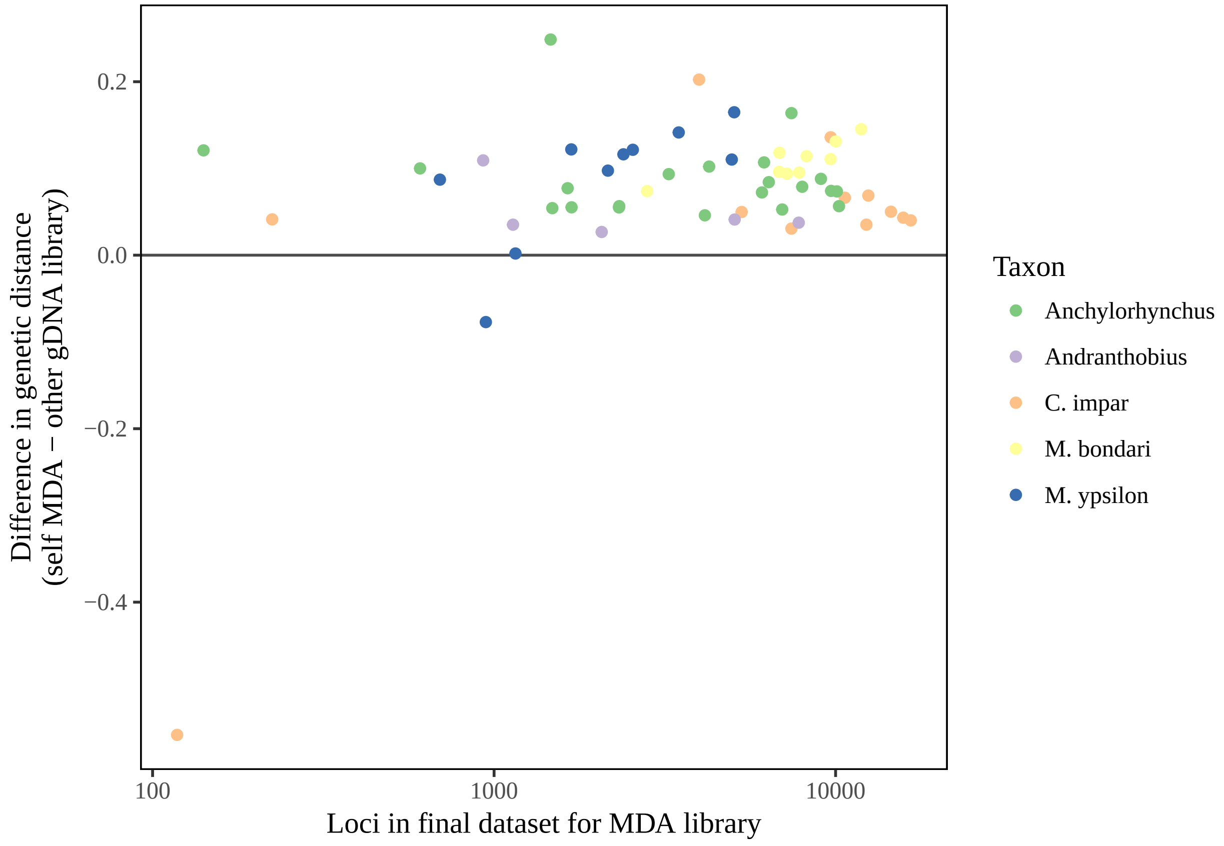


```
pdf(file = "plots/fig_close_contamination.pdf", width = 7, height = 3.5,
    useDingbats = F)
print(p)
dev.off()
```

```
## pdf
##   2
```

Now we will remove the 9 samples with fewest loci in the final dataset:

```
problems = as.character(dist_diff_df$sample[rank(as.integer(dist_diff_df$N_loci_s7)) <=
    9])
problems_libs = sample_info_dup$samplename_ipyrad[grepl(paste(problems,
    collapse = "|"), sample_info_dup$sample) & sample_info_dup$WGA ==
    TRUE]
sample_info_sing = rbind(sample_info_sing, sample_info_dup[(sample_info_dup$WGA ==
    F & grepl(paste(problems, collapse = "|"), sample_info_dup$sample)),
    ])

prun_sample_info_dup = sample_info_dup[!(grepl(paste(problems,
    collapse = "|"), sample_info_dup$sample)), ]

prun_sample_info_dup$WGA = factor(prun_sample_info_dup$WGA, ordered = F,
    levels = c("FALSE", "TRUE"))
```

Comparing MDA and gDNA for number of loci, heterozygosity and GC bias

Here we calculate some statistics for each sample and fit a linear model controlling for covariates. In all cases, taxon and sample nested within taxon are random effects, and WGA is a fixed effect. In the case of comparison of number of clusters, number of reads is a fixed effect, and in the others number of clusters is a fixed effetc. In this section, we only include samples that had libraries prepared both with and without whole genome amplification

Does whole-genome amplification reduce the number of loci obtained in comparison to gDNA libraries?

First, figure out the best model.

We had to some some scale transformations to fit a linear model: * reads were transformed using log10 * number of clusters was transformed using logit, assuming a maximum of 30000 clusters

Then, we use step() to find non-significant coefficients. Model output and disgnostic plots:

```
max_clusters = 30000  #sample with > 3 mi reads had ~30K clusters

log_reads = log10(prun_sample_info_dup$reads_passed_filter)
logit_clusters = log(prun_sample_info_dup$clusters_hidepth/max_clusters/(1 -
    prun_sample_info_dup$clusters_hidepth/max_clusters))  #logit = log(p/1-p)


reads_model_full = lmer(logit_clusters ~ log_reads + WGA + log_reads:WGA +
    (1 | taxon/sample), data = prun_sample_info_dup, REML = TRUE)


step_out = step(reads_model_full, reduce.random = FALSE)
step_out
```

```
## Backward reduced random-effect table:
##
##                     Eliminated npar  logLik    AIC    LRT Df Pr(>Chisq)
## <none>                           7 -78.603 171.21
## (1 | sample:taxon)           0    6 -78.603 169.21 0.0000  1     1.0000
## (1 | taxon)                  0    6 -79.594 171.19 1.9814  1     0.1592
##
## Backward reduced fixed-effect table:
## Degrees of freedom method: Satterthwaite
##
##               Eliminated Sum Sq Mean Sq NumDF  DenDF F value  Pr(>F)
## log_reads:WGA          0 1.6505  1.6505     1 91.729  5.9599 0.01655 *
## ---
## Signif. codes:  0 '***' 0.001 '**' 0.01 '*' 0.05 '.' 0.1 ' ' 1
##
## Model found:
## logit_clusters ~ log_reads + WGA + log_reads:WGA + (1 | taxon/sample)
```

```
reads_model = get_model(step_out)

summary(reads_model)
```

```
## Linear mixed model fit by REML. t-tests use Satterthwaite's method [
## lmerModLmerTest]
## Formula:
## logit_clusters ~ log_reads + WGA + log_reads:WGA + (1 | taxon/sample)
##    Data: prun_sample_info_dup
##
## REML criterion at convergence: 157.2
##
## Scaled residuals:
##     Min      1Q  Median      3Q     Max
## -4.0060 -0.7076  0.1602  0.5539  2.5186
##
## Random effects:
##  Groups       Name        Variance Std.Dev.
##  sample:taxon (Intercept) 0.00000  0.0000
##  taxon        (Intercept) 0.02744  0.1656
##  Residual                 0.27694  0.5262
## Number of obs: 96, groups:  sample:taxon, 48; taxon, 5
##
## Fixed effects:
##                   Estimate Std. Error       df t value Pr(>|t|)
## (Intercept)       -19.9703     1.1258  91.3423 -17.739   <2e-16 ***
## log_reads           3.5526     0.2217  90.9580  16.025   <2e-16 ***
## WGATRUE             4.7302     2.2700  91.6698   2.084   0.0400 *
## log_reads:WGATRUE  -0.9976     0.4086  91.7289  -2.441   0.0166 *
## ---
## Signif. codes:  0 '***' 0.001 '**' 0.01 '*' 0.05 '.' 0.1 ' ' 1
##
## Correlation of Fixed Effects:
##             (Intr) lg_rds WGATRU
## log_reads   -0.995
## WGATRUE     -0.522  0.524
## lg_:WGATRUE  0.566 -0.570 -0.997
```

```
plot(reads_model)
```

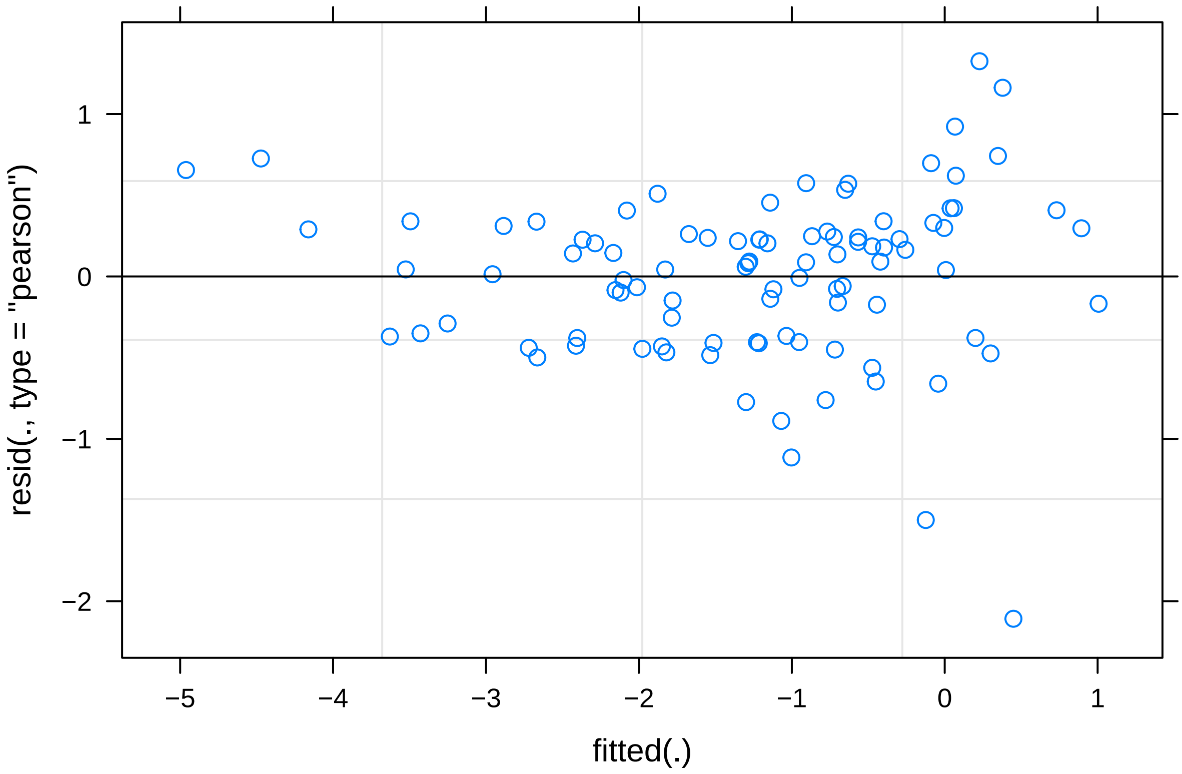


```r
qqnorm(resid(reads_model), main = "Q-Q plot for residuals")
```

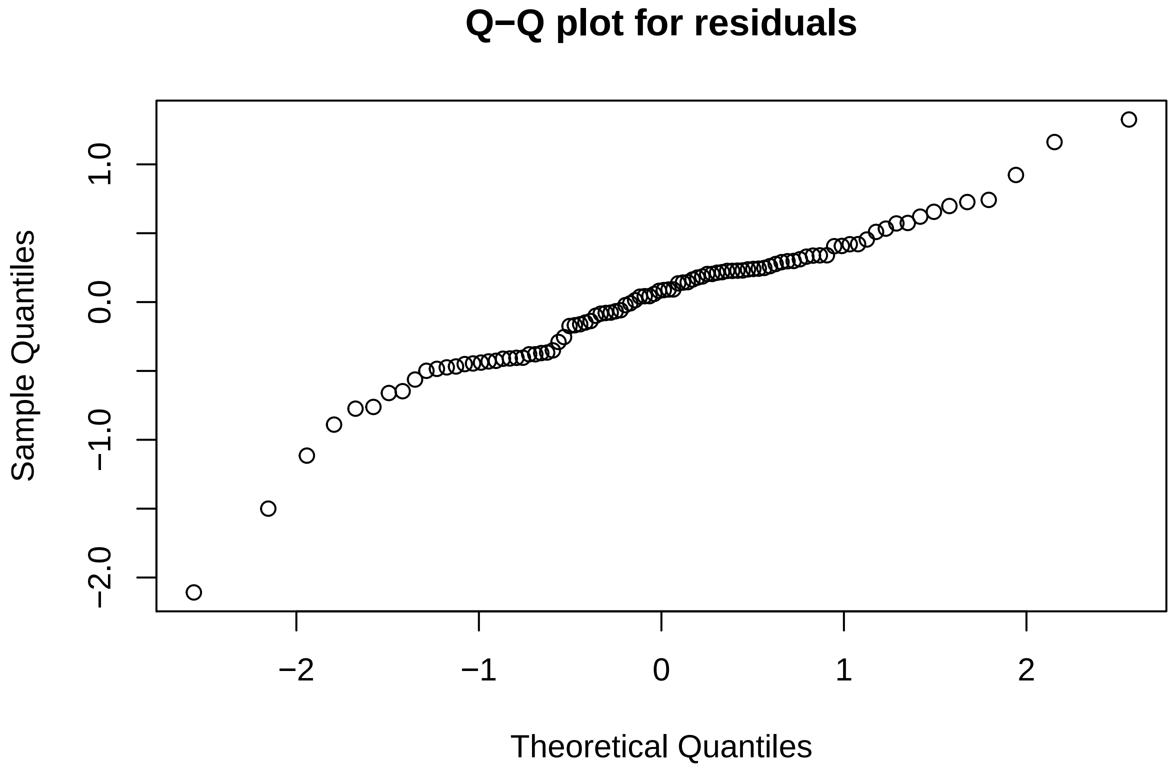

Yes, it seems that whole-genome amplification decreases the number of clusters obtained for a sample. By how much?

```
line_seq = seq(2, 7, 0.01)
line_df = data.frame(log_reads = line_seq, WGA = rep(c("TRUE",
    "FALSE"), each = length(line_seq)))
predicted = predict(reads_model, line_df, re.form = NA)
transformed_predicted = exp(predicted)/(1 + exp(predicted)) *
    max_clusters  #back-logit
# transformed_predicted = exp(predicted)
prediction = cbind(line_df, x = 10^line_seq, y = transformed_predicted)

range(prediction$y[prediction$WGA == TRUE & prediction$x > 2e+05 &
    prediction$x < 1500000]/prediction$y[prediction$WGA == FALSE &
    prediction$x > 2e+05 & prediction$x < 1500000])
```

```
## [1] 0.5872886 0.7176143
```

WGA results in a reduction of about 58%-72% in the number of clusters for a range of sensible number of reads (200K to 1.5M).

Now, plotting data and model predictions:

```
p = ggplot() + geom_point(data = prun_sample_info_dup, mapping = aes(x = reads_passed_filter/1000,
    y = clusters_hidepth/1000, colour = WGA)) + scale_x_continuous(limits = c(0,
    max(prun_sample_info_dup$reads_passed_filter)/1000 + 100)) +
    scale_y_continuous(limits = c(0, max(prun_sample_info_dup$clusters_hidepth/1000) +
        0.1)) + geom_line(data = prediction, mapping = aes(x = x/1000,
    y = y/1000, colour = WGA)) + theme_tufte() + theme(panel.border = element_rect(colour = "black",
    fill = NA)) + scale_colour_manual(values = brewer.pal(n = 3,
    name = "RdYlBu")[c(3, 1)], name = "", labels = c(`FALSE` = "gDNA",
    `TRUE` = "MDA")) + ylab("Assembled loci (thousands)") + xlab("Reads passing filter (thousands)")

print(p)
```

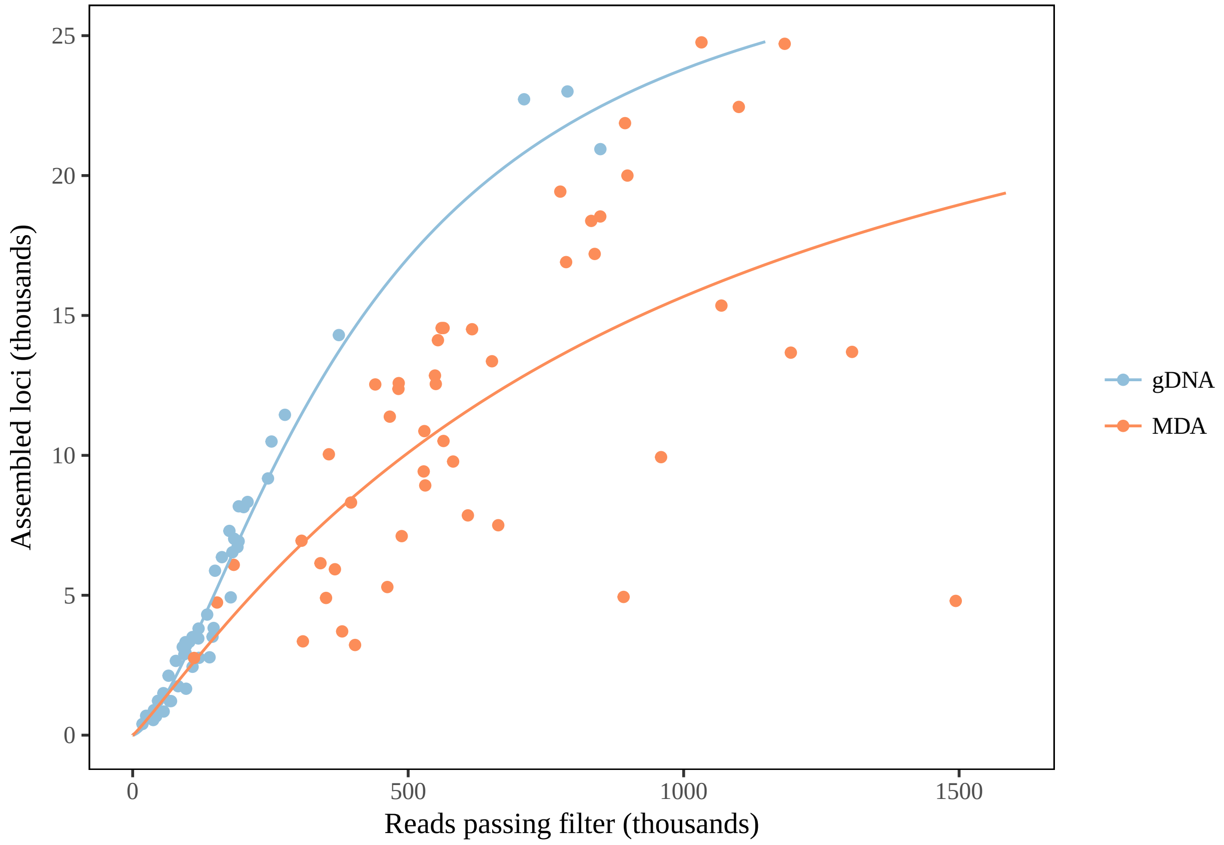


```
pdf("plots/fig_nloci.pdf", width = 7, height = 3.5, useDingbats = F)
print(p)
dev.off()
```

```
## pdf
##   2
```

What is the effect of MDA on variance of read depth among loci?

```
log_reads = log10(prun_sample_info_dup$reads_passed_filter)
sd_coverage = log(prun_sample_info_dup$sd_depth_total)

cov_model_full = lmer(sd_coverage ~ log_reads + WGA + log_reads:WGA +
    (1 | taxon/sample), data = prun_sample_info_dup, REML = TRUE)


step_out = step(cov_model_full, reduce.random = FALSE)
step_out
```

```
## Backward reduced random-effect table:
##
##                      Eliminated npar   logLik    AIC    LRT Df Pr(>Chisq)
## <none>                             7  -83.256 180.51
## (1 | sample:taxon)             0    6  -83.256 178.51  0.000  1          1
## (1 | taxon)                    0    6 -104.733 221.47 42.953  1  5.608e-11
##
```

```
## <none>
## (1 | sample:taxon)
## (1 | taxon)        ***
## ---
## Signif. codes:  0 '***' 0.001 '**' 0.01 '*' 0.05 '.' 0.1 ' ' 1
##
## Backward reduced fixed-effect table:
## Degrees of freedom method: Satterthwaite
##
##               Eliminated  Sum Sq Mean Sq NumDF  DenDF F value    Pr(>F)
## log_reads:WGA          1  0.9025  0.9025     1 88.217  3.2696   0.07398
## log_reads              0 11.6294 11.6294     1 88.893 41.0420 6.908e-09
## WGA                    0  8.2161  8.2161     1 88.847 28.9959 5.865e-07
##
## log_reads:WGA .
## log_reads     ***
## WGA           ***
## ---
## Signif. codes:  0 '***' 0.001 '**' 0.01 '*' 0.05 '.' 0.1 ' ' 1
##
## Model found:
## sd_coverage ~ log_reads + WGA + (1 | taxon/sample)
```

```
cov_model = get_model(step_out)

summary(cov_model)
```

```
## Linear mixed model fit by REML. t-tests use Satterthwaite's method [
## lmerModLmerTest]
## Formula: sd_coverage ~ log_reads + WGA + (1 | taxon/sample)
##    Data: prun_sample_info_dup
##
## REML criterion at convergence: 169.8
##
## Scaled residuals:
##     Min      1Q  Median      3Q     Max
## -2.6504 -0.6904 -0.2750  0.5646  2.8927
##
## Random effects:
##  Groups       Name        Variance Std.Dev.
##  sample:taxon (Intercept) 0.0000   0.0000
##  taxon        (Intercept) 0.4853   0.6966
##  Residual                 0.2834   0.5323
## Number of obs: 96, groups:  sample:taxon, 48; taxon, 5
##
## Fixed effects:
##             Estimate Std. Error      df t value Pr(>|t|)
## (Intercept)  -2.7245     0.9884 84.8948  -2.757  0.00715 **
## log_reads     1.1844     0.1849 88.8934   6.406 6.91e-09 ***
## WGATRUE       0.8934     0.1659 88.8467   5.385 5.87e-07 ***
## ---
## Signif. codes:  0 '***' 0.001 '**' 0.01 '*' 0.05 '.' 0.1 ' ' 1
##
## Correlation of Fixed Effects:
##           (Intr) lg_rds
```

```
## log_reads -0.945
## WGATRUE    0.678 -0.756
```

```r
plot(cov_model)
```

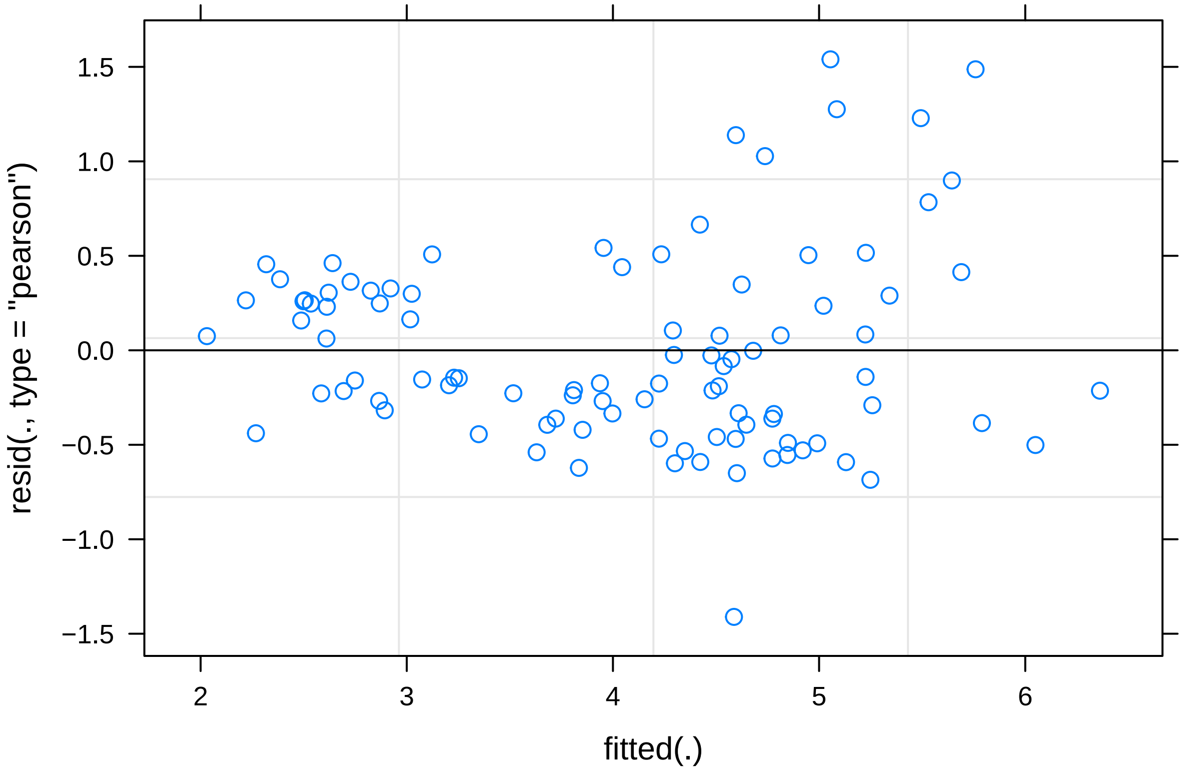


```r
qqnorm(resid(cov_model), main = "Q-Q plot for residuals")
```

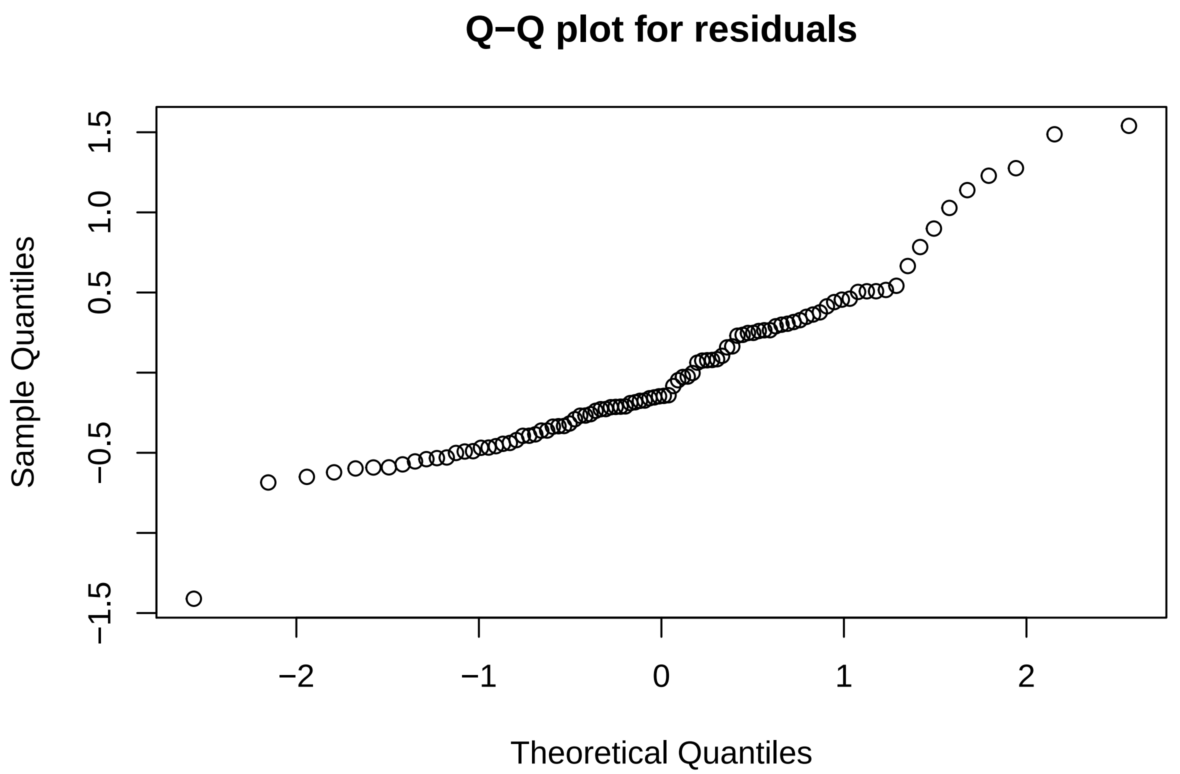

The standard deviation in read depth is affected by MDA, let's plot:

```
line_seq = seq(2, 7, 0.01)
line_df = data.frame(log_reads = line_seq, WGA = rep(c("TRUE",
    "FALSE"), each = length(line_seq)))
predicted = predict(cov_model, line_df, re.form = NA)
transformed_predicted = exp(predicted)
prediction = cbind(line_df, x = 10^line_seq, y = transformed_predicted)

p = ggplot() + geom_point(data = prun_sample_info_dup, mapping = aes(x = reads_passed_filter/1000,
    y = prun_sample_info_dup$sd_depth_total, colour = WGA)) +
    scale_x_continuous(limits = c(0, max(prun_sample_info_dup$reads_passed_filter)/1000 +
        100)) + scale_y_continuous(limits = c(0, max(prun_sample_info_dup$sd_depth_total) +
    100)) + geom_line(data = prediction, mapping = aes(x = x/1000,
    y = y, colour = WGA)) + # geom_segment(data = segments, mapping = aes(x = x1, y = y1,
# xend = x2, yend = y2), size = 0.1, alpha = 0.7,
# arrow=arrow(angle = 30, length = unit(0.05, 'inches'),ends
# = 'last', type = 'closed')) +
theme_tufte() + theme(panel.border = element_rect(colour = "black",
    fill = NA)) + scale_colour_manual(values = brewer.pal(n = 3,
    name = "RdYlBu")[c(3, 1)], name = "", labels = c(`FALSE` = "gDNA",
    `TRUE` = "MDA")) + ylab("Standard deviation of coverage in assembled loci") +
    xlab("Reads passing filter (thousands)")

print(p)
```

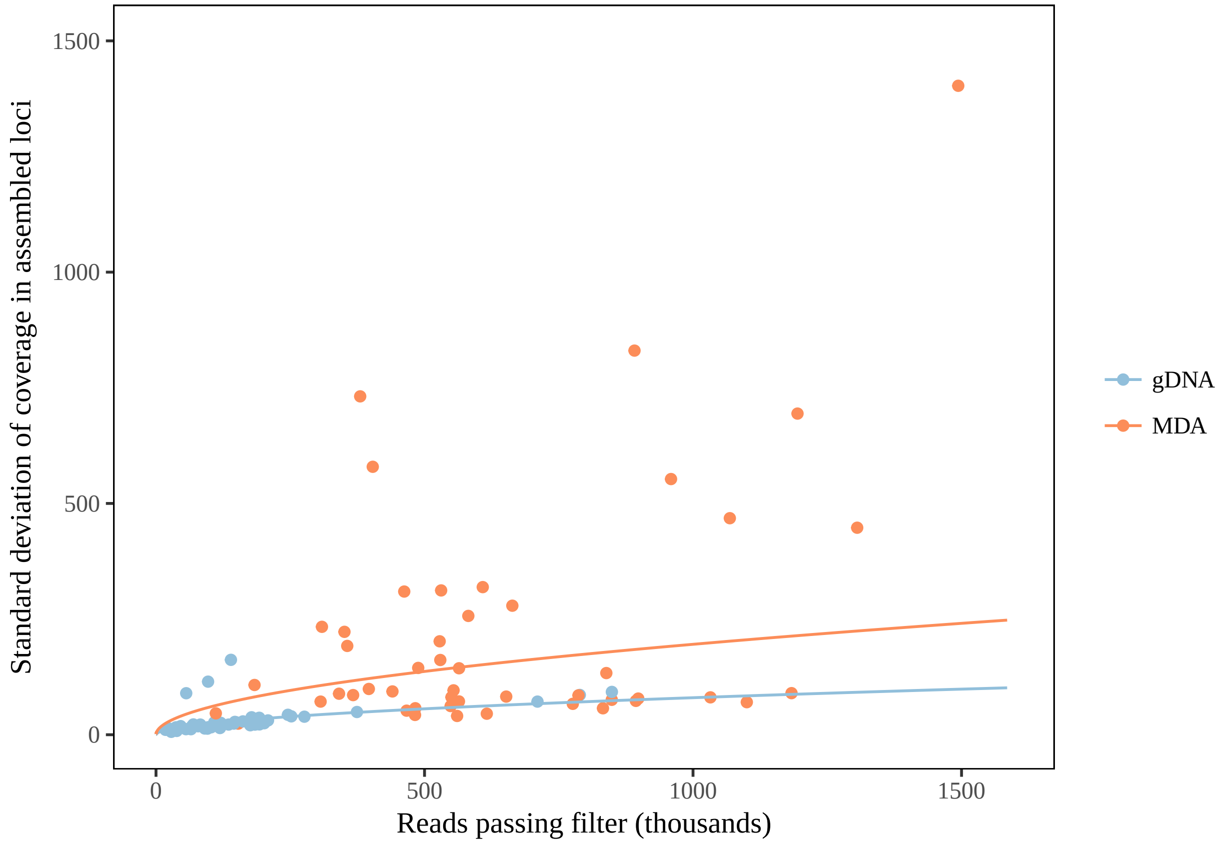


```
pdf("plots/fig_sdcov.pdf", width = 7, height = 3.5, useDingbats = F)
print(p)
dev.off()
```

```
## pdf
##   2
```

Does MDA interfere with heterozygosity?

Here we will make two models. One in which number of loci is in the predictors and other in which average coverage replaces it. A model including both at the same time and interactions was too hard to interpret. We will also plot the relationship between average coverage and number of loci.

Model fitting:

```
Nclusters = log(prun_sample_info_dup$clusters_hidepth)
het = prun_sample_info_dup$N_nucleotides_heteroz/prun_sample_info_dup$N_nucleotides_s7
avg_depth = log(prun_sample_info_dup$avg_depth_stat)

het_avg_model_full = lmer(het ~ WGA * avg_depth + (1 | taxon/sample),
    data = prun_sample_info_dup, REML = TRUE)
step_out = step(het_avg_model_full, reduce.random = FALSE)
step_out
```

```
## Backward reduced random-effect table:
##
##                      Eliminated npar logLik     AIC     LRT Df Pr(>Chisq)
## <none>                             7 486.36 -958.72
```

```
## (1 | sample:taxon)          0    6 485.02 -958.04  2.6783  1   0.101724
## (1 | taxon)                 0    6 480.88 -949.76 10.9600  1   0.000931
##
## <none>
## (1 | sample:taxon)
## (1 | taxon)        ***
## ---
## Signif. codes:  0 '***' 0.001 '**' 0.01 '*' 0.05 '.' 0.1 ' ' 1
##
## Backward reduced fixed-effect table:
## Degrees of freedom method: Satterthwaite
##
##               Eliminated    Sum Sq   Mean Sq NumDF  DenDF F value  Pr(>F)
## WGA:avg_depth          0 6.372e-06 6.372e-06     1 85.036  6.6537 0.01161
##
## WGA:avg_depth *
## ---
## Signif. codes:  0 '***' 0.001 '**' 0.01 '*' 0.05 '.' 0.1 ' ' 1
##
## Model found:
## het ~ WGA * avg_depth + (1 | taxon/sample)
```

```
het_avg_model = get_model(step_out)
summary(het_avg_model)
```

```
## Linear mixed model fit by REML. t-tests use Satterthwaite's method [
## lmerModLmerTest]
## Formula: het ~ WGA * avg_depth + (1 | taxon/sample)
##    Data: prun_sample_info_dup
##
## REML criterion at convergence: -972.7
##
## Scaled residuals:
##      Min       1Q   Median       3Q      Max
## -2.28945 -0.44652 -0.02966  0.60728  1.67387
##
## Random effects:
##  Groups       Name        Variance  Std.Dev.
##  sample:taxon (Intercept) 3.125e-07 0.0005590
##  taxon        (Intercept) 4.875e-07 0.0006982
##  Residual                 9.577e-07 0.0009786
## Number of obs: 96, groups:  sample:taxon, 48; taxon, 5
##
## Fixed effects:
##                      Estimate Std. Error         df t value Pr(>|t|)
## (Intercept)         0.0005733  0.0023253 70.6290541   0.247  0.80599
## WGATRUE             0.0079942  0.0026461 87.1166767   3.021  0.00331 **
## avg_depth           0.0010938  0.0007806 73.0167969   1.401  0.16538
## WGATRUE:avg_depth -0.0022127  0.0008578 85.0361456  -2.579  0.01161 *
## ---
## Signif. codes:  0 '***' 0.001 '**' 0.01 '*' 0.05 '.' 0.1 ' ' 1
##
## Correlation of Fixed Effects:
##             (Intr) WGATRUE avg_dp
## WGATRUE     -0.882
```

```
## avg_depth   -0.988  0.888
## WGATRUE:vg_  0.920 -0.992  -0.931
```

```r
plot(het_avg_model)
```

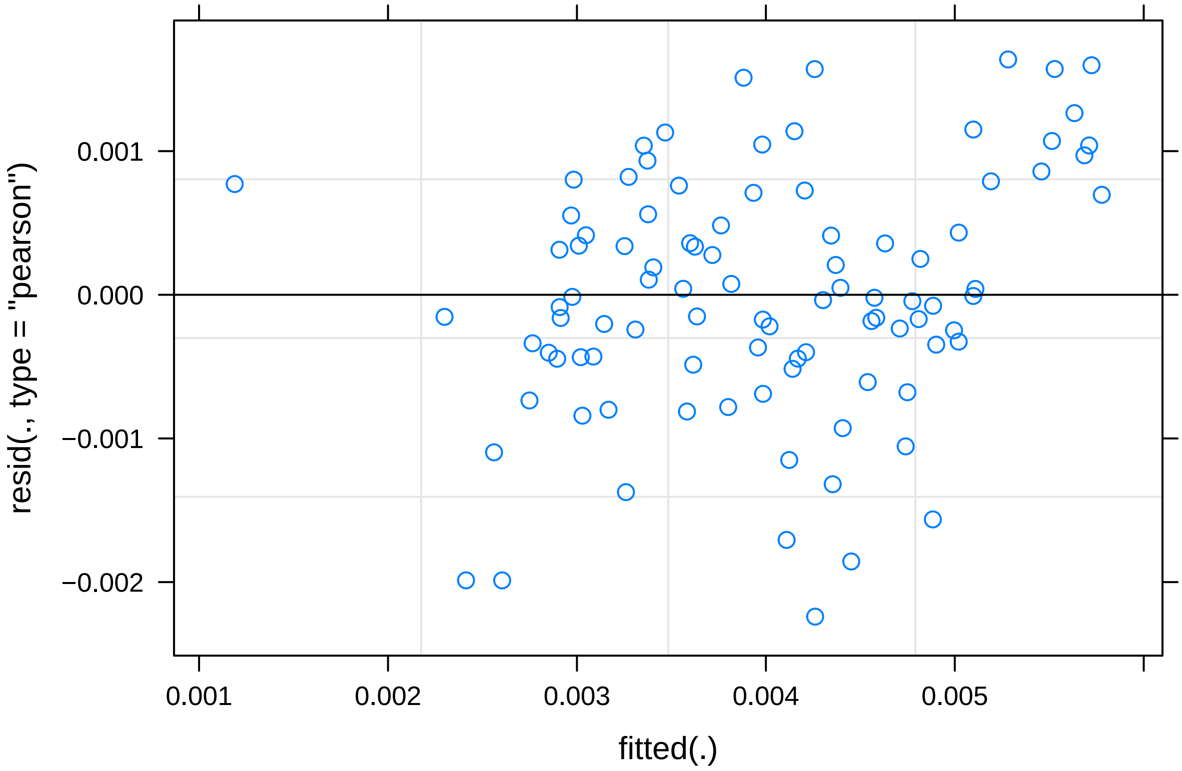


```r
qqnorm(resid(het_avg_model), main = "Q-Q plot for residuals")
```

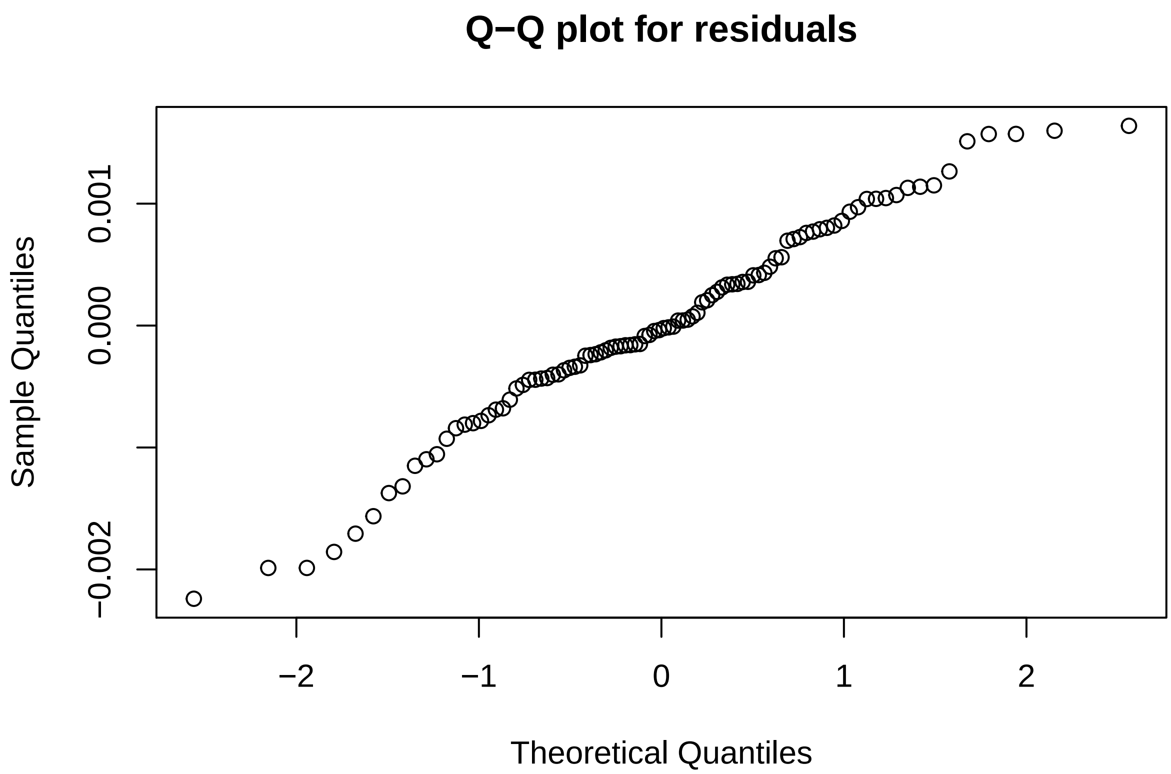


```
het_clust_model_full = lmer(het ~ WGA * Nclusters + (1 | taxon/sample),
    data = prun_sample_info_dup, REML = TRUE)
step_out = step(het_clust_model_full, reduce.random = FALSE)
step_out
```

```
## Backward reduced random-effect table:
##
##                      Eliminated npar logLik     AIC     LRT Df Pr(>Chisq)
## <none>                             7 497.03 -980.07
## (1 | sample:taxon)            0    6 495.27 -978.54  3.5299  1  0.0602718
## (1 | taxon)                   0    6 491.34 -970.68 11.3874  1  0.0007394
##
## <none>
## (1 | sample:taxon) .
## (1 | taxon)        ***
## ---
## Signif. codes:  0 '***' 0.001 '**' 0.01 '*' 0.05 '.' 0.1 ' ' 1
##
## Backward reduced fixed-effect table:
## Degrees of freedom method: Satterthwaite
##
##                 Eliminated     Sum Sq    Mean Sq NumDF  DenDF F value
## WGA:Nclusters            0 4.3103e-06 4.3103e-06     1 70.926  6.1439
##                  Pr(>F)
## WGA:Nclusters 0.01557 *
## ---
## Signif. codes:  0 '***' 0.001 '**' 0.01 '*' 0.05 '.' 0.1 ' ' 1
##
## Model found:
## het ~ WGA * Nclusters + (1 | taxon/sample)
```

```r
het_clust_model = get_model(step_out)
summary(het_clust_model)
```

```
## Linear mixed model fit by REML. t-tests use Satterthwaite's method [
## lmerModLmerTest]
## Formula: het ~ WGA * Nclusters + (1 | taxon/sample)
##    Data: prun_sample_info_dup
##
## REML criterion at convergence: -994.1
##
## Scaled residuals:
##      Min       1Q   Median       3Q      Max
## -2.19826 -0.65334  0.02357  0.50078  2.48808
##
## Random effects:
##  Groups       Name        Variance  Std.Dev.
##  sample:taxon (Intercept) 2.862e-07 0.0005350
##  taxon        (Intercept) 3.584e-07 0.0005987
##  Residual                 7.016e-07 0.0008376
## Number of obs: 96, groups:  sample:taxon, 48; taxon, 5
##
## Fixed effects:
##                      Estimate Std. Error         df t value Pr(>|t|)
## (Intercept)       -0.0007666  0.0012028 78.6429947  -0.637 0.525779
## WGATRUE           -0.0071734  0.0027802 70.8846789  -2.580 0.011948 *
## Nclusters          0.0005649  0.0001445 77.4919294   3.909 0.000197 ***
## WGATRUE:Nclusters  0.0007690  0.0003103 70.9260737   2.479 0.015567 *
## ---
## Signif. codes:  0 '***' 0.001 '**' 0.01 '*' 0.05 '.' 0.1 ' ' 1
##
## Correlation of Fixed Effects:
##             (Intr) WGATRUE Nclstr
## WGATRUE     -0.494
## Nclusters   -0.966  0.511
## WGATRUE:Ncl  0.530 -0.997  -0.552
```

```r
plot(het_clust_model)
```

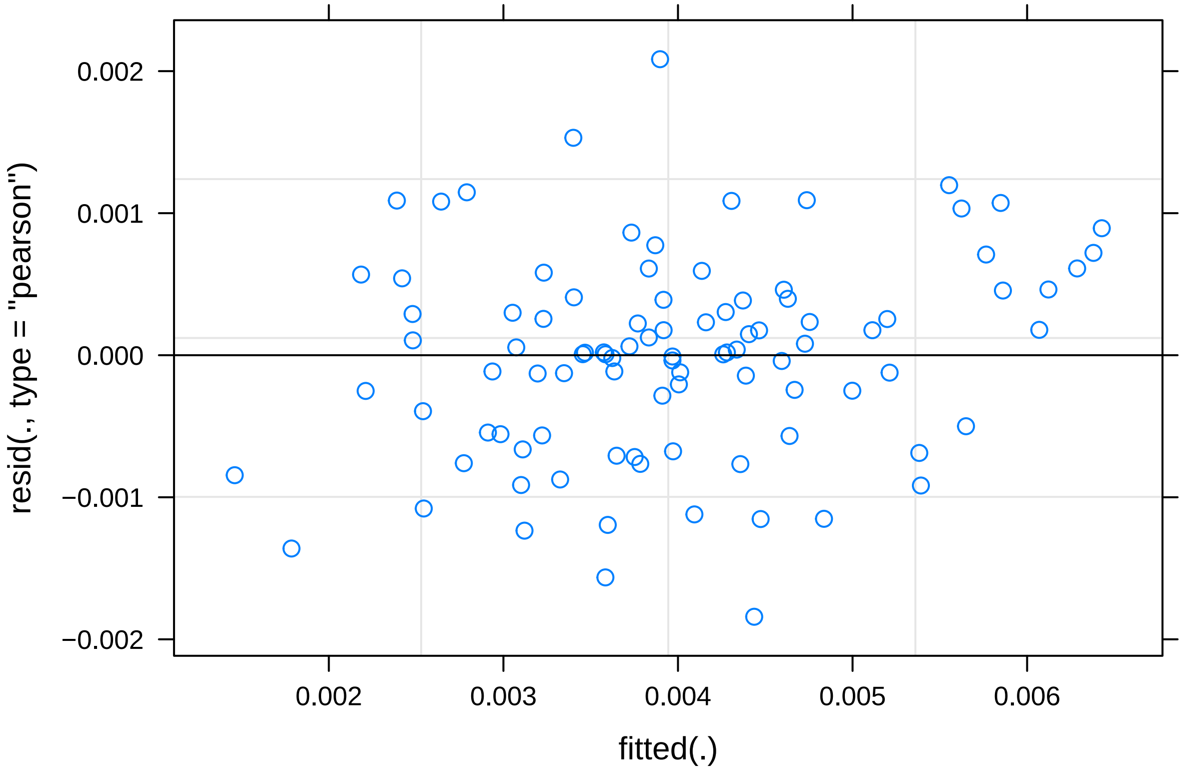


```r
qqnorm(resid(het_clust_model), main = "Q-Q plot for residuals")
```

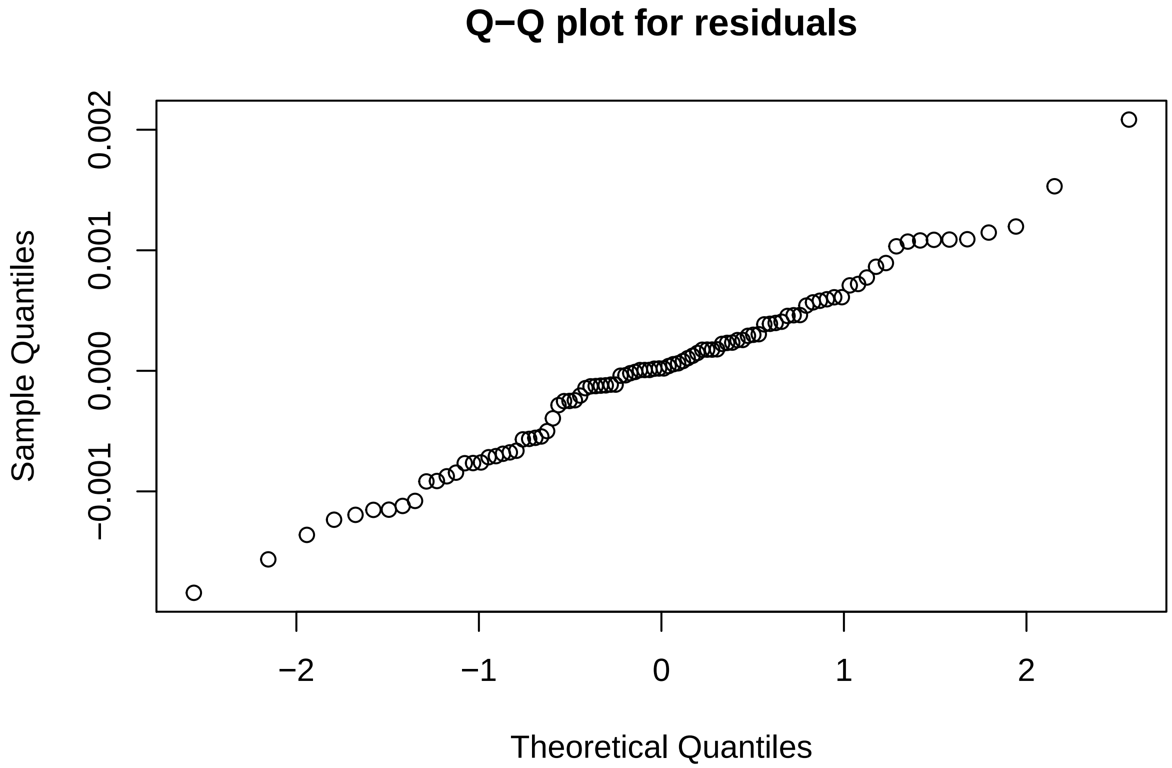

In the case of average depth, the interaction was dropped. In the case of number of clusters, the full model was kept.

Let's visualize the effects in both models:

```
line_seq = log(seq(1, 25000, 100))
line_df = data.frame(Nclusters = line_seq, WGA = rep(c("TRUE",
    "FALSE"), each = length(line_seq)))
prediction = cbind(line_df, x = exp(line_seq), y = predict(het_clust_model,
    line_df, re.form = NA))

p1 = ggplot(prun_sample_info_dup) + geom_jitter(aes(x = clusters_hidepth/1000,
    y = N_nucleotides_heteroz/N_nucleotides_s7, colour = WGA)) +
    scale_x_continuous(limits = range(prun_sample_info_dup$clusters_hidepth)/1000 +
        c(-0.1, 0.1)) + scale_y_continuous(limits = c(0, max(prun_sample_info_dup$N_nucleotides_heteroz/
    5e-04)) + theme_tufte() + theme(panel.border = element_rect(colour = "black",
    fill = NA)) + scale_colour_manual(values = brewer.pal(n = 3,
    name = "RdYlBu")[c(3, 1)], name = "", labels = c(`FALSE` = "gDNA",
    `TRUE` = "MDA")) + ylab("Observed heterozygosity") + xlab("Assembled loci (thousands)") +
    geom_line(data = prediction, aes(x = x/1000, y = y, colour = WGA))


print(p1)
```

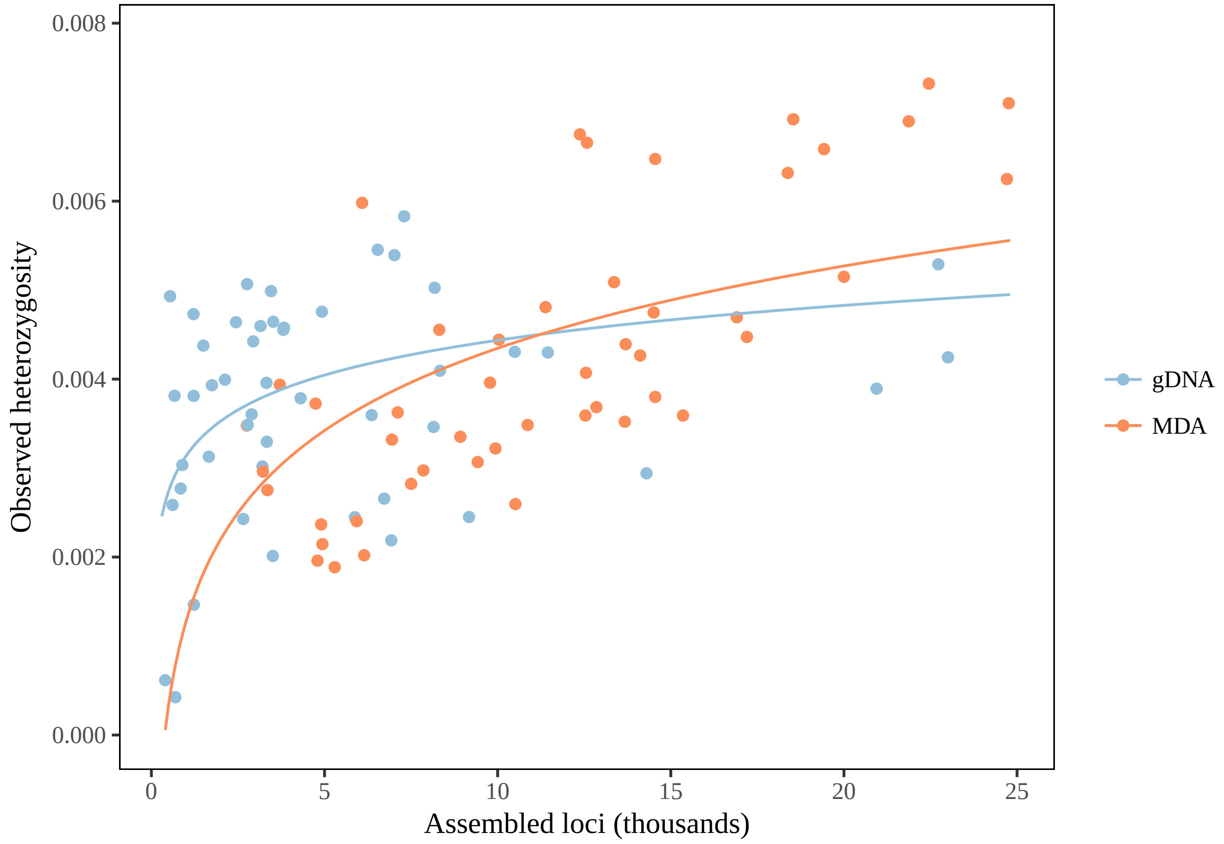

Now let's look at the effect of average coverage alone.

```
line_seq = log(seq(5, 300, by = 0.1))
line_df = data.frame(avg_depth = line_seq, WGA = rep(c("TRUE",
    "FALSE"), each = length(line_seq)))
prediction = cbind(line_df, x = exp(line_seq), y = predict(het_avg_model,
    line_df, re.form = NA))
```

```
p2 = ggplot(prun_sample_info_dup) + geom_jitter(aes(x = avg_depth_stat,
    y = N_nucleotides_heteroz/N_nucleotides_s7, colour = WGA)) +
    scale_x_continuous(limits = range(prun_sample_info_dup$avg_depth_stat) +
        c(-5, 10)) + scale_y_continuous(limits = c(0, max(prun_sample_info_dup$N_nucleotides_heteroz/pru
    5e-04)) + theme_tufte() + theme(panel.border = element_rect(colour = "black",
    fill = NA)) + scale_colour_manual(values = brewer.pal(n = 3,
    name = "RdYlBu")[c(3, 1)], name = "", labels = c(`FALSE` = "gDNA",
    `TRUE` = "MDA")) + ylab("Observed heterozygosity") + xlab("Average coverage in assembled loci") +
    geom_line(data = prediction, aes(x = x, y = y, colour = WGA))


print(p2)
```

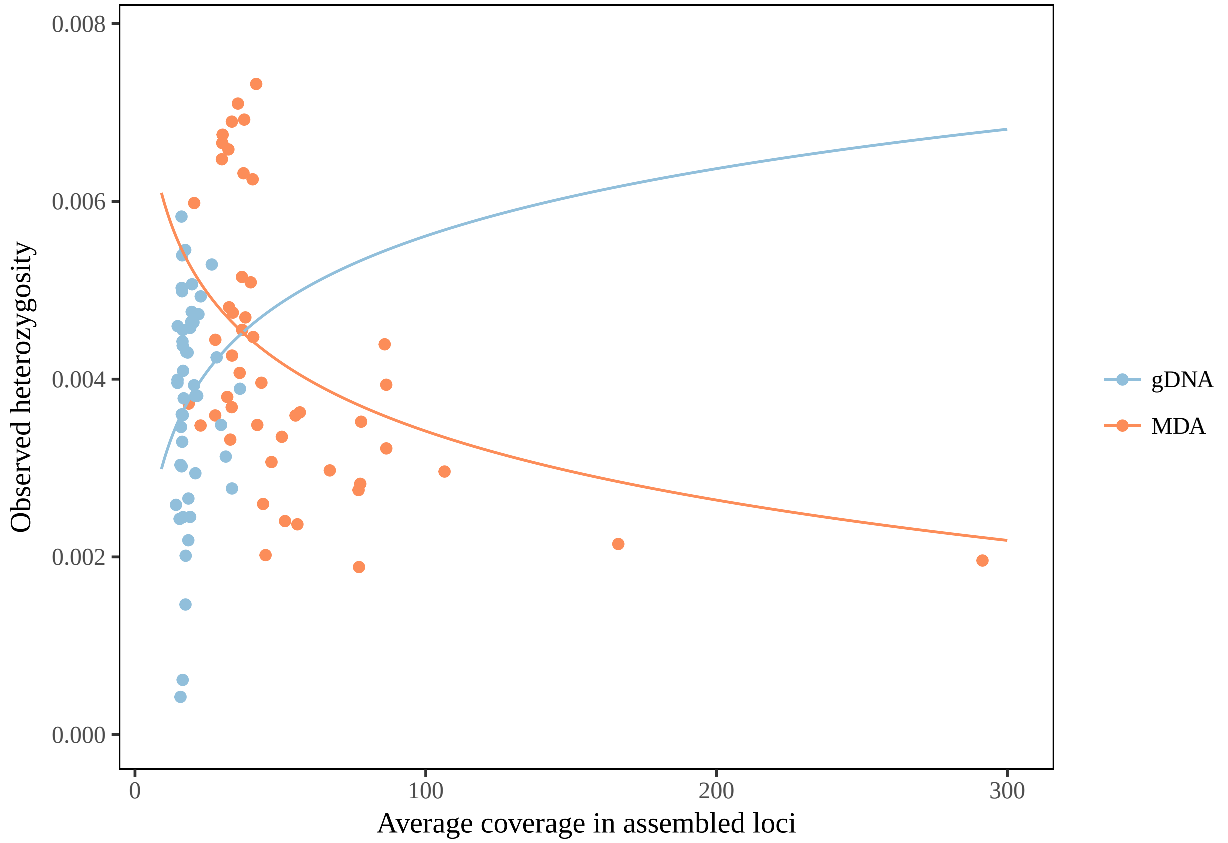

Finally, let's plot the relationship between assembled loci and average coverage:

```
p3 = ggplot(prun_sample_info_dup) + geom_point(aes(x = clusters_hidepth/1000,
    y = avg_depth_stat, color = WGA)) + theme_tufte() + theme(panel.border = element_rect(colour = "bla
    fill = NA)) + scale_colour_manual(values = brewer.pal(n = 3,
    name = "RdYlBu")[c(3, 1)], name = "", labels = c(`FALSE` = "gDNA",
    `TRUE` = "MDA")) + ylab("Average coverage in assembled loci") +
    xlab("Assembled loci (thousands)")

print(p3)
```

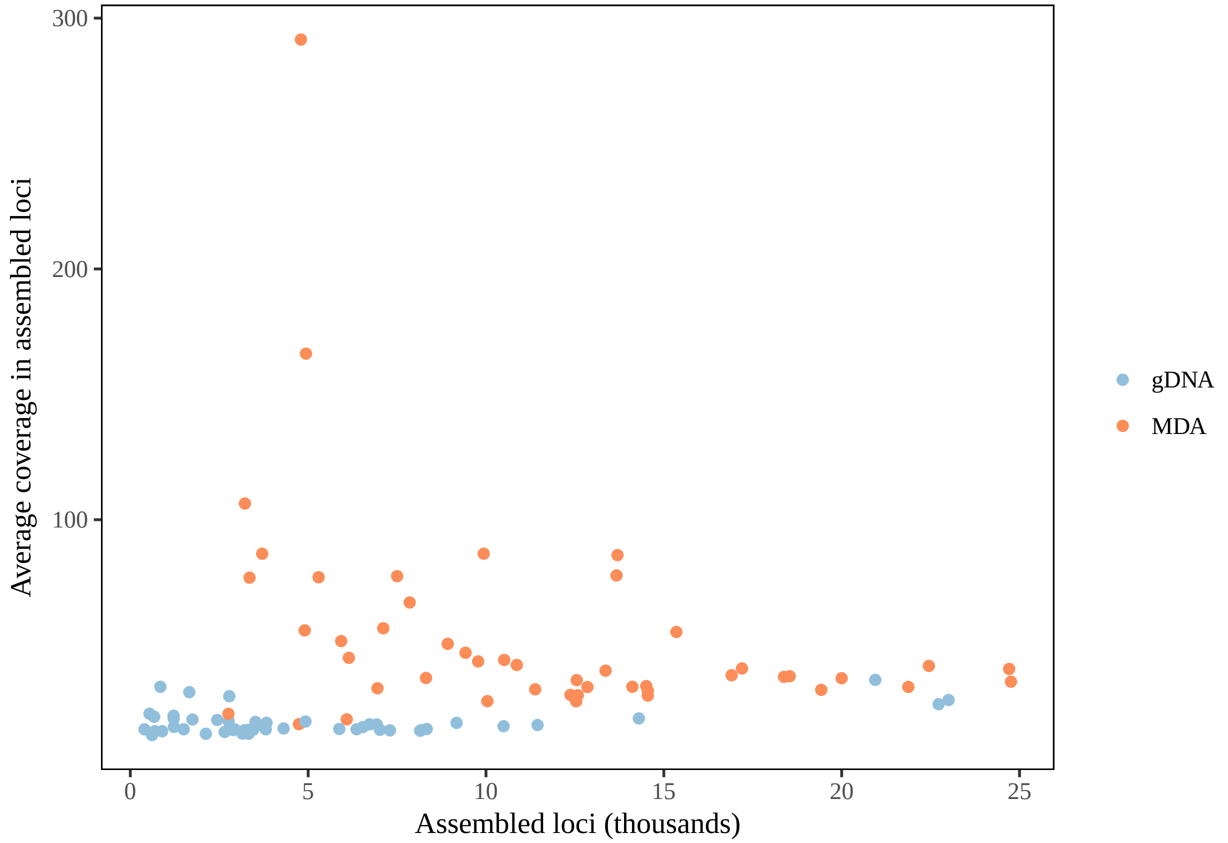

Now let's plot the 3 of them together:

```
# extract legend
# https://github.com/hadley/ggplot2/wiki/Share-a-legend-between-two-ggplot2-graphs
g_legend <- function(a.gplot) {
    tmp <- ggplot_gtable(ggplot_build(a.gplot))
    leg <- which(sapply(tmp$grobs, function(x) x$name) == "guide-box")
    legend <- tmp$grobs[[leg]]
    return(legend)
}

my_legend = g_legend(p1)

p = grid.arrange(p1 + theme(legend.position = "none"), p3 + theme(legend.position = "none"),
    p2 + theme(legend.position = "none"), my_legend, layout_matrix = matrix(c(1,
        1, 1, 2, 2, 2, 3, 3, 3, 4), nrow = 1))
```

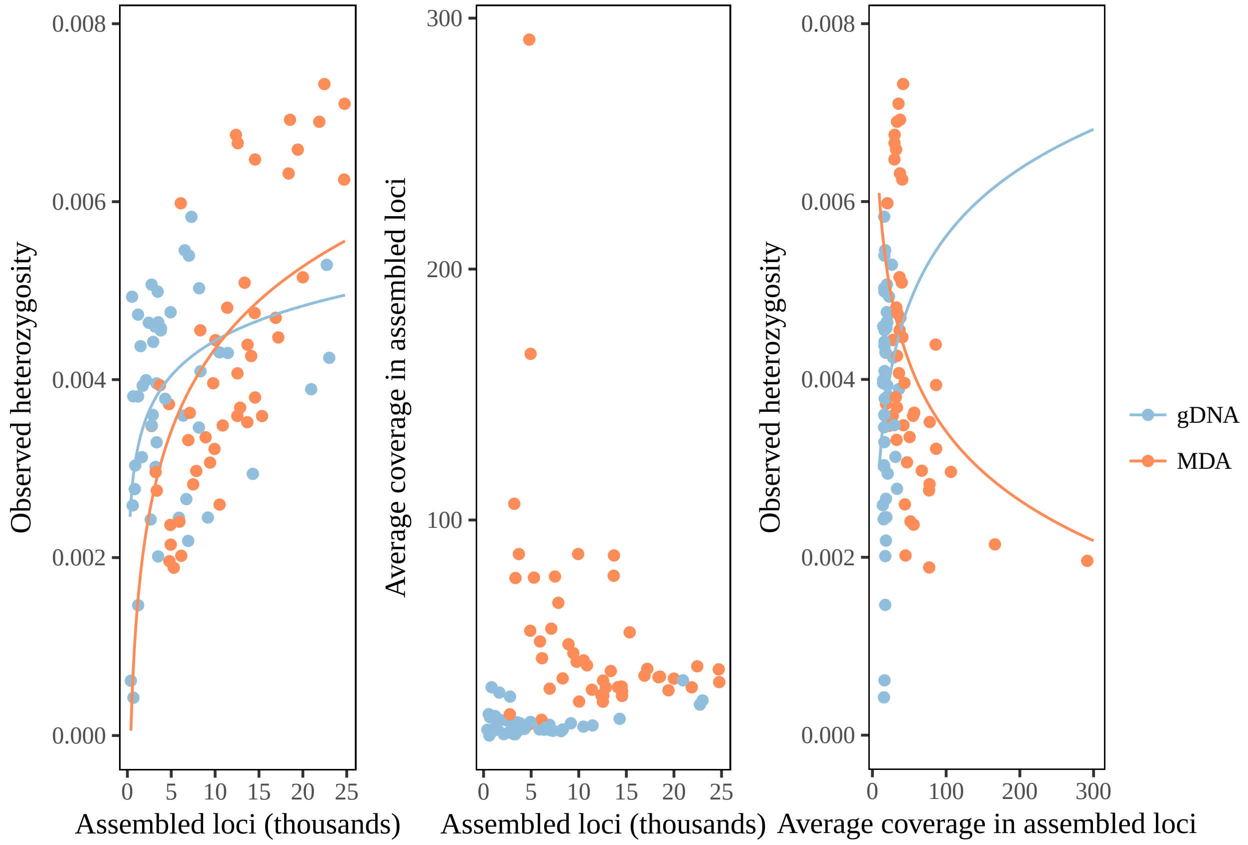


```
ggsave(filename = "plots/fig_het.pdf", device = "pdf", width = 8,
    height = 3, plot = p, useDingbats = F)
```

4 - does WGA bias the GC content?

It seems MDA does not change GC content.

There is a small effect of number of loci. If Illumina sequencing preferentially sequences GC-rich fragments, we should expect some GC enrichment in low-coverage samples.

Model fitting:

```
GC_data = prun_sample_info_dup
GC_data$Nclusters = log(GC_data$clusters_hidepth)
GC_data$GC = GC_data$GC_content
GC_model_full = lmer(GC ~ Nclusters + WGA + Nclusters:WGA + (1 |
    taxon/sample), data = GC_data)

step_out = step(GC_model_full, reduce.random = FALSE)
GC_model = get_model(step_out)
summary(GC_model)
```

```
## Linear mixed model fit by REML. t-tests use Satterthwaite's method [
## lmerModLmerTest]
## Formula: GC ~ Nclusters + (1 | taxon/sample)
##    Data: GC_data
##
## REML criterion at convergence: -770.5
```

```
##
## Scaled residuals:
##     Min      1Q  Median      3Q     Max
## -2.3101 -0.6142  0.0282  0.5293  3.2939
##
## Random effects:
##  Groups       Name        Variance  Std.Dev.
##  sample:taxon (Intercept) 0.000e+00 0.000000
##  taxon        (Intercept) 1.512e-04 0.012295
##  Residual                 1.169e-05 0.003419
## Number of obs: 96, groups:  sample:taxon, 48; taxon, 5
##
## Fixed effects:
##                 Estimate Std. Error         df t value Pr(>|t|)
## (Intercept)  0.3376543  0.0063328  6.9463915  53.319 2.46e-10 ***
## Nclusters   -0.0012506  0.0003627 90.0406621  -3.449 0.000859 ***
## ---
## Signif. codes:  0 '***' 0.001 '**' 0.01 '*' 0.05 '.' 0.1 ' ' 1
##
## Correlation of Fixed Effects:
##           (Intr)
## Nclusters -0.492
```

```
plot(GC_model)
```

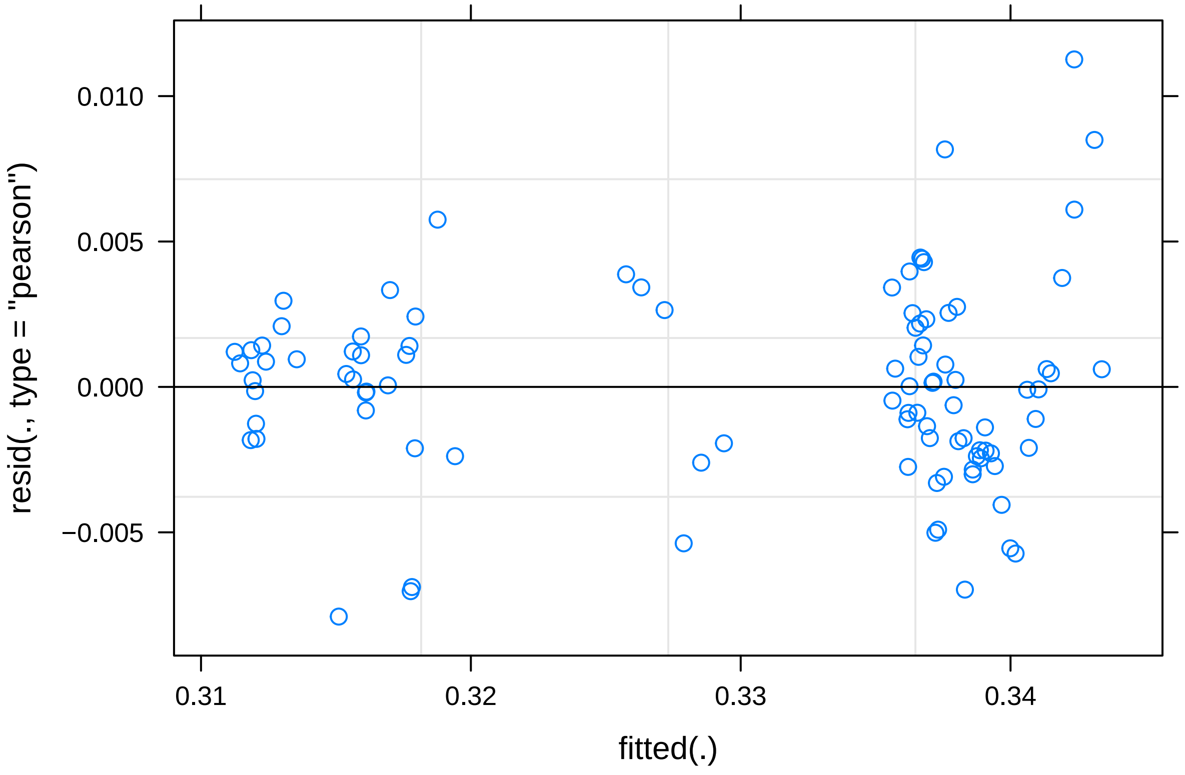


```
qqnorm(resid(GC_model), main = "Q-Q plot for residuals")
```

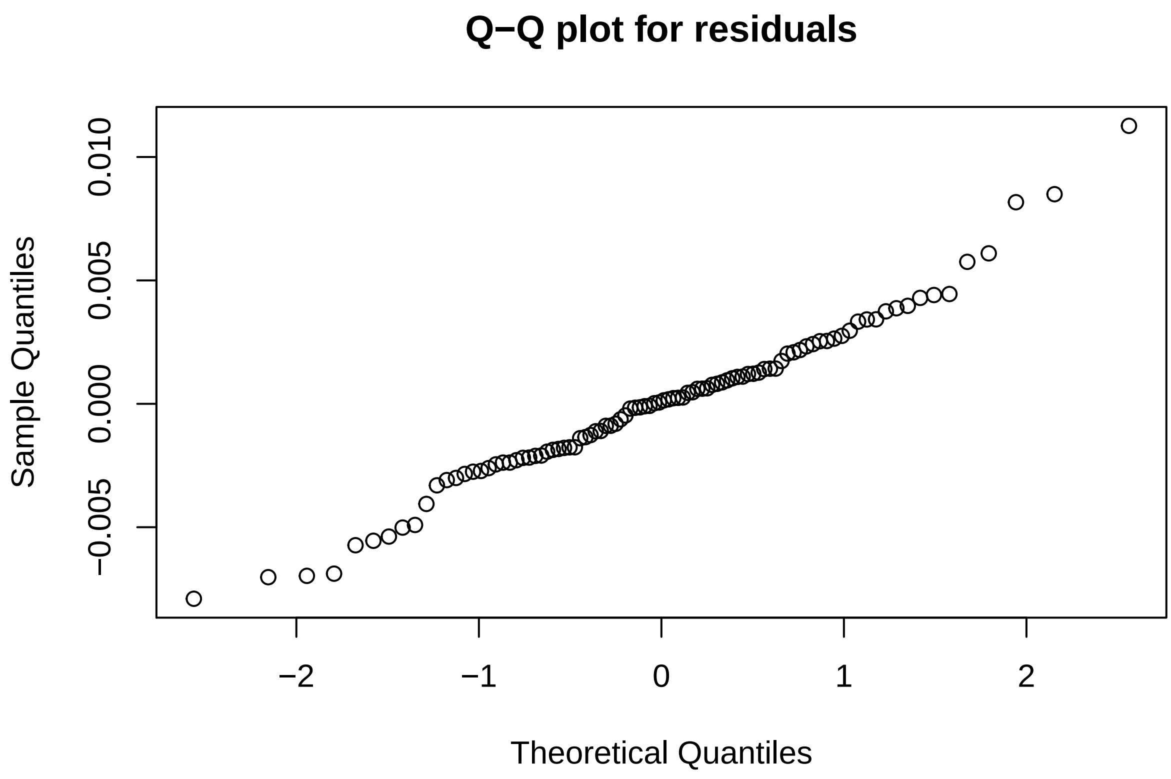

Plot:

```
line_seq = log(seq(1, 25000, 100))
line_df = data.frame(Nclusters = line_seq)
prediction = cbind(line_df, x = exp(line_seq), y = predict(GC_model,
    line_df, re.form = NA))


p = ggplot(prun_sample_info_dup) + geom_jitter(aes(x = clusters_hidepth/1000,
    y = GC_content, colour = WGA)) + scale_x_continuous(limits = range(prun_sample_info_dup$clusters_hid
    c(-0.1, 0.1)) + theme_tufte() + theme(panel.border = element_rect(colour = "black",
    fill = NA)) + scale_colour_manual(values = brewer.pal(n = 3,
    name = "RdYlBu")[c(3, 1)], name = "", labels = c(`FALSE` = "gDNA",
    `TRUE` = "MDA")) + ylab("%GC") + xlab("Assembled loci (thousands)") +
    geom_line(data = prediction, aes(x = x/1000, y = y))  #+

print(p)
```

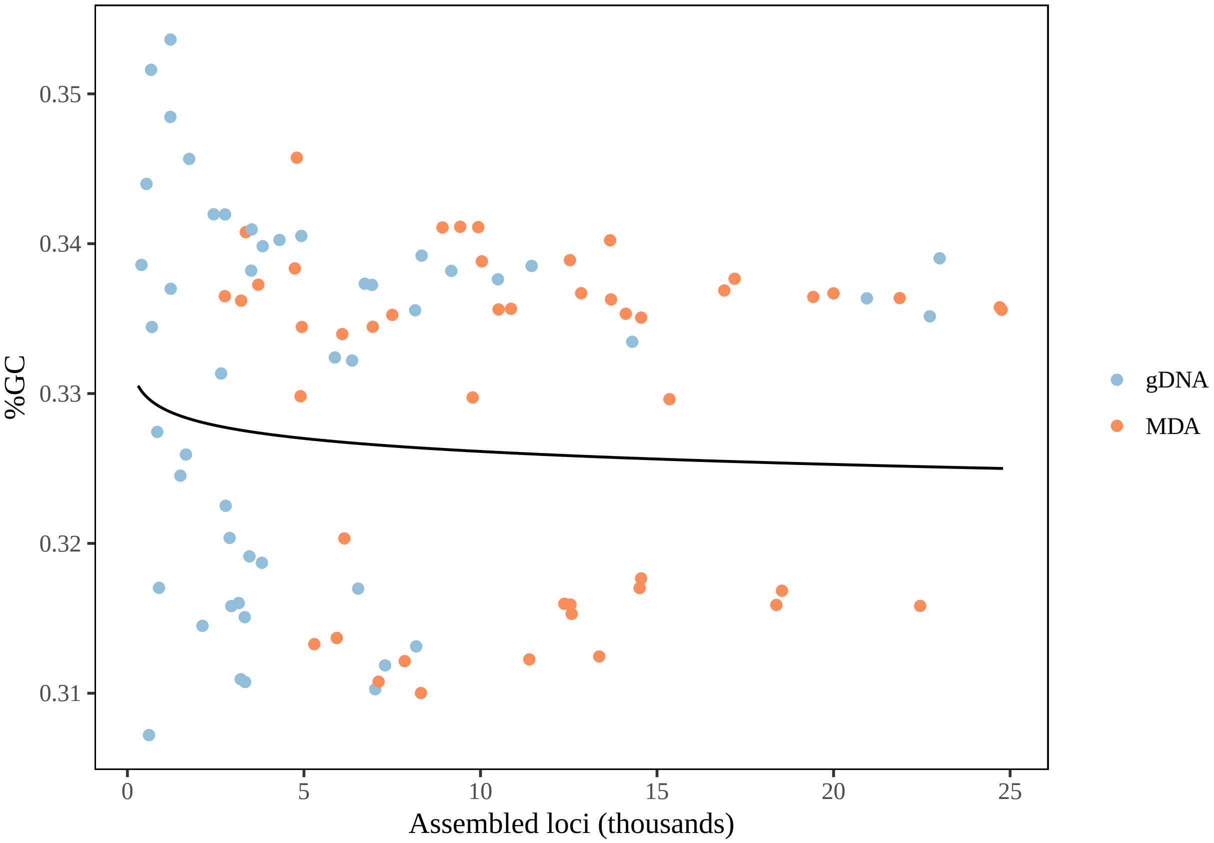


```
pdf("plots/fig_gc.pdf", width = 7, height = 3.5, useDingbats = F)
print(p)
dev.off()
```

```
## pdf
##   2
```

Are errors and biases caused by MDA large enough to impair analyses?

Considering all the above, we are now interested in knowing whether samples that have undergone MDA would erroneuosly cluster together if we do clustering by genetic similarity. In order to do that, we will use MDMR, a regression method in which the response is a pairwise distance matrix.

Here we computed the pairwise genetic distance between all samples. In the python script, we actually calculated, for each site, `2-(number of shared alleles)`, and averaged over all sites. Here we divide this score by 2, so that the distance is in the scale 0-1.

First, prepare data frame for plotting and data analysis.

```
plot_df = data.frame(sample = NULL, pool = NULL, samplename_ipyrad = NULL,
    taxon = NULL, population = NULL, WGA_from = NULL, WGA_to = NULL,
    Nloci = NULL, ave_gaps = NULL, ave_matches = NULL)


for (i in 1:dim(prun_sample_info_dup)[1]) {
    sample = prun_sample_info_dup$sample[i]
```

```
    fullname_i = paste(sample, "pool", sprintf("%02s", prun_sample_info_dup$pool[i]),
        sep = "")

    # first compare statistics to self
    other = setdiff(which(prun_sample_info_dup$sample == sample),
        i)
    fullname_other = paste(sample, "pool", sprintf("%02s", prun_sample_info_dup$pool[other]),
        sep = "")
    loci = pairwise_loci[fullname_i, fullname_other]
    matches = pairwise_distances[fullname_i, fullname_other]

    # then to other samples in the same population
    population = prun_sample_info_dup$population[i]
    taxon = prun_sample_info_dup$taxon[i]

    # WGA true
    criterion_dup = prun_sample_info_dup$sample != as.character(sample) &
        prun_sample_info_dup$taxon == as.character(taxon) & prun_sample_info_dup$population ==
        as.character(population) & prun_sample_info_dup$WGA ==
        TRUE
    temp_df = prun_sample_info_dup[criterion_dup, ]
    criterion_sing = sample_info_sing$sample != as.character(sample) &
        sample_info_sing$taxon == as.character(taxon) & sample_info_sing$population ==
        as.character(population) & sample_info_sing$WGA == TRUE
    temp_df = rbind(temp_df, sample_info_sing[criterion_sing,
        ])

    temp_loci = c()
    temp_distances = c()
    for (j in 1:dim(temp_df)[1]) {
        fullname_other = paste(temp_df$sample[j], "pool", sprintf("%02s",
            temp_df$pool[j]), sep = "")
        temp_loci = c(temp_loci, pairwise_loci[fullname_i, fullname_other])
        temp_distances = c(temp_distances, pairwise_distances[fullname_i,
            fullname_other])
    }
    plot_df = rbind(plot_df, data.frame(sample = sample, pool = as.character(prun_sample_info_dup$pool[i
        samplename_ipyrad = as.character(prun_sample_info_dup$samplename_ipyrad[i]),
        taxon = as.character(prun_sample_info_dup$taxon[i]),
        population = as.character(prun_sample_info_dup$population[i]),
        WGA_from = prun_sample_info_dup$WGA[i], WGA_to = as.character(TRUE),
        Nloci = mean(temp_loci, na.rm = T), ave_matches = mean(temp_distances,
            na.rm = T)))


    # WGA false
    criterion_dup = prun_sample_info_dup$sample != as.character(sample) &
        prun_sample_info_dup$taxon == as.character(taxon) & prun_sample_info_dup$population ==
        as.character(population) & prun_sample_info_dup$WGA ==
        FALSE
    temp_df = prun_sample_info_dup[criterion_dup, ]
    criterion_sing = sample_info_sing$sample != as.character(sample) &
        sample_info_sing$taxon == as.character(taxon) & sample_info_sing$population ==
```

```
        as.character(population) & sample_info_sing$WGA == FALSE
    temp_df = rbind(temp_df, sample_info_sing[criterion_sing,
        ])

    for (j in 1:dim(temp_df)[1]) {
        fullname_other = paste(temp_df$sample[j], "pool", sprintf("%02s",
            temp_df$pool[j]), sep = "")
        temp_loci = c(temp_loci, pairwise_loci[fullname_i, fullname_other])
        temp_distances = c(temp_distances, pairwise_distances[fullname_i,
            fullname_other])
    }
    plot_df = rbind(plot_df, data.frame(sample = sample, pool = as.character(prun_sample_info_dup$pool[
        samplename_ipyrad = as.character(prun_sample_info_dup$samplename_ipyrad[i]),
        taxon = as.character(prun_sample_info_dup$taxon[i]),
        population = as.character(prun_sample_info_dup$population[i]),
        WGA_from = prun_sample_info_dup$WGA[i], WGA_to = as.character(FALSE),
        Nloci = mean(temp_loci, na.rm = T), ave_matches = mean(temp_distances,
            na.rm = T)))
}

plot_df[is.na(plot_df)] = 0
```

Here we will use the matrix of pairwise genetic distances as a response, and MDA, population and number of loci as predictors. The model will be fit separately for each taxon using MDMR.

MDA is not significant in any case. The closest to significance is Anchylorhynchus (0.126 when I ran it for the article, but may vary slightly below due to random permutation). In any case, the pseudo R2 is very small in this case (0.009), so MDA does not seem to be a major factor in clustering.

```
for (taxon in c("Anchylorhynchus", "Andranthobius", "Celetes_impar",
    "Microstrates_bondari", "Microstrates_ypsilon")) {
    samples = as.character(prun_sample_info_dup$samplename_ipyrad[prun_sample_info_dup$taxon ==
        taxon])

    # some samples do not have any loci in common, removing those
    gendist = pairwise_distances[samples, samples]
    na_rows = apply(gendist, 1, function(x) {
        any(is.na(x))
    })
    na_samples = sapply(names(na_rows)[which(na_rows)], function(x) {
        strsplit(x, "pool")[[1]][1]
    })
    if (length(na_samples)) {
        rows_to_remove = grepl(paste(na_samples, collapse = "|"),
            rownames(gendist))
        gendist = gendist[!rows_to_remove, !rows_to_remove]
    }

    gendist = as.dist(gendist)

    samples = labels(gendist)

    cat(paste("\n\n\n", "Number of libraries", length(samples),
        ":\n"))
```

```
    predictors = prun_sample_info_dup[match(samples, prun_sample_info_dup$samplename_ipyrad),
        c("WGA", "population", "N_loci_s7")]


    variables = colnames(predictors)
    predictors = data.frame(factor(predictors[[1]]), factor(predictors[[2]]),
        log(as.integer(predictors[[3]])))
    colnames(predictors) = variables
    rownames(predictors) = samples

    mdmr_model = mdmr(D = gendist, X = predictors)
    cat(paste("\n\n\n", taxon, "\n\n"))
    summary(mdmr_model)


}
```

```
##
##
##
##  Number of libraries 32 :
## 100 % of permutation test statistics computed.
##
##
##
##  Anchylorhynchus
##
##             Statistic Numer.DF Pseudo.R2 Permutation.p.value
## (Omnibus)     9.2604       10   0.90254              <0.002 ***
## WGA1          0.0911        1   0.00888               0.164
## population    8.7239        8   0.85025              <0.002 ***
## N_loci_s7     0.0444        1   0.00433               0.416
## ---
## Signif. codes:  0 "***" 0.001 "**" 0.01 "*" 0.05 "." 0.1 " " 1
##
##
##  Number of libraries 6 :
## 100 % of permutation test statistics computed.
##
##
##
##  Andranthobius
##
##              Statistic Numer.DF Pseudo.R2 Permutation.p.value
## (Omnibus)      1.1860        3    0.5426               0.644
## WGA1           0.1427        1    0.0653               0.860
## population1    0.8382        1    0.3834               0.196
## N_loci_s7      0.0776        1    0.0355               0.934
## ---
## Signif. codes:  0 "***" 0.001 "**" 0.01 "*" 0.05 "." 0.1 " " 1
##
##
##  Number of libraries 20 :
```

```
## 100 % of permutation test statistics computed.
##
##
##
##  Celetes_impar
##
##            Statistic Numer.DF Pseudo.R2 Permutation.p.value
## (Omnibus)     1.8571        6    0.6500              <0.002 ***
## WGA1          0.0378        1    0.0132               0.850
## population    1.7311        4    0.6059              <0.002 ***
## N_loci_s7     0.0862        1    0.0302               0.336
## ---
## Signif. codes:  0 "***" 0.001 "**" 0.01 "*" 0.05 "." 0.1 " " 1
##
##
##  Number of libraries 18 :
## 100 % of permutation test statistics computed.
##
##
##
##  Microstrates_bondari
##
##            Statistic Numer.DF Pseudo.R2 Permutation.p.value
## (Omnibus)     0.9416        4    0.4850              <0.002 ***
## WGA1          0.0903        1    0.0465               0.316
## population    0.7677        2    0.3954              <0.002 ***
## N_loci_s7     0.1251        1    0.0644               0.076 .
## ---
## Signif. codes:  0 "***" 0.001 "**" 0.01 "*" 0.05 "." 0.1 " " 1
##
##
##  Number of libraries 14 :
## 100 % of permutation test statistics computed.
##
##
##
##  Microstrates_ypsilon
##
##            Statistic Numer.DF Pseudo.R2 Permutation.p.value
## (Omnibus)     1.2832        4    0.5620              <0.002 ***
## WGA1          0.0749        1    0.0328               0.762
## population    0.9337        2    0.4089              <0.002 ***
## N_loci_s7     0.2006        1    0.0879               0.046 *
## ---
## Signif. codes:  0 "***" 0.001 "**" 0.01 "*" 0.05 "." 0.1 " " 1
```

Now let's do hierarchical clustering with method single (aka neighbor-joining) and plot the results:

```
plot_list = list()
for (taxon in c("Anchylorhynchus", "Andranthobius", "Celetes_impar",
    "Microstrates_bondari", "Microstrates_ypsilon")) {
    samples = as.character(prun_sample_info_dup$samplename_ipyrad[prun_sample_info_dup$taxon ==
        taxon])

    # some samples do not have any loci in common, removing those
```

```r
gendist = pairwise_distances[samples, samples]
na_rows = apply(gendist, 1, function(x) {
    any(is.na(x))
})
na_samples = sapply(names(na_rows)[which(na_rows)], function(x) {
    strsplit(x, "pool")[[1]][1]
})
if (length(na_samples)) {
    rows_to_remove = grepl(paste(na_samples, collapse = "|"),
        rownames(gendist))
    gendist = gendist[!rows_to_remove, !rows_to_remove]
}

gendist = as.dist(gendist)

samples = labels(gendist)


predictors = prun_sample_info_dup[match(samples, prun_sample_info_dup$samplename_ipyrad),
    c("WGA", "population", "clusters_hidepth")]


variables = colnames(predictors)
predictors = data.frame(factor(predictors[[1]]), factor(predictors[[2]]),
    log(as.integer(predictors[[3]])))
colnames(predictors) = variables
rownames(predictors) = samples

temp_dendro = as.dendrogram(hclust(gendist, method = "single"))
temp_ddata = dendro_data(temp_dendro, type = "rectangle")
segments = segment(temp_ddata)
segments_tips = segments[segments$yend == 0, ]
tips = label(temp_ddata)
tips$pop = as.character(sample_info_all$population[match(tips$label,
    sample_info_all$samplename_ipyrad)])
tips$sample = sapply(tips$label, function(x) {
    strsplit(as.character(x), "pool")[[1]][1]
})
segments$pop = ""
segments$pop[segments$yend == 0][match(tips$x, segments$xend[segments$yend ==
    0])] = as.character(tips$pop)

taxon_translate = c(Anchylorhynchus = "Anchylorhynchus",
    Andranthobius = "Andranthobius", Celetes_impar = "C. impar",
    Microstrates_bondari = "M. bondari", Microstrates_ypsilon = "M. ypsilon")

plot_temp = ggplot(segments) + geom_segment(aes(x = x, y = y,
    xend = xend, yend = yend, colour = pop), size = 1) +
    theme_dendro() + scale_colour_manual(values = c("#000000",
    brewer.pal(length(unique(tips$pop)), name = "Set3")),
    name = "Population") + ggtitle(bquote(italic(.(taxon_translate[taxon])))) +
    coord_flip() + scale_y_reverse(expand = c(0.2, 0)) +
    geom_text(data = tips, aes(x = x, y = y - 1e-04, label = sample),
```

```
            size = 2, hjust = 0)
    print(plot_temp)
    plot_list[[length(plot_list) + 1]] = plot_temp
}
```

Anchylorhynchus

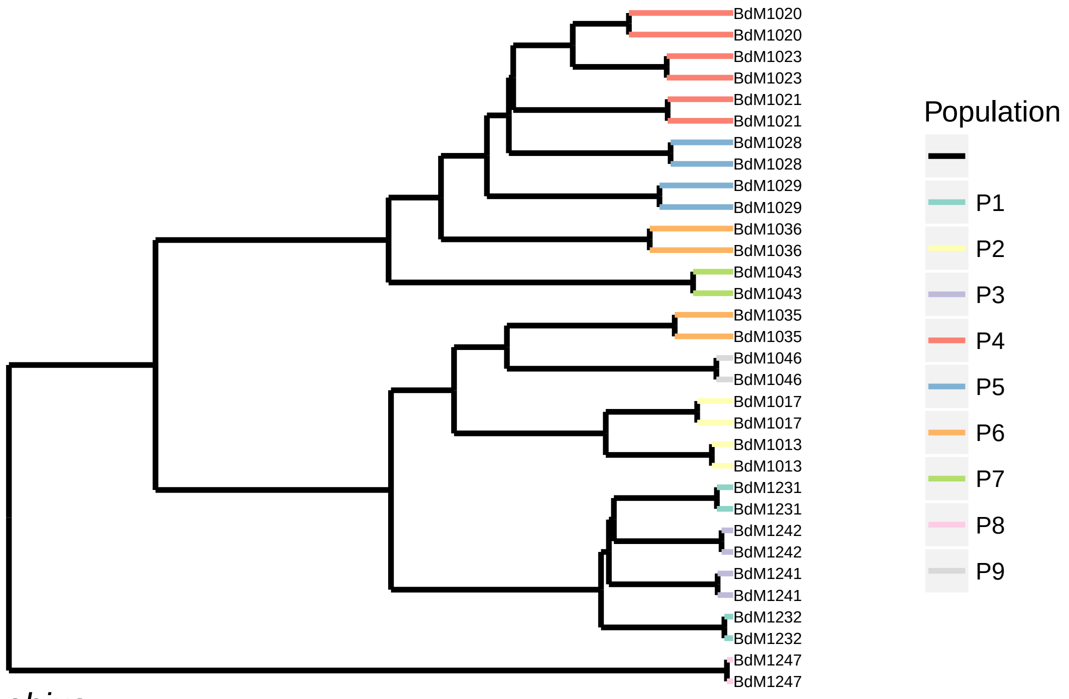

Andranthobius

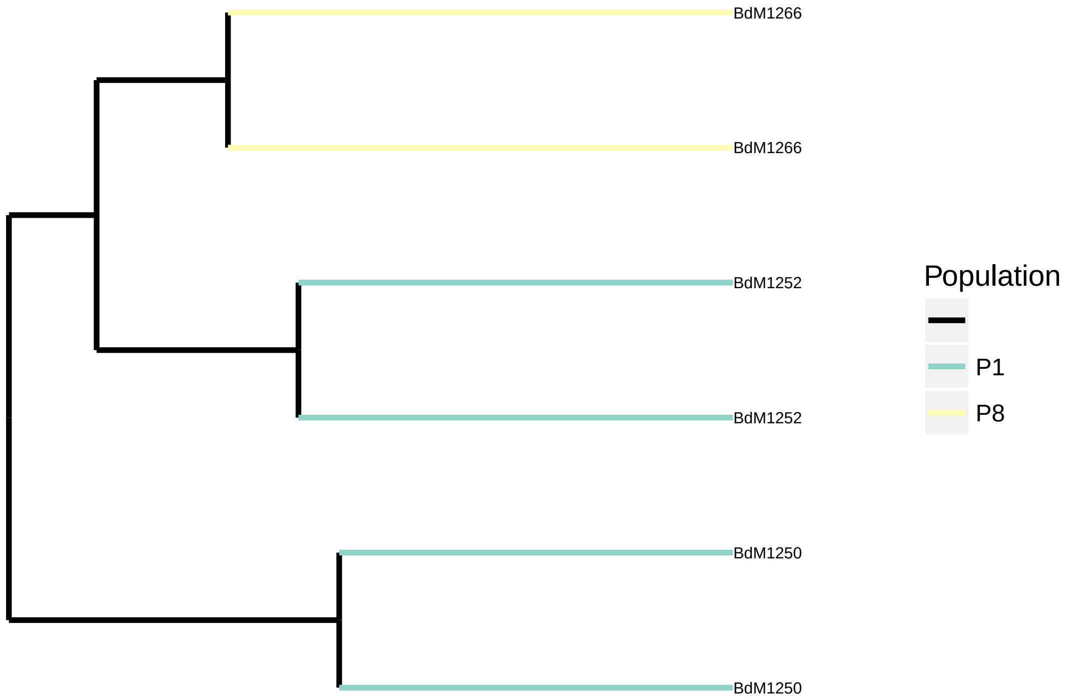

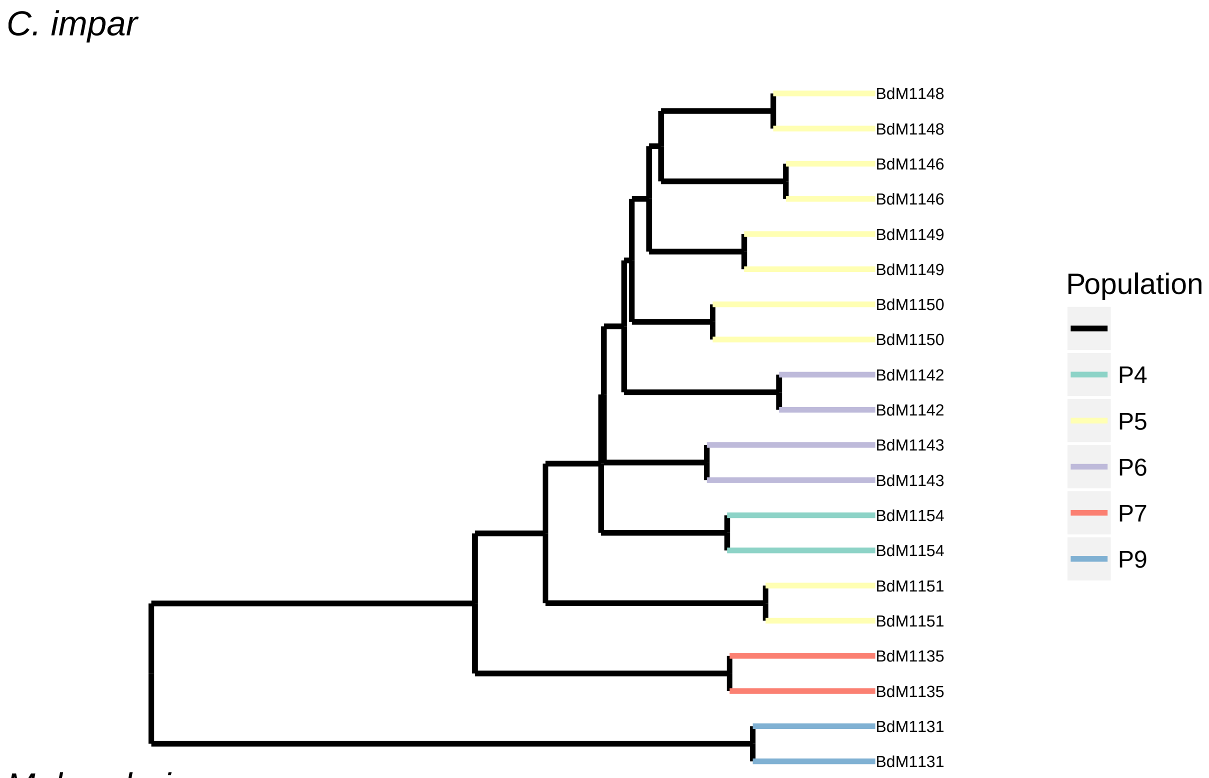
C. impar
BdM1148
BdM1148
BdM1146
BdM1146
BdM1149
BdM1149
BdM1150
BdM1150
BdM1142
BdM1142
BdM1143
BdM1143
BdM1154
BdM1154
BdM1151
BdM1151
BdM1135
BdM1135
BdM1131
BdM1131
Population
P4
P5
P6
P7
P9

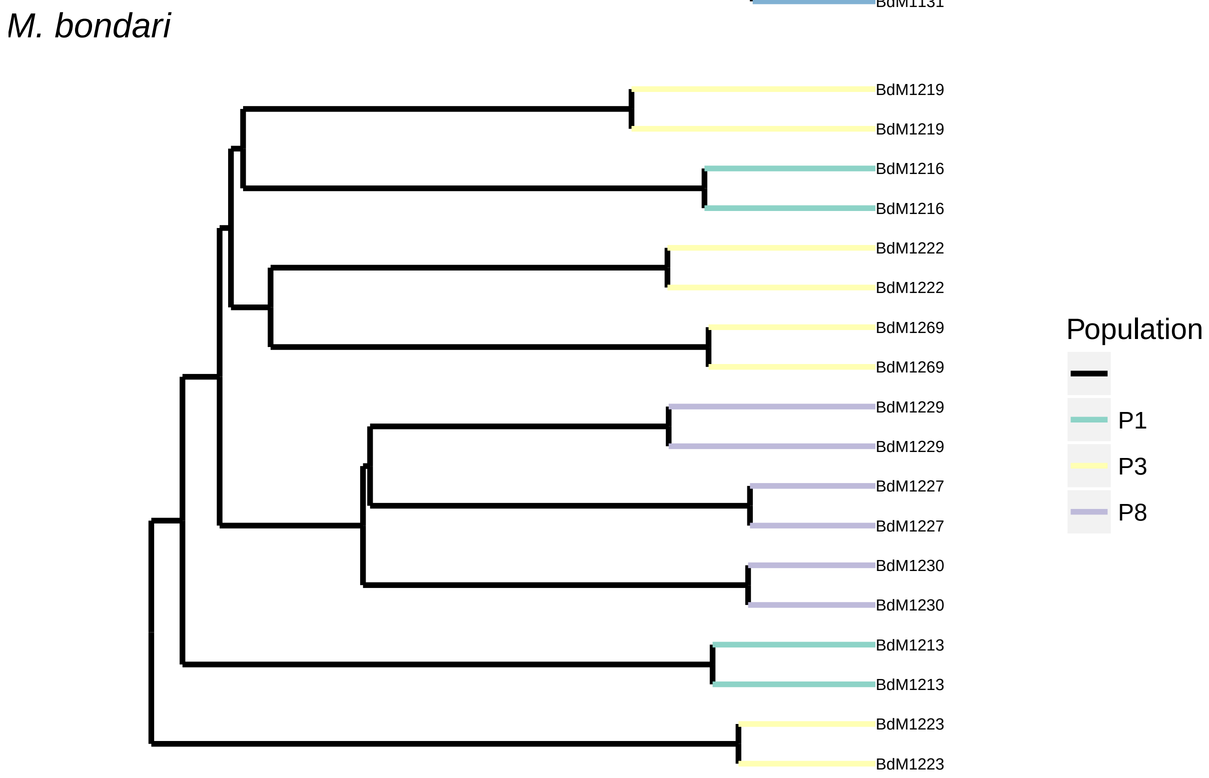
M. bondari
BdM1219
BdM1219
BdM1216
BdM1216
BdM1222
BdM1222
BdM1269
BdM1269
BdM1229
BdM1229
BdM1227
BdM1227
BdM1230
BdM1230
BdM1213
BdM1213
BdM1223
BdM1223
Population
P1
P3
P8

M. ypsilon

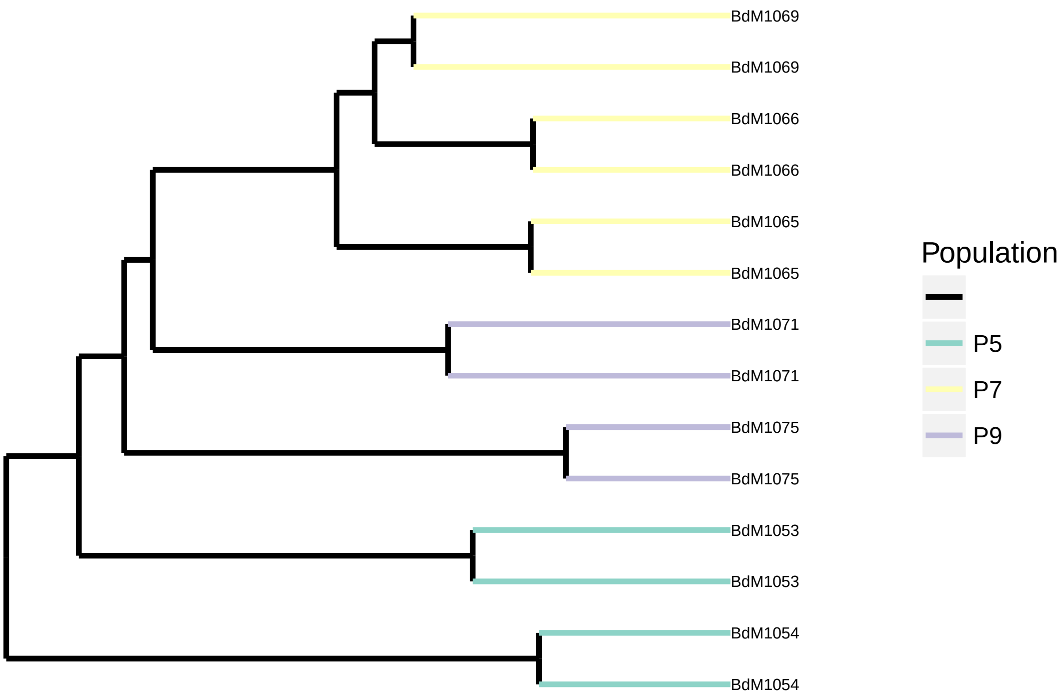

Repeating above, now plotting to a file:

```
pdf("plots/supp_figs.pdf", width = 7.5, height = 10, useDingbats = F)
do.call(grid.arrange, c(plot_list, list(ncol = 2)))
dev.off()
```

pdf 2

Are the loci sequenced with MDA are biased subset of loci sequenced with gDNA libraries?

For each taxon, each kind of library (gDNA or MDA) was typically pooled together for ligation and size selection. Therefore, we expect ligation and size selection effects (i. e. pool effects) to affect all loci sequenced for a given pool. However, since we have multiple pools for each treatment for each taxon, we can try to parse out pool and MDA effects in a MDMR analysis.

Our response pairwise distance variable will be the proportion of loci recovered for just one of the samples in the comparison, and our predictors will be the number of reads, the ligation pool, whether MDA was performed and population. We will include all samples for this analysis, since not sharing any locus is also important information, and we cannot distinguish pool from MDA effects only within paired samples.

```
pairwise_all_loci = read.csv("pairwise_N_loci_total.csv", row.names = 1)

for (taxon1 in c("Anchylorhynchus", "Andranthobius", "Celetes_impar",
    "Microstrates_bondari", "Microstrates_ypsilon")) {
    samples = sample_info %>% filter(taxon == taxon1) %>% select(samplename_ipyrad) %>%
        unlist %>% as.character

    # as.character(prun_sample_info_dup$samplename_ipyrad[prun_sample_info_dup$taxon
```

```r
    # == taxon])

    nloci = pairwise_all_loci[samples, samples]/Nfinal[[taxon1]]
    nloci[is.na(nloci)] = 0
    nloci = as.dist(nloci)
    nloci = 1 - nloci

    # samples = row.names(nloci)

    cat(paste("\n\n\n", "Number of libraries", length(samples),
        ":\n"))


    predictors = sample_info %>% filter(samplename_ipyrad %in%
        samples) %>% transmute(samplename_ipyrad = samplename_ipyrad,
        WGA = factor(WGA), pool = factor(pool), population = factor(population),
        N_loci_s7 = log(as.integer(N_loci_s7))) %>% tibble::remove_rownames() %>%
        tibble::column_to_rownames("samplename_ipyrad")


    # [match(samples,prun_sample_info_dup$samplename_ipyrad),c('WGA','pool','population','N_loci_s7')]


    # variables = colnames(predictors) predictors =
    # data.frame(factor(predictors[[1]]),factor(predictors[[2]]),log(as.integer(predictors[[3]])))
    # colnames(predictors) = variables

    mdmr_model = mdmr(D = nloci, X = predictors)
    cat(paste("\n\n\n", taxon1, "\n\n"))
    summary(mdmr_model)


}
```

```
##
##
##
##  Number of libraries 82 :
## 100 % of permutation test statistics computed.
##
##
##
##  Anchylorhynchus
##
##             Statistic Numer.DF Pseudo.R2 Permutation.p.value
## (Omnibus)     0.3465       14    0.2573              <0.002 ***
## WGA1          0.0200        1    0.0148               0.016 *
## pool          0.0737        4    0.0547              <0.002 ***
## population    0.1690        8    0.1255              <0.002 ***
## N_loci_s7     0.0289        1    0.0214              <0.002 ***
## ---
## Signif. codes:  0 "***" 0.001 "**" 0.01 "*" 0.05 "." 0.1 " " 1
##
##
```

```
##  Number of libraries 27 :
## 100 % of permutation test statistics computed.
##
##
##
##  Andranthobius
##
##            Statistic Numer.DF Pseudo.R2 Permutation.p.value
## (Omnibus)     0.6936        6    0.4095              <0.002 ***
## WGA1          0.0809        1    0.0478               0.016 *
## pool          0.1779        2    0.1050               0.002 **
## population    0.1188        2    0.0701               0.148
## N_loci_s7     0.2253        1    0.1330              <0.002 ***
## ---
## Signif. codes:  0 "***" 0.001 "**" 0.01 "*" 0.05 "." 0.1 " " 1
##
##
##  Number of libraries 43 :
## 100 % of permutation test statistics computed.
##
##
##
##  Celetes_impar
##
##            Statistic Numer.DF Pseudo.R2 Permutation.p.value
## (Omnibus)     0.4105        8    0.2911              <0.002 ***
## WGA1          0.0337        1    0.0239               0.392
## pool1         0.0337        1    0.0239               0.372
## population    0.1713        5    0.1214               0.036 *
## N_loci_s7     0.1120        1    0.0794              <0.002 ***
## ---
## Signif. codes:  0 "***" 0.001 "**" 0.01 "*" 0.05 "." 0.1 " " 1
##
##
##  Number of libraries 33 :
## 100 % of permutation test statistics computed.
##
##
##
##  Microstrates_bondari
##
##            Statistic Numer.DF Pseudo.R2 Permutation.p.value
## (Omnibus)     0.3706        6    0.2704              <0.002 ***
## WGA1          0.0519        1    0.0378               0.050 *
## pool          0.0850        2    0.0620               0.216
## population    0.0910        2    0.0664               0.062 .
## N_loci_s7     0.0953        1    0.0696              <0.002 ***
## ---
## Signif. codes:  0 "***" 0.001 "**" 0.01 "*" 0.05 "." 0.1 " " 1
##
##
##  Number of libraries 31 :
## 100 % of permutation test statistics computed.
##
```

```
##
##
##  Microstrates_ypsilon
##
##             Statistic Numer.DF Pseudo.R2 Permutation.p.value
## (Omnibus)     0.4234        7    0.2975              <0.002 ***
## WGA1          0.0436        1    0.0307               0.616
## pool          0.0915        2    0.0643               0.286
## population    0.1312        3    0.0922               0.442
## N_loci_s7     0.0749        1    0.0527              <0.002 ***
## ---
## Signif. codes:  0 "***" 0.001 "**" 0.01 "*" 0.05 "." 0.1 " " 1
```
