# Supplementary material for: Whole-genome amplification in double-digest RADseq results in adequate libraries but fewer sequenced loci
Source: PeerJ. 2018 Jul 17;6:e5089. doi: 10.7717/peerj.5089 (PMC6054070; doi:10.7717/peerj.5089)
Supplement: Supplemental Information 8 [file peerj-06-5089-s008.pdf]

Article S3: Clustering and isolation by distance

Contents

In this notebook we will do a few simple population genetics analyses to ttest the use of ddRAD libraries prepared with Multiple Displacement Amplification (MDA)

Let's start by loading packages

```
library(adegenet)
```

```
## Loading required package: ade4

##
##    /// adegenet 2.1.1 is loaded ////////////
##
##    > overview: '?adegenet'
##    > tutorials/doc/questions: 'adegenetWeb()'
##    > bug reports/feature requests: adegenetIssues()
```

```
library(BEDASSLE)
library(fossil)
```

```
## Loading required package: sp

## Loading required package: maps

## Loading required package: shapefiles

## Loading required package: foreign

##
## Attaching package: 'shapefiles'

## The following objects are masked from 'package:foreign':
##
##     read.dbf, write.dbf
```

```
library(dplyr)
```

```
##
## Attaching package: 'dplyr'

## The following objects are masked from 'package:stats':
##
##     filter, lag

## The following objects are masked from 'package:base':
##
##     intersect, setdiff, setequal, union
```

```
library(RColorBrewer)
library(ggplot2)
library(broom)
```

```
##
## Attaching package: 'broom'
```

```
## The following object is masked from 'package:fossil':
##
##     bootstrap
```

```
library(data.table)
```

```
##
## Attaching package: 'data.table'

## The following objects are masked from 'package:dplyr':
##
##     between, first, last
```

```
library(ggthemes)
library(gridExtra)
```

```
##
## Attaching package: 'gridExtra'

## The following object is masked from 'package:dplyr':
##
##     combine
```

```
library(vegan)
```

```
## Loading required package: permute

## Loading required package: lattice

## This is vegan 2.5-2
```

```
library(knitr)

knitr::opts_chunk$set(warning=FALSE)
knitr::opts_chunk$set(tidy.opts=list(width.cutoff=60),tidy=TRUE)
```

Now let's load the data:

```
taxa = list.files("./", pattern = ".*gen$") %>% gsub(pattern = "_filtered.*$",
    replacement = "")

genpops = sapply(taxa, function(x) {
    paste(x, ".*gen$", sep = "") %>% list.files(path = "./") %>%
        read.genepop(ncode = 3, quiet = FALSE)
})
```

```
##
##  Converting data from a Genepop .gen file to a genind object...
##
##
## File description:  genepop file generated from Anchylorhynchus_filtered.u.snps.phy.
##
## ...done.
##
##
##  Converting data from a Genepop .gen file to a genind object...
##
##
## File description:  genepop file generated from Andranthobius_filtered.u.snps.phy.
##
```

```
## ...done.
##
##
##  Converting data from a Genepop .gen file to a genind object...
##
##
## File description:  genepop file generated from C_impar_filtered.u.snps.phy.
##
## ...done.
##
##
##  Converting data from a Genepop .gen file to a genind object...
##
##
## File description:  genepop file generated from M_bondari_filtered.u.snps.phy.
##
## ...done.
##
##
##  Converting data from a Genepop .gen file to a genind object...
##
##
## File description:  genepop file generated from M_ypsilon_filtered.u.snps.phy.
##
## ...done.
```

```
sample_info = read.csv("sample_info_new_WGA.csv")

pop_locations = read.csv("pop_locations.csv")
```

Now let's use DAPC to find clusters. We manually selected number of clusters according to variation in BIC for different number of clusters. Basically, we selected the number of groups right before a sharp increase in BIC. In the case of **Anchylorhynchus**, number of clusters was ever decreasing. We selected 4 clusters because that is the point in which the curve seems to start flattening.

This resulted in the following number of clusters:

```
nclusters = c(Anchylorhynchus = 4, Andranthobius = 1, C_impar = 3,
    M_bondari = 2, M_ypsilon = 2)
```

The following runs find.clusters (we first ran interactively to get the number of clusters above). To run interactively, one needs to change `choose.n.clust` to `FALSE`.

```
dapcs = lapply(taxa, function(x) {
    tryCatch(expr = {
        re = find.clusters(tab(genpops[[x]], freq = T, NA.method = "mean"),
            choose.n.clust = FALSE, pca.select = "percVar", perc.pca = 75,
            n.clust = nclusters[x])
        return(re)
    }, error = function(e) {
        samps = rownames(genpops[[x]]$tab)
        outvec = rep(1, length(samps))
        names(outvec) = samps
        re = list(grp = outvec)
        return(re)
    })
```

```
})
names(dapcs) = taxa
```

Now let's plot make plots PCAs including genetic clusters and local populations. We will save plots in a list and make a multipnale plot later.

```
titles = c(Anchylorhynchus = "Anchylorhynchus", Andranthobius = "Andranthobius",
    C_impar = "Celetes impar", M_bondari = "Microstrates bondari",
    M_ypsilon = "Microstrates ypsilon")
plots = list()

for (taxon in names(genpops)) {
    gendata = genpops[[taxon]]

    imputed = tab(gendata, freq = TRUE, NA.method = "mean")
    pca1 = dudi.pca(df = imputed, scale = FALSE, scannf = F,
        nf = 20)

    xrange = range(pca1$li$Axis1)
    yrange = range(pca1$li$Axis2)

    xrangeplot = xrange + c(-1, 1) * (xrange[2] - xrange[1]) *
        0.3
    yrangeplot = yrange + c(-1, 1) * (yrange[2] - yrange[1]) *
        0.3

    pc1_var = 100 * pca1$eig[1]/sum(pca1$eig)
    pc2_var = 100 * pca1$eig[2]/sum(pca1$eig)

    localities = gsub("^.*_", "", rownames(pca1$li)) %>% factor

    samples = gsub("_.*$", "", rownames(pca1$li))

    MDA = sample_info %>% filter(samplename_ipyrad %in% samples) %>%
        select(WGA) %>% unlist %>% factor(ordered = T, levels = c("TRUE",
        "FALSE"))

    plot_df = data.frame(sample = samples, locality = localities,
        cluster = dapcs[[taxon]]$grp, MDA = MDA, PC1 = pca1$li[,
            1], PC2 = pca1$li[, 2])

    # Some groups have few data points, and stat_ellipse needs at
    # least 3 points.To make ellipses consistently, we will
    # double the number of points and put a small jitter. The
    # idea is to simply enclose populations, not to be
    # statistically accurate.

    ellipse_df = rbind(plot_df, plot_df) %>% mutate(PC1 = PC1 +
        rnorm(n = length(PC1), mean = , sd = 0.1), PC2 = PC2 +
        rnorm(n = length(PC2), mean = , sd = 0.1)) %>% select(locality,
        PC1, PC2)

    centroids = plot_df %>% group_by(cluster) %>% summarise(cent.PC1 = mean(PC1),
        cent.PC2 = mean(PC2)) %>% right_join(plot_df)
```

```
    if (taxon == "Anchylorhynchus") {
        ellipses = NULL
    } else {
        ellipses = stat_ellipse(aes(x = PC1, y = PC2, group = locality),
            linetype = "dashed", data = ellipse_df, type = "t",
            color = "grey30")
    }
    p = ggplot(plot_df) + ellipses + geom_segment(aes(x = PC1,
        y = PC2, xend = cent.PC1, yend = cent.PC2), data = centroids) +
        geom_point(aes(x = PC1, y = PC2, color = MDA)) + xlab(paste("PC1 (",
        sprintf("%.1f", pc1_var), "%)", sep = "")) + ylab(paste("PC2 (",
        sprintf("%.1f", pc2_var), "%)", sep = "")) + theme_tufte() +
        theme(panel.border = element_rect(colour = "black", fill = NA)) +
        scale_colour_manual(values = brewer.pal(n = 3, name = "RdYlBu")[c(1,
            3)], name = "", labels = c(`FALSE` = "gDNA", `TRUE` = "MDA")) +
        ggtitle(bquote(italic(.(titles[taxon])))) + scale_y_continuous(labels = function(x) sprintf("%1
        x))

    plots[[taxon]] = p

}
```

```
## Joining, by = "cluster"
## Joining, by = "cluster"
## Joining, by = "cluster"
## Joining, by = "cluster"
## Joining, by = "cluster"
```

Now let's plot all of them together:

```
# extract legend. From:
# https://github.com/hadley/ggplot2/wiki/Share-a-legend-between-two-ggplot2-graphs
g_legend <- function(a.gplot) {
    tmp <- ggplot_gtable(ggplot_build(a.gplot))
    leg <- which(sapply(tmp$grobs, function(x) x$name) == "guide-box")
    legend <- tmp$grobs[[leg]]
    return(legend)
}

my_legend = g_legend(plots[[1]])

p = grid.arrange(plots[[1]] + theme(legend.position = "none"),
    plots[[2]] + theme(legend.position = "none"), plots[[3]] +
        theme(legend.position = "none"), plots[[4]] + theme(legend.position = "none"),
    plots[[5]] + theme(legend.position = "none"), my_legend,
    ncol = 2)
```

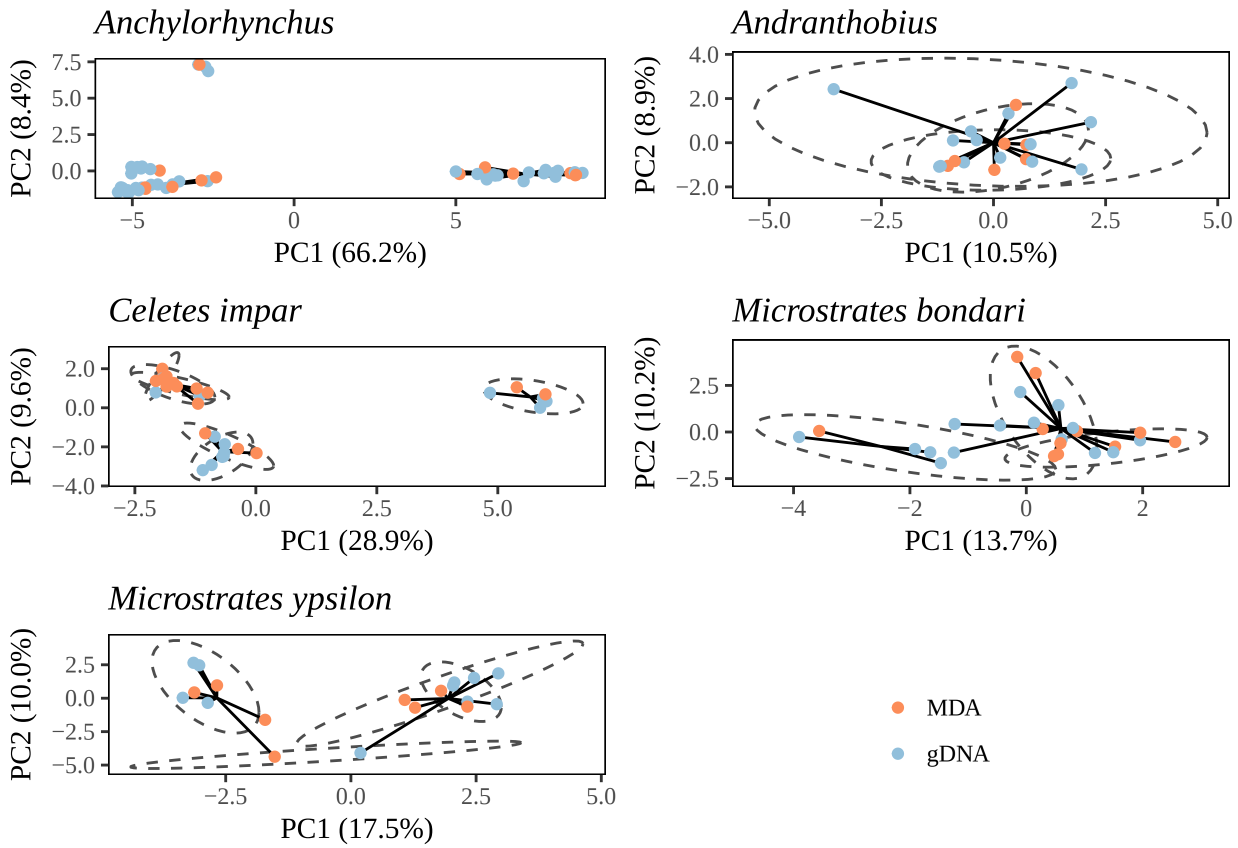


```
print(p)
```

```
## TableGrob (3 x 2) "arrange": 6 grobs
##   z     cells    name             grob
## 1 1 (1-1,1-1) arrange    gtable[layout]
## 2 2 (1-1,2-2) arrange    gtable[layout]
## 3 3 (2-2,1-1) arrange    gtable[layout]
## 4 4 (2-2,2-2) arrange    gtable[layout]
## 5 5 (3-3,1-1) arrange    gtable[layout]
## 6 6 (3-3,2-2) arrange gtable[guide-box]
```

```
ggsave(filename = "fig_DAPC.pdf", plot = p, device = "pdf", path = "plots/",
    width = 6.5, height = 8)
```

Now we will look at the effect of MDA on isolation by distance. We will use only **C. impar** only **Anchylorhynchus** is complicated and includes many species, while the other taxa have too few populations

We will start by defining a function that calculates pairwise FSTs. The standard function in hierfstat seems to calculate FST wrongly. I am using the function in BEDASSLE, which implements the "ratio of averages" as defined by Bhatia G., Patterson N., Sankararaman S., Price AL. 2013. Estimating and interpreting FST: The impact of rare variants. Genome Research 23:1514–1521. DOI: 10.1101/gr.154831.113.

```
get_pairwise_Fst = function(genmat, grp) {
    grp = factor(grp)
    if (length(levels(grp)) > 1) {

        allele.counts = apply(genmat@tab, 2, function(x) {
            pops = grp
            counts = tapply(x, pops, function(y) {
```

```
                sum(y, na.rm = T)
            })
            return(counts)
        })
        sample.sizes = apply(genmat@tab, 2, function(x) {
            pops = grp
            sizes = tapply(x, pops, function(y) {
                2 * sum(!is.na(y))
            })
            return(sizes)
        })

        Fst = calculate.all.pairwise.Fst(allele.counts, sample.sizes)
        colnames(Fst) = rownames(Fst) = levels(grp)
        # print(Fst)
        return(tidy(as.dist(Fst, upper = FALSE)))
    } else {
        print("Only one pop")
        return(matrix())
    }
}
```

Now let's calculate the pairwise geographical distances between populations

```
geodist = earth.dist(pop_locations[c("lon", "lat")], dist = FALSE)
rownames(geodist) = colnames(geodist) = pop_locations$population

geodist = geodist[sort(rownames(geodist)), sort(rownames(geodist))] %>%
    as.dist(upper = TRUE) %>% tidy() %>% transmute(population1 = item1,
    population2 = item2, geo_distance = distance)

head(geodist, n = 20)
```

population1	population2	geo_distance
P10	P1	158.68768
P11	P1	491.85846
P12	P1	566.00272
P13	P1	713.92707
P14	P1	742.79517
P15	P1	1034.82444
P16	P1	1084.98755
P17	P1	1114.62218
P18	P1	1021.10125
P19	P1	979.12169
P2	P1	245.63416
P20	P1	872.73557
P3	P1	354.94080
P4	P1	614.33708
P5	P1	656.78746
P6	P1	755.04150
P7	P1	534.14716
P8	P1	89.75459
P9	P1	433.54472

population1	population2	geo_distance
P1	P10	158.68768

Now let's calculate FSTs for **C. impar** (all samples, only MDA and only gDNA) and save results in a table

```
FST = plyr::ldply(c("all", "MDA", "gDNA"), function(y) {
    samples = rownames(genpops$C_impar@tab)
    pops = gsub("^.+_", "", samples)
    samples = gsub("_.+$", "", samples)

    if (y == "all") {
        return(get_pairwise_Fst(genpops$C_impar, pops) %>% mutate(method = y))
    } else if (y == "MDA") {
        samples = sample_info %>% filter(WGA == TRUE, samplename_ipyrad %in%
            samples) %>% tidyr::unite("sample_pop", samplename_ipyrad,
            population) %>% select(sample_pop) %>% unlist
        pops = gsub("^.+_", "", samples)
        return(get_pairwise_Fst(genpops$C_impar[samples], pops) %>%
            mutate(method = y))
    } else {
        samples = sample_info %>% filter(WGA == FALSE, samplename_ipyrad %in%
            samples) %>% tidyr::unite("sample_pop", samplename_ipyrad,
            population) %>% select(sample_pop) %>% unlist
        pops = gsub("^.+_", "", samples)
        return(get_pairwise_Fst(genpops$C_impar[samples], pops) %>%
            mutate(method = y))
    }



})

FST = FST %>% transmute(method, population1 = item1, population2 = item2,
    FST = distance)

FST
```

method	population1	population2	FST
all	P4	P2	0.2089811
all	P5	P2	0.2222732
all	P6	P2	0.2385589
all	P7	P2	0.1059134
all	P9	P2	0.4631936
all	P5	P4	0.0400003
all	P6	P4	0.0253863
all	P7	P4	0.1725187
all	P9	P4	0.4779835
all	P6	P5	0.0576042
all	P7	P5	0.1998439
all	P9	P5	0.5041844
all	P7	P6	0.2049786
all	P9	P6	0.4988504
all	P9	P7	0.4220915

method	population1	population2	FST
MDA	P4	P2	0.2717992
MDA	P5	P2	0.1990030
MDA	P6	P2	0.2687741
MDA	P7	P2	0.1146292
MDA	P9	P2	0.4539007
MDA	P5	P4	0.0565881
MDA	P6	P4	0.0731253
MDA	P7	P4	0.2066143
MDA	P9	P4	0.5209841
MDA	P6	P5	0.0506373
MDA	P7	P5	0.1942737
MDA	P9	P5	0.5030831
MDA	P7	P6	0.2049786
MDA	P9	P6	0.5265207
MDA	P9	P7	0.4353757
gDNA	P4	P2	0.2126439
gDNA	P5	P2	0.1940448
gDNA	P9	P2	0.4703934
gDNA	P5	P4	0.1007167
gDNA	P9	P4	0.4649076
gDNA	P9	P5	0.4994800

Now let's join geographical distance and linearize FST:

```
FST_plot = FST %>% left_join(geodist) %>% mutate(FST = (FST)/(1 -
    FST))
```

```
## Joining, by = c("population1", "population2")
```

```
FST_plot
```

method	population1	population2	FST	geo_distance
all	P4	P2	0.2641923	369.54998
all	P5	P2	0.2857986	411.66486
all	P6	P2	0.3132993	512.44438
all	P7	P2	0.1184599	326.76142
all	P9	P2	0.8628690	292.17121
all	P5	P4	0.0416670	84.64655
all	P6	P4	0.0260475	192.69093
all	P7	P4	0.2084865	289.63476
all	P9	P4	0.9156484	425.00440
all	P6	P5	0.0611253	112.65044
all	P7	P5	0.2497561	252.74804
all	P9	P5	1.0168790	406.63874
all	P7	P6	0.2578278	289.98861
all	P9	P6	0.9954123	454.73884
all	P9	P7	0.7303777	165.08018
MDA	P4	P2	0.3732477	369.54998
MDA	P5	P2	0.2484442	411.66486
MDA	P6	P2	0.3675664	512.44438
MDA	P7	P2	0.1294703	326.76142
MDA	P9	P2	0.8311688	292.17121

method	population1	population2	FST	geo_distance
MDA	P5	P4	0.0599824	84.64655
MDA	P6	P4	0.0788944	192.69093
MDA	P7	P4	0.2604210	289.63476
MDA	P9	P4	1.0876133	425.00440
MDA	P6	P5	0.0533383	112.65044
MDA	P7	P5	0.2411162	252.74804
MDA	P9	P5	1.0124090	406.63874
MDA	P7	P6	0.2578278	289.98861
MDA	P9	P6	1.1120247	454.73884
MDA	P9	P7	0.7710893	165.08018
gDNA	P4	P2	0.2700734	369.54998
gDNA	P5	P2	0.2407638	411.66486
gDNA	P9	P2	0.8881939	292.17121
gDNA	P5	P4	0.1119966	84.64655
gDNA	P9	P4	0.8688363	425.00440
gDNA	P9	P5	0.9979222	406.63874

Now let's plot:

```
p = ggplot(FST_plot, aes(x = geo_distance, y = FST, color = method)) +
    geom_point() + geom_smooth(method = lm, se = FALSE) + theme_tufte() +
    theme(panel.border = element_rect(colour = "black", fill = NA)) +
    scale_colour_manual(values = c("black", brewer.pal(n = 3,
        name = "RdYlBu")[c(3, 1)]), name = "") + ylab(bquote("F"["ST"]/(1 -
    "F"["ST"]))) + xlab("Geographical distance (km)")

print(p)
```

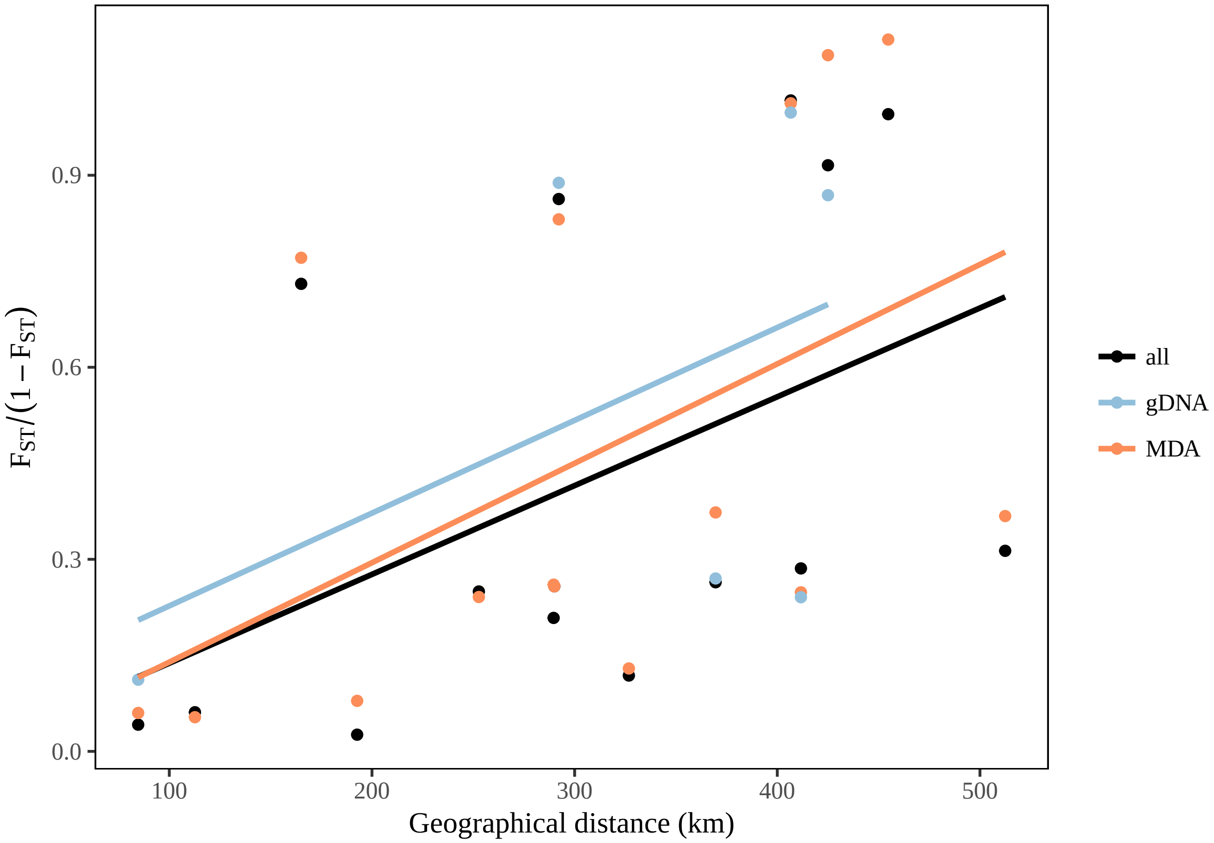


```
ggsave(filename = "fig_Mantel.pdf", plot = p, device = "pdf",
    path = "plots/")
```

```
## Saving 6.5 x 4.5 in image
```

Finally, let's do Mantel tests and see if results are different between methods:

```
for (method in c("all", "gDNA", "MDA")) {

    FST_mat = FST_plot %>% filter(!(!method == method)) %>% dcast(formula = population2 ~
        population1, value.var = "FST") %>% (function(x) {
        rownames(x) = x[[1]]
        return(x[-1])
    }) %>% as.matrix

    pops = base::union(rownames(FST_mat), colnames(FST_mat)) %>%
        sort

    FST_dist = matrix(NA, nrow = length(pops), ncol = length(pops),
        dimnames = list(pops, pops))

    FST_dist[t(combn(pops, 2))] = FST_mat[t(combn(pops, 2))]

    FST_dist = FST_dist %>% t %>% as.dist



    geo_mat = FST_plot %>% filter(!(!method == method)) %>% dcast(formula = population2 ~
```

```r
        population1, value.var = "geo_distance") %>% (function(x) {
        rownames(x) = x[[1]]
        return(x[-1])
    }) %>% as.matrix

    geo_dist = matrix(NA, nrow = length(pops), ncol = length(pops),
        dimnames = list(pops, pops))

    geo_dist[t(combn(pops, 2))] = geo_mat[t(combn(pops, 2))]

    geo_dist = geo_dist %>% t %>% as.dist

    mantel_res = mantel(geo_dist, FST_dist)
    print(method)
    print(mantel_res)
}
```

```
## Aggregation function missing: defaulting to length
## Aggregation function missing: defaulting to length

## 'nperm' >= set of all permutations: complete enumeration.

## Set of permutations < 'minperm'. Generating entire set.

## [1] "all"
##
## Mantel statistic based on Pearson's product-moment correlation
##
## Call:
## mantel(xdis = geo_dist, ydis = FST_dist)
##
## Mantel statistic r:     1
##       Significance: 0.066667
##
## Upper quantiles of permutations (null model):
##   90%   95% 97.5%   99%
## 0.167 1.000 1.000 1.000
## Permutation: free
## Number of permutations: 719

## Aggregation function missing: defaulting to length

## Aggregation function missing: defaulting to length

## 'nperm' >= set of all permutations: complete enumeration.

## Set of permutations < 'minperm'. Generating entire set.

## [1] "gDNA"
##
## Mantel statistic based on Pearson's product-moment correlation
##
## Call:
## mantel(xdis = geo_dist, ydis = FST_dist)
##
## Mantel statistic r:     1
##       Significance: 0.066667
##
```

```
## Upper quantiles of permutations (null model):
##   90%   95% 97.5%   99%
## 0.167 1.000 1.000 1.000
## Permutation: free
## Number of permutations: 719
```

```
## Aggregation function missing: defaulting to length
```

```
## Aggregation function missing: defaulting to length
```

```
## 'nperm' >= set of all permutations: complete enumeration.
```

```
## Set of permutations < 'minperm'. Generating entire set.
```

```
## [1] "MDA"
##
## Mantel statistic based on Pearson's product-moment correlation
##
## Call:
## mantel(xdis = geo_dist, ydis = FST_dist)
##
## Mantel statistic r:     1
##       Significance: 0.066667
##
## Upper quantiles of permutations (null model):
##   90%   95% 97.5%   99%
## 0.167 1.000 1.000 1.000
## Permutation: free
## Number of permutations: 719
```

This lists number of samples per population Table S2:

```
for (taxon in taxa) {
    print(taxon)
    print(sample_info[sapply(sample_info$samplename_ipyrad, function(x) any(grepl(x,
        rownames(genpops[[taxon]]@tab)))), ] %>% select(population,
        WGA) %>% table)
}
```

```
## [1] "Anchylorhynchus"
##           WGA
## population FALSE TRUE
##         P1     4    1
##         P2     8    2
##         P3     4    0
##         P4     0    3
##         P5     9    2
##         P6     6    2
##         P7     6    1
##         P8     3    1
##         P9     5    1
## [1] "Andranthobius"
##           WGA
## population FALSE TRUE
##         P1     1    4
##         P2     0    0
##         P3     7    2
##         P4     0    0
```

```
##         P5     0    0
##         P6     0    0
##         P7     0    0
##         P8     6    1
##         P9     0    0
## [1] "C_impar"
##           WGA
## population FALSE TRUE
##         P1     0    0
##         P2     5    1
##         P3     0    0
##         P4     3    2
##         P5     2    5
##         P6     0    4
##         P7     0    4
##         P8     0    0
##         P9     4    2
## [1] "M_bondari"
##           WGA
## population FALSE TRUE
##         P1     8    2
##         P2     0    0
##         P3     2    5
##         P4     0    0
##         P5     0    0
##         P6     0    0
##         P7     0    0
##         P8     5    4
##         P9     0    0
## [1] "M_ypsilon"
##           WGA
## population FALSE TRUE
##         P1     0    0
##         P2     0    0
##         P3     0    0
##         P4     1    2
##         P5     5    2
##         P6     0    0
##         P7     0    2
##         P8     0    0
##         P9     5    2
```
