# Supplementary figures and images for: Whole-genome amplification in double-digest RADseq results in adequate libraries but fewer sequenced loci
Source: PeerJ. 2018 Jul 17;6:e5089. doi: 10.7717/peerj.5089 (PMC6054070; doi:10.7717/peerj.5089)

*Anchylorhynchus*

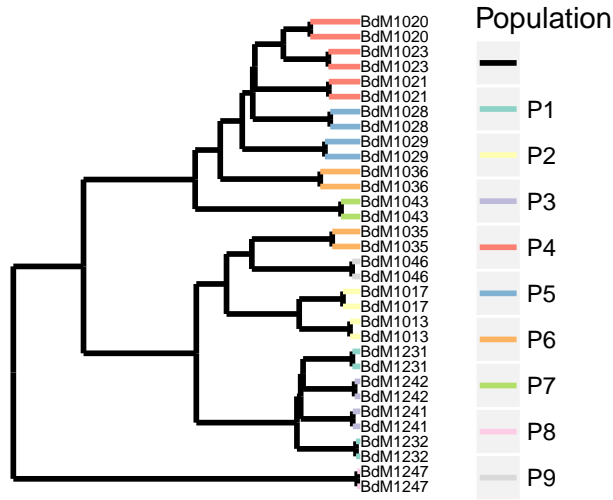

*Andranthobius*

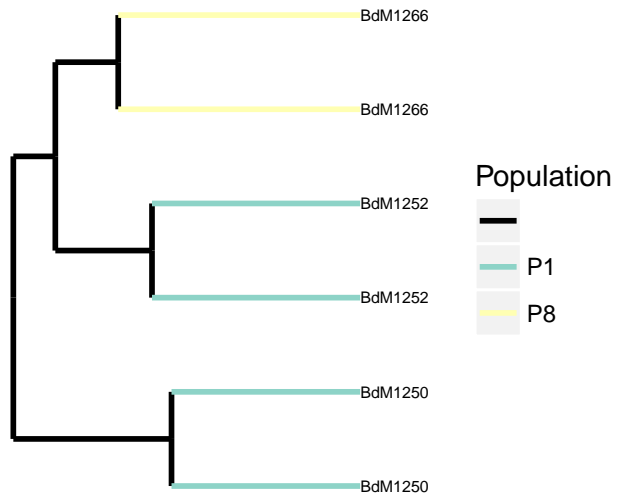

*C. impar*

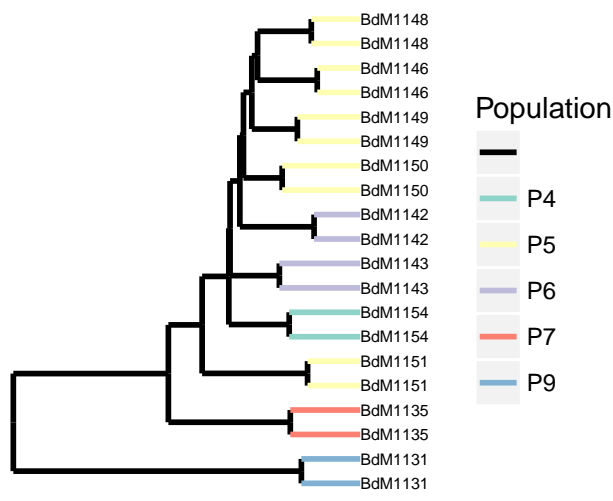

*M. bondari*

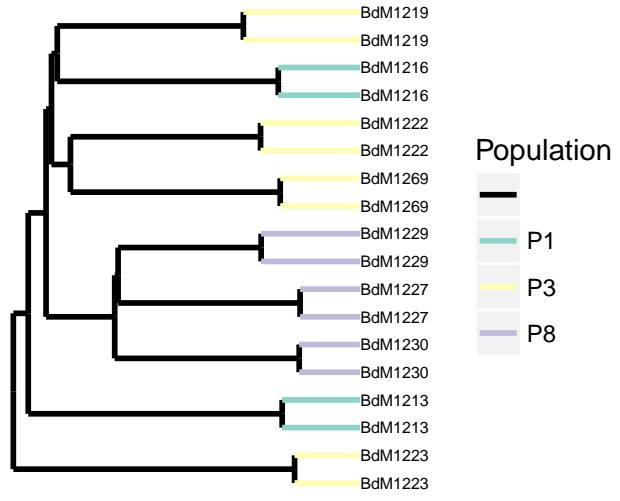

*M. ypsilon*

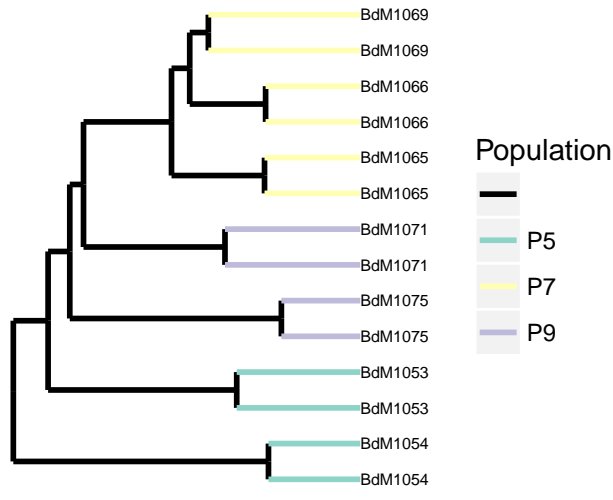

Supplement: Supplemental Information 9 — Neighbor-joining dendograms based on pairwise genetic distances for each taxon always cluster individuals and individuals are often clustered by population. [file peerj-06-5089-s009.pdf]

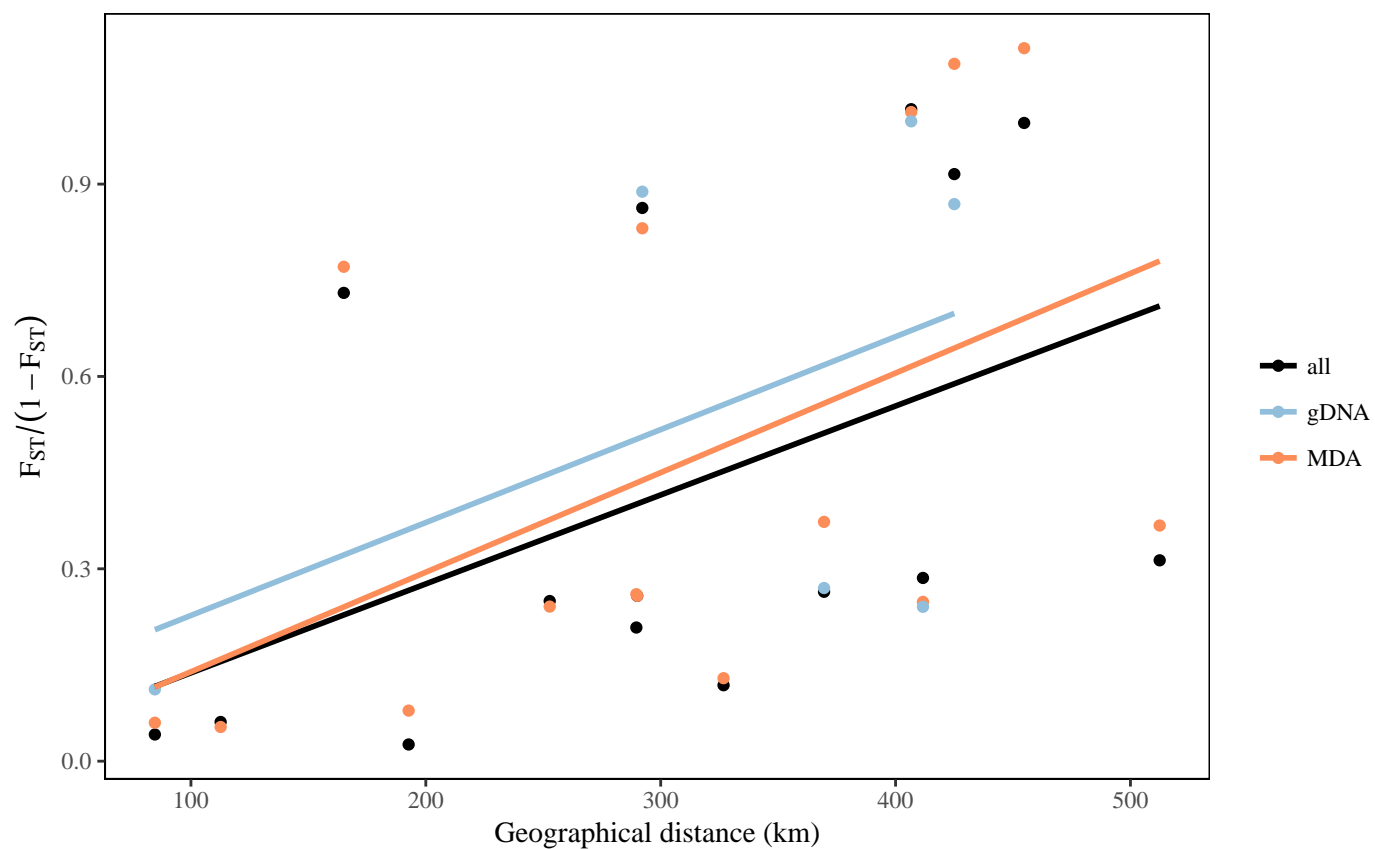

Supplement: Supplemental Information 10 — Patterns of isolation by distance in Celetes impar inferred from the whole dataset, gDNA libraries only and MDA libraries only. Two populations did not retain any gDNA samples in the more stringently filtered dataset, resulting in fewer comparisons here. The slopes using the three different datasets are very similar. [file peerj-06-5089-s010.pdf]
